# First record of a tomistomine crocodylian from Australia

**DOI:** 10.1038/s41598-021-91717-y

**Published:** 2021-06-09

**Authors:** Jorgo Ristevski, Gilbert J. Price, Vera Weisbecker, Steven W. Salisbury

**Affiliations:** 1 School of Biological Sciences, The University of Queensland, Brisbane, QLD 4072 Australia; 2 School of Earth and Environmental Sciences, The University of Queensland, Brisbane, QLD 4072 Australia; 3 College of Science and Engineering, Flinders University, Bedford Park, SA 5042 Australia

**Keywords:** Evolution, Palaeontology, Phylogenetics, Taxonomy

## Abstract

Based on the known fossil record, the majority of crocodylians from the Cenozoic Era of Australia are referred to the extinct clade Mekosuchinae. The only extant crocodylians in Australia are two species of *Crocodylus*. Hence, the viewpoint that *Crocodylus* and mekosuchines have been the only crocodylians inhabiting Australia during the Cenozoic has remained largely undisputed. Herein we describe Australia’s first tomistomine crocodylian, *Gunggamarandu maunala* gen. et sp. nov., thus challenging the notion of mekosuchine dominance during most of the Cenozoic. The holotype specimen of *Gunggamarandu maunala* derives from the Pliocene or Pleistocene of south-eastern Queensland, marking the southern-most global record for Tomistominae. *Gunggamarandu maunala* is known from a large, incomplete cranium that possesses a unique combination of features that distinguishes it from other crocodylians. Phylogenetic analyses place *Gunggamarandu* in a basal position within Tomistominae, specifically as a sister taxon to *Dollosuchoides* from the Eocene of Europe. These results hint at a potential ghost lineage between European and Australian tomistomines going back more than 50 million years. The cranial proportions of the *Gunggamarandu maunala* holotype specimen indicate it is the largest crocodyliform yet discovered from Australia.

## Introduction

Crocodylian evolution from the Australian Cenozoic is mainly characterized by the taxonomically rich and morphologically diverse crocodylian clade Mekosuchinae^1–7^. As currently understood, Australia’s mekosuchine fossil record spans from the Eocene^1,2,8,9^ to the Pleistocene^7,10–13^, an inference based on remains collected across the continent, primarily the eastern half^2–4,14^. No mekosuchines are known to have survived in Australia after the late Pleistocene, thus leaving two species of *Crocodylus*—*C. johnstoni* (the sole extant crocodylian endemic to Australia) and *C. porosus*—as the only surviving crocodylians on the continent. Fossils attributable to *Crocodylus* in Australia have been reported from Pliocene^6,11,15,16^ and Pleistocene^17^ deposits. Therefore, the overarching perception in the past three decades has been that only two crocodylian lineages—the Australasian endemic Mekosuchinae and the cosmopolitan *Crocodylus*—have inhabited Australia during the past 66 million years.

That an endemic crocodylian radiation dominated Australia during the Cenozoic is in sharp contrast to the rest of the world. The Cenozoic crocodylian faunas on other continents (except Antarctica) were generally represented by multiple eusuchian lineages, which still holds true today in the Americas, Asia, and to a lesser extent Africa. Moreover, Africa, Asia, Europe, and the Americas even hosted non-eusuchian crocodyliforms (i.e., sebecosuchians and tethysuchians) during the Paleogene and Neogene^18–22^. However, there are indications that the higher-clade crocodylian diversity in Australia was greater than previously perceived. Several studies^2,6,7,23,24^ suggest that the Miocene Australian taxon *Harpacochampsa camfieldensis* is probably not a mekosuchine, although its precise phylogenetic affinities are uncertain.

**Figure 1:**
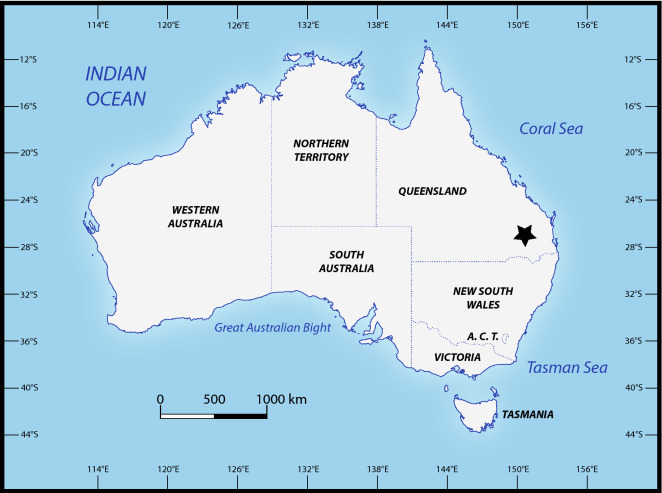
Map of Australia. The black star indicates the Darling Downs region, where the *Gunggamarandu maunala* gen. et sp. nov. holotype specimen, QMF14.548, was discovered. This map was drawn by J. R. in Adobe Illustrator CC 2021 (https://www.adobe.com/products/illustrator.html) based on data modified from SimpleMappr (https://www.simplemappr.net/).

In this study, we describe the first unambiguous tomistomine crocodylian from Australia—a representative of a clade hitherto unreported from this part of the world (Fig. 1).

## Results

### Systematic paleontology

CROCODYLOMORPHA Hay^25^ (sensu Nesbitt^26^)

CROCODYLIFORMES Hay ^25^ (sensu Sereno et al.^27^)

MESOEUCROCODYLIA Whetstone & Whybrow^28^ (sensu Sereno et al.^27^)

EUSUCHIA Huxley^29^

CROCODYLIA Gmelin ^30^ (sensu Clark in Benton & Clark^31^)

TOMISTOMINAE Kälin ^32^ (sensu Brochu^5^)

*GUNGGAMARANDU* GEN. NOV

**Type species.** *Gunggamarandu maunala* gen. et sp. nov.

**Etymology.** The name of the genus incorporates words from Barunggam and Wakka Wakka, the languages spoken by the people of the Barunggam and Wakka Wakka nations of the Darling Downs, south-eastern Queensland. The generic name *Gunggamarandu* (pronounced [gung-ga-ma-ran-du] with the emphasis on the first syllable only) is a combination of the Barunggam and Wakka Wakka words *guƞ* or *gung* meaning ‘water’, ‘waterhole’ or ‘river’, and *gamarandu*, the Barunggam word for ‘boss’ or ‘owner’ (see Holmer^33^).

**Generic diagnosis.** Large-sized tomistomine crocodylian characterized by the following unique combination of features (autapomorphies indicated with *): (1) proportionally large supratemporal fenestrae, occupying over 48% of the cranial table surface at maturity; (2) frontoparietal fossa with no anteromedial extension on cranial table; (3) broad frontal bearing a conspicuous concavity spread widely over most of the frontal’s dorsal surface at maturity; (4) deep sub-triangular concavity spread widely over most of the parietal’s posterodorsal surface; (5) minimum width of parietal between the supratemporal fenestrae is 10% or less the maximum cranial table width; (6) the bar forming the posterior border of the supratemporal fenestra is slender, with the minimum thickness being less than 8% of the maximum cranial table width; (7) very large and widely spaced postoccipital processes of the supraoccipital, separated from each other by a width of ~ 35% the total width of the supraoccipital; (8) occipital surface of the supraoccipital convex and without a nuchal crest*; (9) foramen magnum and myelencephalon portion of the brain endocast obround in cross-section*; (10) pterygoid process of the quadrate with evident occipital exposure ventrolateral to the otoccipital; (11) ventral surface of quadrate with hypertrophied crests A and B’.

*GUNGGAMARANDU* *MAUNALA* GEN. ET SP. NOV.

**Etymology.** The specific epithet *maunala* (pronounced [mau-nala] with the emphasis on the first syllable only) is a combination from the Barunggam words *mau*, meaning ‘head’, and *nala*, meaning ‘hole’ (see Holmer^33^). Given in reference to the large supratemporal openings on the skull.

**Holotype.** QMF14.548 (Queensland Museum, Brisbane, Queensland, Australia [F, fossil]), incomplete cranium.

**Type locality and horizon.** Unknown locality on the Darling Downs, south-eastern Queensland. The fossil-bearing localities of the Darling Downs region are generally divided into two major areas, the eastern Darling Downs and the western Darling Downs^34^. The western Darling Downs is predominantly Pliocene deposits^35–37^, whereas the eastern Darling Downs is predominantly Pleistocene^36,38,39^. The precise age of QMF14.548 is uncertain, as it could be either Pliocene or Pleistocene.

**Specific diagnosis.** Because *Gunggamarandu maunala* is the only know species within its genus, the generic and specific diagnoses are the same.

### General remarks and morphological description for QMF14.548

**Figure 2:**
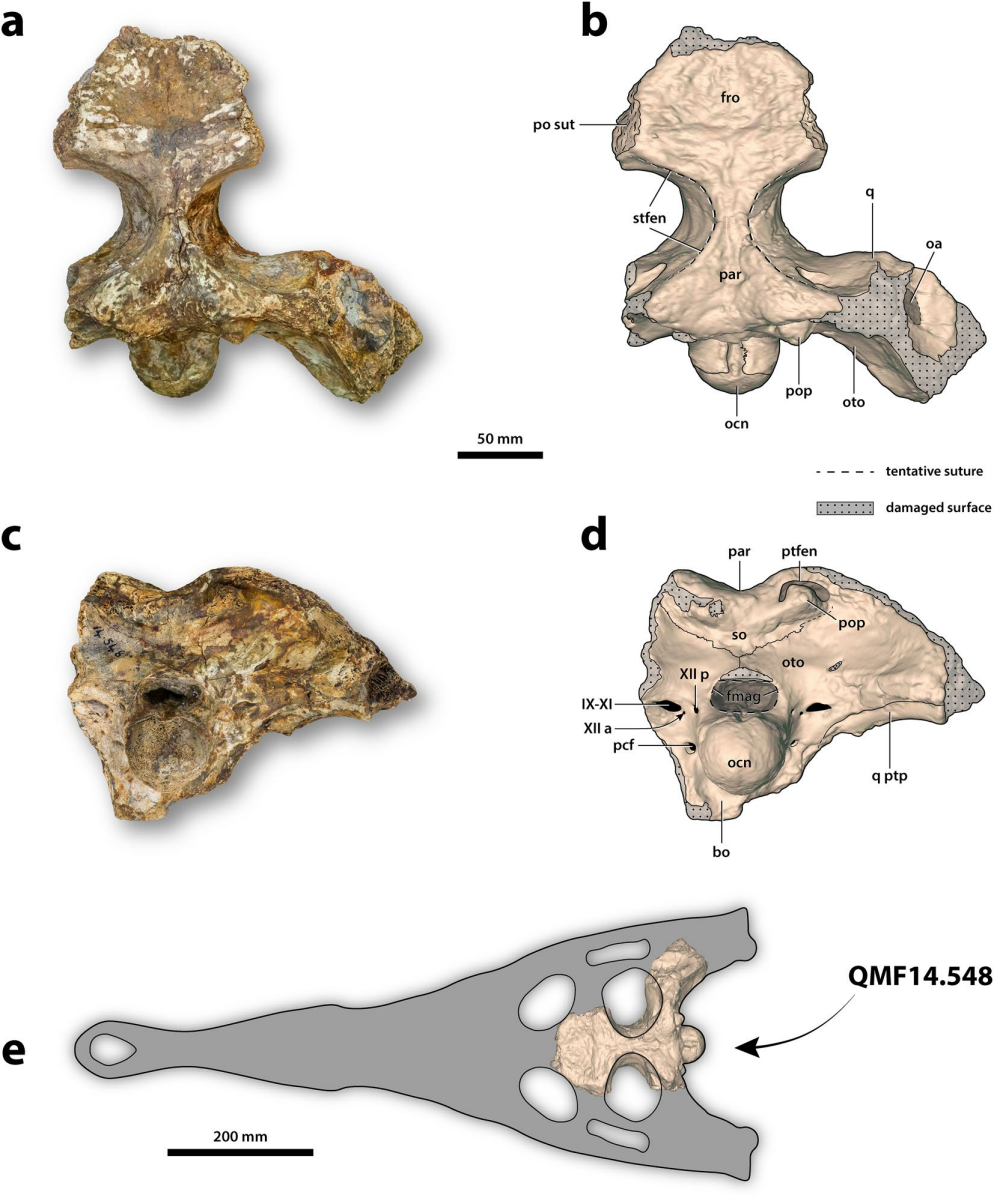
*Gunggamarandu maunala* gen. et sp. nov., QMF14.548, holotype. (**a**) Photograph, and (**b**) annotated digital model of the cranium in dorsal views. (**c**) Photograph, and (**d**) annotated digital model of the cranium in occipital views. (**e**) Hypothetical outline of the skull of *Gunggamarandu maunala* in dorsal view, with QMF14.548 depicted in its corresponding position. The hypothetical skull outline of *Gunggamarandu maunala* in (**e**) is based on the skulls of *Dollosuchoides densmorei* and *Kentisuchus spenceri*. bo, basioccipital; fmag, foramen magnum; fro, frontal; IX-XI, foramen for glossopharyngeal, vagus, and accessory nerves (metotic foramen); oa, otic aperture; ocn, occipital condyle; oto, otoccipital; par, parietal; pcf, posterior carotid foramen; po sut, sutural surface for articulation with the postorbital; pop, postoccipital process of the supraoccipital; ptfen, posttemporal fenestra; q, quadrate; q ptp, quadrate pterygoid process; so, supraoccipital; stfen, supratemporal fenestra; XII a, anterior hypoglossal foramen; XII p, posterior hypoglossal foramen. This figure was created by J. R. in Adobe Illustrator CC 2021 (https://www.adobe.com/products/illustrator.html).

**Figure 3:**
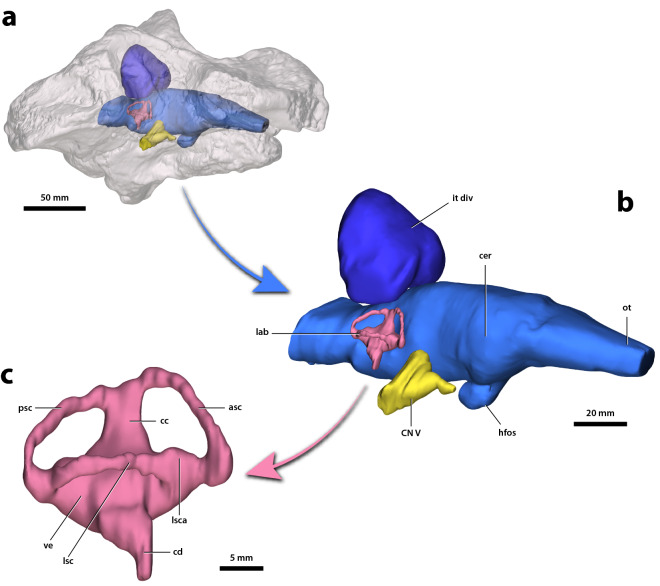
*Gunggamarandu maunala* gen. et sp. nov., QMF14.548, holotype. (**a**) Transparent digital model of the cranium in oblique right lateral view, exposing the digitally segmented endocranial structures. (**b**) Digitally segmented endocranial structures in oblique right lateral view. (**c**) Right endosseous labyrinth in lateral view. asc, anterior semicircular canal (endocast); cc, common crus (endocast); cd, cochlear duct (endocast); cer, cerebrum (endocast); CN V, trigeminal nerve canal; hfos, hypophyseal fossa (endocast); it div, intertympanic diverticulum; lab, endosseous labyrinth; lsc, lateral semicircular canal (endocast); lsca, ampulla of lateral semicircular canal (endocast); ot, olfactory tract (endocast); psc, posterior semicircular canal (endocast); ve, vestibule (endocast). This figure was created by J. R. in Adobe Illustrator CC 2021 (https://www.adobe.com/products/illustrator.html).

The holotype specimen of *Gunggamarandu maunala*, QMF14.548 (Figs. 2 and 3), is an incomplete cranium that belonged to a large crocodylian. Details surrounding its discovery are scant. According to its museum record, QMF14.548 was accessioned into the QM collections on 8 January1914. Museum records further note that QMF14.548 was originally part of the ‘old collection’ (see Mather^40^), which indicates that the specimen was discovered no later than the 1870s. During the late nineteenth century, intensive prospecting for fossils was undertaken on behalf of the QM in order to establish its collection. Prospecting and collecting efforts were focused primarily on the Darling Downs, with thousands of fossils coming to the museum from that region. Therefore, given its accession history, it is likely that QMF14.548 is from the Darling Downs. While QMF14.548 has never been formally described prior to this study, it was the subject of a taxonomic discussion by Salisbury et al.^41^ who tentatively suggested it may represent a gavialoid. Salisbury et al.^41^ also suggested that the specimen could be Pleistocene in age as its preservation style appears to be consistent with vertebrate fossils from the eastern Darling Downs. The eastern Darling Downs provenance of the specimen was further corroborated by the late Dr. Alan Bartholomai, former Director of the QM in discussions with Dr. Ralph Molnar in 1995 (J. Ristevski, S. W. Salisbury and R. E. Molnar, pers. comm. 2 February 2021). Although we are relatively confident that the specimen comes from the Darling Downs, without any definitive provenance data we feel it is prudent to regard its age as either Pliocene or Pleistocene.

The external surfaces of QMF14.548 are affected by small, fine cracks with few wider breakages, and some portions are covered by matrix (especially at the anterior of the braincase). Most (but not all) sutures are either very difficult to discern or not visible externally on the cranium. Additionally, the endocranial cavities and cranial foramina are partially filled with matrix of grey, pebbly conglomerate (poorly sorted clasts in a silty matrix). Consequently, not all endocranial elements could be digitally segmented because of the matrix-filled cranial cavities. Externally, the specimen has a russet color interspersed with beige, gray and ebony splotches. Despite the superficial fractures, there is no, or only insignificant degree of plastic deformation; therefore, the specimen maintains the integrity of its three-dimensional form.

The following elements are preserved on the cranium: partial frontal, parietal, supraoccipital, laterosphenoids, partial quadrates (mostly the right quadrate), partial otoccipitals, and partial basioccipital. A very small section of the right squamosal is still present, forming the posterior margin of the otic aperture. Even though the postorbitals are missing, traces of their sutural surfaces are visible on the lateral margins of the frontal and parietal. Modest portions of the pterygoids are also visible, although not to a degree that allows a thorough assessment of their morphology. The presence of the prootics and basisphenoid may also be inferred, although no description of those elements can be provided. What follows is an abbreviated description of QMF14.548. A detailed description of the specimen, complemented by additional figures, is given in Supplemental Document S1.

The supratemporal fenestrae are mostly complete and occupy much of the cranial table (Fig. 2a,b). The dimensions of each supratemporal fenestra are ~ 86 mm in anteroposterior length, and ~ 70 mm of preserved transverse width; however, since the lateral margins of the fenestrae are missing, the total transverse width of each fenestra would have been greater than 70 mm. The frontoparietal fossae (sensu Holliday et al.^42^) do not extend anteromedially on the cranial table (character 210, state 0).

The foramen magnum is complete and has a distinctly obround outline (Fig. 2c,d). Although an elongated foramen magnum is not uncommon in tomistomines^43^, no crocodylian that we are aware of has a foramen magnum with an outline like that of *G. maunala*. The dimensions of the foramen magnum are ~ 44 mm in transverse width, and ~ 22 mm in dorsoventral height.

The frontal (Fig. 2a,b) is relatively well preserved, although missing its anterior process. The frontal’s dorsal surface is broad, bearing a conspicuous medial concavity that is widely spread over it. Such shallow and wide concavities on the frontal are present in mature specimens of certain basal tomistomines (e.g., *Dollosuchoides densmorei*, *Gavialosuchus eggenburgensis*, *Kentisuchus*^44^, *Maroccosuchus zennaroi*^45^, *Megadontosuchus arduini*^46^, “*Tomistoma*” *calaritanum*, and “*Tomistoma*” *gaudense*^43^). The dorsal surface of the frontal is mainly ornamented with grooves that are delimited by low ridges.

The parietal (Fig. 2) is well preserved and occupies the posteromedial part of the cranial table. Although the parietal is a large element that comprises a significant portion of the cranium, its dorsal surface is proportionally narrow because of the large supratemporal fenestrae. The interfenestral and postfenestral bars are highly constricted (character 208, state 0, and character 209, state 2). The most intriguing feature of the parietal of *G. maunala* is the deep and sub-triangular medial concavity. A sub-triangular concavity like that of *G. maunala* is also present in the basal tomistomine *Kentisuchus spenceri* from the early Eocene of England^47^.

It is undetermined if the supraoccipital is exposed dorsally on the cranial table (Fig. 2). Unique to *G. maunala*, the occipital surface of the supraoccipital is wide, markedly convex and without a nuchal crest. The postoccipital processes of the supraoccipital (of which the right process is complete, whereas the left is partial) are highly enlarged and widely separated from each other (character 229, state 1). Computed Tomographic (CT) scans reveal an expansive intertympanic diverticulum occupying the supraoccipital internally (Fig. 3a,b).

The otoccipitals (opistothics-exoccipitals) of QMF14.548 are only partially preserved, with the right otoccipital being the more complete (Fig. 2). The otoccipitals are in sutural contact dorsomedially to the foramen magnum, preventing the supraoccipital from contributing to the margins of the foramen. The dorsolateral portions of the occipital condyle neck are comprised by the otoccipitals, however, the otoccipitals do not contribute to the occipital condyle. Several cranial nerve and vasculature foramina open on the otoccipitals, which are the hypoglossal (cranial nerve XII) foramina, the common foramina for cranial nerves IX-XI and their associated vessels, and the posterior carotid foramina (Fig. 2c,d).

The basioccipital is only partially preserved, and observable in occipital aspect (Fig. 2c,d). It forms part of the occipital condyle neck and the entirety of the large and spherical occipital condyle. The basioccipital plate, although incomplete, is clearly oriented posteriorly (character 170, state 1).

Substantial portions of the quadrates are missing, with the right quadrate of QMF14.548 being by far the more complete of the two (Fig. 2). The right quadrate preserves a significant part of its dorsomedial-most portion (i.e., the quadrate dorsal primary head sensu Kley et al.^48^), its anterodorsal process, pterygoid process, and part of the quadrate body. The sutural relationships between the quadrate and its adjacent elements are not visible externally on the specimen. Some of the most conspicuous features on the ventral surface of the right quadrate are the hypertrophied crests A and B’ (sensu Iordansky^49^). In occipital view, the pterygoid process of the right quadrate is exposed ventral to the otoccipital (character 214, state 1; Fig. 2c,d).

The digitally segmented brain endocast of *G. maunala* (Fig. 3a,b) is mostly complete. The only missing portions of the endocast are the olfactory bulbs and the anterior half of the olfactory tract endocasts. The overall shape of the brain endocast is akin to those of some mesoeucrocodylian crocodyliforms, both extant and extinct^50–58^. When observed in lateral view, the endocast displays a smoothly sinusoidal dorsal contour with relatively unpronounced cephalic (the angle between the forebrain and midbrain, measured to be 149°) and pontine (the angle between the midbrain and hindbrain, measured to be 152°) flexures. Impressions of the occipital and ventral longitudinal dural venous sinuses are easily discernable. A standout feature of the brain endocast is the shape of the myelencephalon region of the rhombencephalon (which contained the remainder of the medulla oblongata, ventral longitudinal dural venous sinus and the occipital sinus). This portion of the endocast is dorsoventrally compressed and transversely elongated, with its cross-sectional outline mirroring the obround foramen magnum.

The trigeminal nerve (cranial nerve V) canals are the only cranial nerve canals that could be digitally segmented from QMF14.548 (Fig. 3a,b). These nerve canals are very large, with each of the two projecting laterally from the brain endocast at an angle of approximately 35°. Externally, each canal exits the braincase through its trigeminal foramen (more specifically, through the maxillomandibular foramen).

The endosseous labyrinths of the inner ears (sensu Witmer et al.^51^) could also be digitally segmented, with the right labyrinth being complete and virtually undistorted (Fig. 3). The vestibular apparatus has a sub-pyramidal shape and comprises the dorsal component of the endosseous labyrinth, whereas the cochlear duct extends lateroventrally from the aforementioned. Medially on the vestibular apparatus is the dorsally rising common crus, and the anterior and posterior semicircular canals meet at its dorsal end. The ampulla of the lateral semicircular canal has an obvious impression that is notable as a subtle dilation immediately anterior to the lateral semicircular canal (Fig. 3c).

### *Gunggamarandu* in context of Tomistominae, and differentiation from Gavialidae

Although many anatomical features of *G. maunala* are unknown, what is preserved of the holotype demonstrates that it is a tomistomine and is sufficiently distinct to warrant the erection of a new taxon. Two cranial features standout as unique to *G. maunala*. Of particular note is the supraoccipital with a markedly convex and wide occipital surface that lacks a nuchal crest. Likewise, the obround outline of the foramen magnum is hitherto unreported in a tomistomine.

The holotype cranium of *G. maunala* displays multiple features that it has in common with other tomistomines that also distinguish it from gavialids. (Here, Tomistominae is discussed in a phylogenetic context outside of Gavialidae.) A peculiar feature of *G. maunala* is the deep sub-triangular concavity on the parietal, that it shares with *K. spenceri*. No other currently described tomistomine possesses a parietal as deeply concave like that of *G. maunala* and *K. spenceri*. Besides *G. maunala*, very large and widely separated postoccipital processes of the supraoccipital (character 229, state 1) occur in some other tomistomines, such as *D. densmorei* (IRSNB R1748; see Brochu^47^) and *T. schlegelii* (e.g., TMM M-6342; also, see Sookias^59^). Similarly hypertrophied postoccipital processes of the supraoccipital also characterize gavialids, however, unlike tomistomines, the postoccipital processes of gavialids are positioned very close to each other (character 229, state 2; see Figs. S2.1E,F in Supplemental Document S2). Therefore, it seems that very large and closely spaced postoccipital processes of the supraoccipital is a synapomorphy of Gavialidae, but not Tomistominae. The very large size of the supratemporal fenestrae and the strong constriction of the interfenestral and postfenestral bars (character 208, state 0, and character 209, state 2) in *G. maunala* are common traits among long (i.e., longirostrine) and slender snouted crocodylians (or slender longirostrine sensu Drumheller and Wilberg^60^), including tomistomines (e.g., *D. densmorei*^47^; *Ga. eggenburgensis*^61^; *Toyotamaphimeia machikanensis*^62^). While the postfenestral bar (formed by the medial contact between the parietal and the medial process of the squamosal) is largely missing in QMF14.548, a small but adequate sliver remains of it to attest its slender nature. Finally, the capitate processes of the laterosphenoids of *G. maunala* are positioned posteriorly in relation to the olfactory foramen (character 166, state 1), which is the more common condition among crocodylians, including most tomistomines. In gavialids, the capitate processes tend to be positioned in line with the olfactory foramen (character 166, state 0).

### Phylogeny

**Figure 4:**
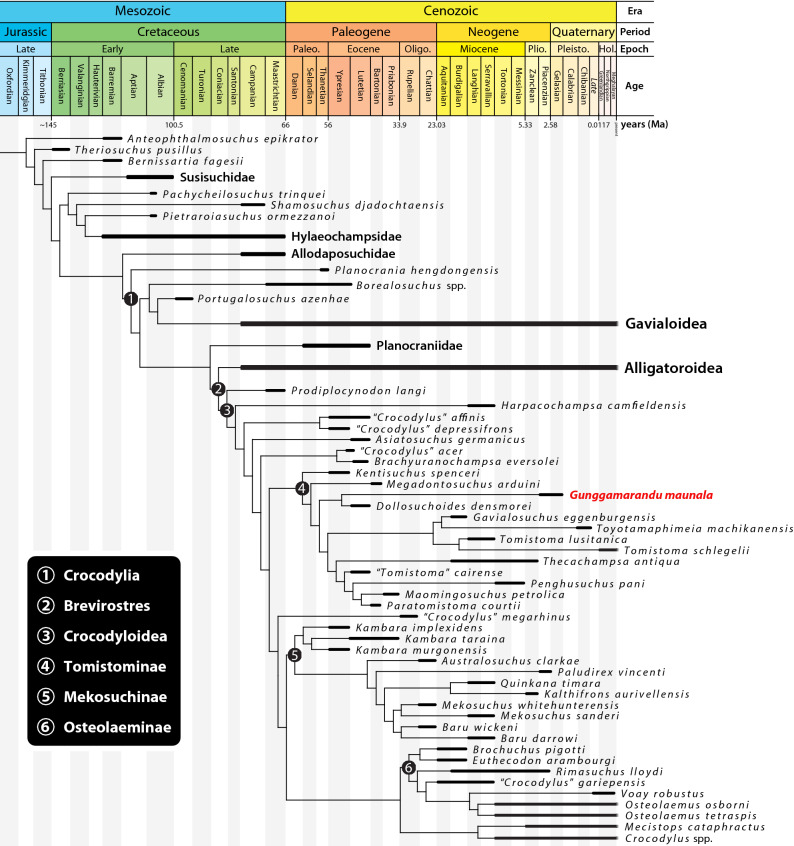
Time-calibrated phylogeny based on the strict consensus results from the analysis that used the Implied Weighting method, with the *k* (concavity constant) value set to 25.0 (k = 25). The first and last appearance dates for the taxa were acquired from the Paleobiology Database (https://paleobiodb.org) on 11 October 2020. The geologic dates are based on version 2020/03 of the International Chronostratigraphic Chart (https://stratigraphy.org/chart). For the complete strict consensus topology, nodal values, and more information on the phylogeny see Supplemental Document S2. This figure was created by J. R. in Adobe Illustrator CC 2021 (https://www.adobe.com/products/illustrator.html).

The phylogenetic affinities of *Gunggamarandu maunala* were tested in four phylogenetic analyses. The first analysis was run under an equal weighting search procedure, while the remaining three used implied weighting (see “Methods” below). Although the resulting topologies largely differ between the analyses (see Supplemental Document S2), the position of *Gunggamarandu* in the cladogram is remarkably consistent. The results (Fig. 4) support the interpretation of *Gunggamarandu* as a tomistomine crocodylian, and more intriguingly, one in a basal position within the clade. In all four analyses, *Gunggamarandu* is the sister taxon to *Dollosuchoides densmorei.* The *Gunggamarandu* + *Dollosuchoides* clade is characterized by two synapomorphies: a slender postfenestral bar, with a minimum thickness that is less than 8% the width of the cranial table (character 209, state 2); and, very large and widely spaced postoccipital processes of the supraoccipital (character 229, state 1).

## Discussion

The establishment of the novel taxon *Gunggamarandu maunala* has significant implications for crocodylian diversity and evolution in Australia, as well as globally. Australia’s Cenozoic crocodylian fauna was previously understood to have been dominated by mekosuchines, with the exception of the phylogenetically enigmatic *Harpacochampsa camfieldensis* during the middle Miocene, and species of *Crocodylus* appearing towards the end of the Neogene. The morphological and phylogenetic evaluation of *G. maunala* demonstrates that another clade, Tomistominae, was also present in Australia. Once a taxonomically diverse group, Tomistominae is today represented by only one species in one genus, *Tomistoma schlegelii*, found in Southeast Asia^63,64^. Throughout the Cenozoic, tomistomine crocodylians had a much wider distribution, with their fossils previously having been reported from all continents except Antarctica and Australia^46^ (note that Cidade et al.^65^ and De Celis et al.^66^ recommended a reinspection of the South American tomistomine record in order to confirm if such taxonomic identification is correct). *Gunggamarandu* is the first unambiguous representative of Tomistominae from Australia, with *G. maunala* marking the southern-most known record of the clade in the world. Also noteworthy is the size of the *G. maunala* holotype cranium – its size and proportions surpass those for the equivalent part of the skull of the largest *C. porosus* specimens known to us, and also outsize those of the largest known mekosuchines (i.e., *Baru* and *Paludirex*). Due to its incompleteness, providing a total length estimate for *G. maunala* based on QMF14.548 would be premature. Nevertheless, considering the proportions of QMF14.548 alone, it can be inferred that *G. maunala* is very likely the largest crocodyliform yet discovered in Australia.

### Phylogenetic implications

While our phylogenetic analyses consistently placed *Gunggamarandu* within Tomistominae (Fig. 4; see also Supplemental Document S2), and more specifically as a sister taxon to *Dollosuchoides*, we cannot yet be entirely certain of its precise position in the group. This is due to the preservational condition and incompleteness of the holotype (and thus far only) specimen, QMF14.548, which prevents scoring of many phylogenetic characters. However, if our results are correct, then the inferred basal position of *Gunggamarandu* within Tomistominae is extraordinary. Indeed, anatomical comparisons also indicate that *G. maunala* is morphologically most similar to basal tomistomines, such as *D. densmorei*, and especially *K. spenceri*. This is astonishing, since the aforementioned taxa are known from the Eocene of Europe, whereas *G. maunala* is from the Pliocene or Pleistocene of Australia. Under the scenario that these inferences are correct, it would suggest that there is a ghost lineage extending back at least to the early Eocene (Ypresian Age; 56–47.8 Ma) that links the primitive European tomistomines with the Australian taxon. Discovery of more complete *Gunggamarandu* specimens will provide answers to these tantalizing questions.

Another peculiarity of the phylogenetic results concerns *Harpacochampsa camfieldensis*, a Miocene crocodylian with enigmatic phylogenetic affinities. The phylogenetic placement of *H. camfieldensis* has been contentious^5,67^, with some studies recovering it as a possible mekosuchine^3,44,45,68–73^ while others in various positions within Crocodyloidea^2,7,23,74^. A couple of recent studies^6,24^ proposed gavialoid affinities for *H. camfieldensis*, and some of the analyses by Lee and Yates^24^ recovered topologies where *H. camfieldensis* clustered with tomistomines. If those results by Lee and Yates^24^ are correct, then it would imply that *H. camfieldensis* is the earliest known tomistomine from Australia. However, due to the persisting contradictory phylogenetic placements for *Harpacochampsa*, its exact phylogenetic affinity remains unclear. Thus, *Gunggamarandu* is presently the only confirmed tomistomine from the continent. Based on our results (Fig. 4; also, Ristevski et al.^7,74^) *Harpacochampsa* is neither a mekosuchine nor a tomistomine, and seems to be a representative of a different crocodylian lineage that inhabited Australia at some point in the past 66 Ma. Members of Mekosuchinae are still the most common crocodylians from Australia’s Cenozoic. However, in light of the results in this study it can be concluded that the higher-clade taxonomic diversity in Australia during most of the Cenozoic was greater than previously assumed.

### *Gunggamarandu* in context of Cenozoic Australian crocodylians

*Gunggamarandu* is morphologically distinct from all other known Australian crocodylians, and thus not referable to Mekosuchinae nor is it closely related to *Crocodylus* (Fig. 4). No known mekosuchines or species of *Crocodylus* possess the striking cranial features of *Gunggamarandu*, such as the very large supratemporal fenestrae, deeply concave cranial table, distinctive outline of the foramen magnum, and unusual morphology of the supraoccipital. Among the named Australian crocodylians, *G. maunala* is most similar to *H. camfieldensis*, with the greatest resemblance being the relative size of their supratemporal fenestrae. Even then, this feature is not identical between them, as *G. maunala* has proportionally larger supratemporal fenestrae. In virtually all other morphological features *G. maunala* clearly differs from *H. camfieldensis*, as the latter has a flat cranial table, a foramen magnum with an elliptical outline, and the supraoccipital of *H. camfieldensis* bears small postoccipital processes and a relatively flat occipital surface with a well-developed nuchal crest.

Based on the proportionately large supratemporal fenestrae and phylogenetic position within Tomistominae, it seems likely that *G. maunala* would have had a long, slender snout. In Crocodylia, a long and slender snout is not always accompanied by highly enlarged supratemporal fenestrae^75,76^. For example, even though *C. johnstoni* is a slender longirostrine species of *Crocodylus*, its supratemporal fenestrae are not significantly larger than species of *Crocodylus* with proportionately shorter and broader snouts (and while mature *C. johnstoni* have proportionally larger supratemporal fenestrae than mature *C. porosus*, this size difference is not drastic). However, highly enlarged supratemporal fenestrae that occupy much of the cranial table surface always correlate with a long and slender snout in known Crocodylia^31,49,77–81^. This is manifested in gavialids, ‘thoracosaurs’ and many tomistomines^43–47,53,61,62,82–100^. Whether *G. maunala* possessed a long and slender snout can only be proven conclusively once a more complete skull than the holotype is discovered. Nevertheless, the morphological and phylogenetic signals strongly point to such a snout morphology for *Gunggamarandu*. No known tomistomine deviates from the long and slender snout form (although the expression of snout elongation and constriction is variable between tomistomines; for example^45,47^, *M. zennaroi* is not as slender-snouted as *D. densmorei*), and since *G. maunala* is firmly nestled within Tomistominae, predicting a snout morphology in line with that of its sister taxa is the most parsimonious interpretation.

If the above-elaborated hypothesis holds true, then *G. maunala* would be the first slender longirostrine crocodylian recognized from the Darling Downs. Alongside *Gunggamarandu*, there are two accepted crocodylian genera from the Pliocene and/or Pleistocene of south-eastern Queensland—the broad-snouted non-ziphodont *Paludirex*, and the altirostral (tall-snouted) ziphodont *Quinkana*^7^. Different snout and dental morphologies enable crocodyliforms to employ different methods of prey acquisition and feeding strategies, and even have different prey type preferences^60,101–103^. Considering the disparity in the snout, dental and cranial morphologies exhibited between the crocodylian taxa from the Darling Downs (the former two only hypothesized for *G. maunala*) it is reasonable to assume that a certain degree of niche-partitioning would have allowed these crocodylians to co-exist in some form of sympatry. Afterall, sympatry between similarly sized crocodylians that have different cranial and snout morphologies is not without precedent today. In India, the slender longirostrine *Gavialis gangeticus* is sometimes sympatric with the broad-snouted *Crocodylus palustris*, as well as *C. porosus*^104^. In parts of Southeast Asia, the slender longirostrine *T. schlegelii* is sympatric with the broad-snouted *C. siamensis*, as well as *C. porosus*^105–107^. The fossil record also demonstrates multiple instances of sympatric crocodylian taxa with disparate craniodental morphologies^108,109^. Currently, it is uncertain if *G. maunala* shared the same environment with *Paludirex* or *Quinkana*—however, if these crocodylians did live in sympatry, then the Darling Downs would be another example of this trend.

### Arrival of tomistomines in Australia

The discovery of *G. maunala* poses the question of when tomistomines arrived in Australia. The earliest known tomistomines come from Europe and northern Africa, with the clade considered to have early Eocene or late Paleocene origins in the western Tethys^45^. Today, the only extant tomistomine is restricted to freshwater habitats in Southeast Asia^104^. However, many tomistomine fossils are known from estuarine and/or coastal deposits, indicating that at least some early tomistomines were capable of traversing marine environments, which allowed species within the clade to achieve a worldwide distribution during the Cenozoic^5,44–46,80,83,110–112^. The discovery of a tomistomine in Australia is not unexpected given the cosmopolitan status of Tomistominae throughout the Cenozoic. However, determining when this dispersal first occurred and via which route is more difficult to ascertain.

Assuming that the most likely point of origin for the common ancestor of *Gunggamarandu* and other early diverging tomistomines was the western Tethys, the most plausible palaeobiogeographic scenario for their dispersal to Australia is via Southeast Asia through the Sundaic region and Wallacea. Another, but probably less likely possibility, is an arrival into Australia directly from the west via Africa. Depending on when it might have occurred, this scenario would imply a substantial marine trek across the Indian Ocean. A third potential route would be directly from the Americas to the west. This scenario is perhaps the least likely as it would have required crossing the vast expanse of the Pacific Ocean.

Since there is currently only one definitive tomistomine specimen from Australia’s fossil record—the *G. maunala* holotype, QMF14.548—it is unknown if this clade was present on the continent prior to the Pliocene (or Pleistocene). If the smallest crossing distance is the main point of consideration, then dispersal from Southeast Asia to Australia seems to be the most plausible. By the late Oligocene (Chattian; ~ 25.7 Ma), Australia and small land areas of New Guinea were roughly equidistant from Antarctica to the south and the Sundaic region and the complex island arcs of Wallacea to the north^113–116^. By the end of the Oligocene (c. 25 Ma) and early Miocene (c. 20 Ma), the northern margin of Sahul (Australia + New Guinea) would have been sufficiently close to emergent parts of Wallacea along the East Philippines-Halmahera-South Caroline arc to have facilitated the southern dispersal of tomistomines (see Figs. 20 and 21 in Hall^113^). By the middle Miocene (Langhian–Serravallian; 15.97–11.63 Ma), the distance between the northwestern margin of Australia and the southern margin of Southeast Asia began to approach its modern span, and dispersal for crocodylians with the capacity to cross relatively short marine barriers through the Sundaic region would have become much easier. Several other reptile groups that inhabit Australia have origins from Southeast Asia, and could have been present in Australia by the late Paleogene^117^. Based on these observations, it is plausible that tomistomines arrived in Australia from Southeast Asia from the middle Miocene onwards. However, a pre-Miocene arrival for tomistomines cannot be ruled out, considering the potential Eocene divergence date between *Gunggamarandu* and other basal European tomistomines.

*Gunggamarandu* is not the oldest non-mekosuchine from Australia’s Cenozoic. Considering that Australia became sufficiently close to Southeast Asia during the middle Miocene, it may not be coincidental that the first non-mekosuchine—*Harpacochampsa camfieldensis*—appeared during this time. The next recorded non-mekosuchine crocodylians are known from the Pliocene onwards (i.e., *Crocodylus* and *Gunggamarandu*). The fossil record is yet to reveal a trail through Southeast Asia (or through Africa or the Americas, for that matter) for tomistomines that have strong similarities to basal European taxa like *Gunggamarandu* does. The recognition of *Gunggamarandu* adds more questions on the dispersal and relationships of tomistomines, with additional, and more complete, material being needed in order to unravel the mysteries of this group.

## Methods

### Computed tomographic scanning and 3D digital models

Using CT scanning, we were able to digitally segment several endocranial structures of QMF14.548, including the brain endocast, trigeminal nerve canals, endosseous labyrinths, and intertympanic diverticulum. The endocranial cavities of QMF14.548 are filled with matrix, which combined with the relatively low resolution of the scan prevented discerning of most other, rather delicate, elements of the endocranium (such as the remaining cranial nerve canals, vascular canals, and components of the paratympanic sinus system). Nonetheless, this makes the present contribution the most comprehensive palaeoneurological assessment of an extinct tomistomine yet. Prior to this study, *Maomingosuchus petrolica* was the only tomistomine with partially known endocranial elements, however, these are based on a ‘natural endocast’ for which a limited amount of information is currently available (see Yeh^118^).

The images acquired from the CT scan are DICOM files that were imported into the specialized 3D image processing software Mimics 22.0 (Materialise NV, Belgium) at The University of Queensland. A digital model of QMF14.548 was generated in Mimics 22.0, where the endocranial features were manually (and seldomly semi-automatically, by having the Auto interpolate option activated) segmented using several tools from the SEGMENT menu. Afterwards, the digital models of the cranium and segmented endocranial features were exported as STL (stereolithography) files. The STL files of the segmented endocranial structures were then imported into Materialise 3-matic 14.0 in order to create the interactive 3D PDF that is added as a supplement to this paper. Details on the CT scanning settings and parameters are given in Supplemental Document S3 to this paper.

### Mensuration

The external linear measurements of QMF14.548 were taken directly using a hand-held 150 mm digital caliper. Where the dimensions exceeded 150 mm, a 5 m measuring tape was used instead. The digitally segmented endocranial structures were measured in Mimics 22.0 using the Distance tool from the MEASURE menu.

### Phylogenetic methods

The phylogenetic assessments undertaken in this study are based on a slightly updated and expanded version of the matrix by Ristevski et al.^7,74^ This version of the matrix has 229 morphological characters and 142 operational taxonomic units (OTUs). Out of the 229 morphological characters, *Gunggamarandu maunala* could be scored for 25 (i.e., 10.92%) characters in total. Serving as an outgroup taxon in the matrix was the goniopholidid crocodyliform *Anteophthalmosuchus epikrator*. Four separate analyses were performed, one under a ‘traditional’ equal weighting (EW) principal search methodology, and three analyses that used the implied weighting (IW) methodology^119^. In the three IW analyses, the *k* (concavity constant) values were set to 3.0 (k = 3), 12.0 (k = 12), and 25.0 (k = 25). In all analyses, 19 out of the 229 characters were treated as ordered (characters 21, 39, 49, 50, 54, 55, 82, 83, 89, 118, 125, 137, 142, 150, 160, 176, 202, 223 and 224). The phylogenetic analyses were carried out in TNT v1.5 Willi Hennig Society Edition^120,121^. The same search protocols as in Ristevski et al.^7,74^ were used here as well. The program was set to 900 Mb of RAM, with the maximum number of held trees being 99, 999. The parameters applied in the analyses follow Young et al.^122^, which implement the new technology searches (sectorial search, ratchet, drift, and tree fusion) set to 1000 random addition sequences (RAS). For the sectorial search, the selection size above 75 used 1000 drifting cycles, 1000 starts below 75 and trees were fused 1000 times. In addition, the consensus sectorial search (CSS) and exclusive sectorial search (XSS) were set to 1000 rounds. For ratchet, the parameters were set to stop the perturbation phase when 1000 substitutions were made, or 99% of the swapping was completed and a total of 1000 iterations. For drift, the perturbation phase stopped when 1000 substitutions were made, or 99% of the swapping was completed, and the number of cycles was set to 1000. No changes were made to the tree fusion settings which were left at the default three rounds. Nodal support was assessed by conducting Bremer support and bootstrap analyses. The Bremer support was performed by running the script ‘BREMER.RUN’, which is provided with the TNT v1.5 download package, and used the default settings. The bootstrap analysis^123,124^ was set to 1000 replicates, showing values of 50% and above. Two homoplasy metrics, the consistency index (CI; Kluge and Farris^125^) and retention index (RI; Farris^126^), were calculated by running the script ‘STATS.RUN’, also provided in the TNT v1.5 download package. Additional information on the taxon matrix and character dataset are given in Supplemental Document S2 to this paper (see also Ristevski et al.^74^), with the raw data of the matrix (in NEXUS format) and results (in native .tnt formats) also provided as supplements.

### Nomenclatural acts

This published work and the nomenclatural acts it contains have been registered in ZooBank, the proposed online registration system for the International Code of Zoological Nomenclature. The ZooBank Life Science Identifiers (LSIDs) can be resolved and the associated information viewed by appending the LSIDs to the prefix http://zoobank.org/. The LSID for this publication is [urn:lsid:zoobank.org:pub:FF79BD61-D67C-4F19-9F4A-8FF57FD16FF9]. The LSID for the genus *Gunggamarandu* is [urn:lsid:zoobank.org:act:2272097D-3DC7-4550-9F18-480C27E1EDBB], and that for the species *Gunggamarandu maunala* is [urn:lsid:zoobank.org:act:A4BA4F02-0510-4AFF-9BD5-6D34A30786ED]. Raw CT data The raw CT data for QMF14.548 is available at MorphoSource: https://www.morphosource.org/concern/parent/000344708/media/000344711

### Ethics declarations

The authors confirm that all research methods and study aspects were carried out in accordance with relevant guidelines and regulations. This study focuses on describing a fossil specimen that is housed at the Queensland Museum in Brisbane, Australia. No live animals were involved at any point of the study.

## Supplementary Information

Supplementary Information 1.

Supplementary Information 2.

Supplementary Information 3.

Supplementary Information 4.

Supplementary Information 5.

Supplementary Information 6.

Supplementary Information 7.

Supplementary Information 8.

Supplementary Information 9.
