# Supplementary material for: First record of a tomistomine crocodylian from Australia
Source: Sci Rep. 2021 Jun 9;11:12158. doi: 10.1038/s41598-021-91717-y (PMC8190066; doi:10.1038/s41598-021-91717-y)
Supplement: Supplementary file 1 — Supplementary Information 1. [file 41598_2021_91717_MOESM1_ESM.pdf]

SUPPLEMENTARY MATERIAL FOR

FIRST RECORD OF A TOMISTOMINE CROCODYLIAN FROM AUSTRALIA

SUPPLEMENTAL DOCUMENT S1: EXPANDED DESCRIPTION OF QMF14.548

by JORGO RISTEVSKI[1*], GILBERT J. PRICE[2], VERA WEISBECKER[1, 3] and STEVEN W. SALISBURY[1]

[1]School of Biological Sciences, The University of Queensland, Brisbane, 4072, Queensland, Australia

[2]School of Earth and Environmental Sciences, The University of Queensland, Brisbane, 4072, Queensland, Australia

[3]College of Science and Engineering, Flinders University, Bedford Park 5042, South Australia, Australia

***Corresponding Author:**

Jorgo Ristevski[1]

School of Biological Sciences, Goddard Building (Building 8), The University of Queensland, Brisbane 4072, Queensland, Australia

Email address: j.ristevski@uq.net.au

TABLE OF CONTENTS

Description 2
- Major cranial fossae, fenestrae and foramina 2
  - Supratemporal fossae and fenestrae 2
  - Temporoorbital foramina 3
  - Posttemporal fenestrae 4
  - Foramen magnum 5
  - Otic aperture 5
- Dermatocranial bones 7
  - Frontal 7
  - Parietal 8
  - Pterygoids 10
- Chondrocranial bones 12
  - Laterosphenoids 12
  - Prootics 15
  - Supraoccipital 16
  - Otoccipitals 17
  - Basioccipital 18
  - Basisphenoid 19
- Splanchnocranial bones 19
  - Quadrates 19
- Endocranial morphology 20
  - Brain endocast 20
  - Trigeminal nerve canals, trigeminal foramina and fossae 24
  - Endosseous labyrinths 25

Institutional abbreviation 27

References 27

DESCRIPTION

In this supplementary document to the study titled "First record of a tomistomine crocodylian from Australia" is given a detailed description of the osteology (**Figs. S1.1–S1.8**) and endocranial morphology (**Figs. S1.9–S1.12**) of the *Gunggamarandu maunala* holotype specimen, QMF14.548. Readers are encouraged to use the interactive 3D PDF of QMF14.548 (provided as a supplement to the paper) in tandem with the following description.

Major cranial fossae, fenestrae and foramina

Supratemporal fossae and fenestrae

The supratemporal fossae and fenestrae (=dorsotemporal or upper temporal fossae and fenestrae) are only missing their lateral margins, but what is preserved of them clearly reveals that they are very large and occupy a significant portion of the cranial table (**Figs. S1.1**, **S1.5**, **S1.7**, **S1.8B–S1.8E** and **S1.9A**). Each fenestra has an anteroposterior length of ~86 mm and a preserved transverse width of ~70 mm (because the supratemporal arches are missing, the maximum transverse width of each fenestra exceeded 70 mm). The supratemporal fossae are most expansive at their medial and posterior allocations. The frontoparietal fossae (*sensu* Holliday *et al.*, 2020) are not particularly prominent, being slightly apparent in dorsal view anteriorly near the sutures for the postorbitals, yet they are not exposed anteromedially over the preserved dorsal cranial surfaces (character 210, state 0; **Fig. S1.1**). The parietal forms the anteromedial, medial and posteromedial margins of the supratemporal fenestrae. The medial and posteromedial margins of both fenestrae are very slightly overhung by the lateral and posterolateral margins of the parietal's dorsal lamina. The left supratemporal fenestra is additionally overhung anteromedially by the anterolateral margin of the parietal's dorsal lamina; this overhang is not present on the right side. Regardless, the overhanging of the fenestrae's margins is negligible (character 152, state 0).

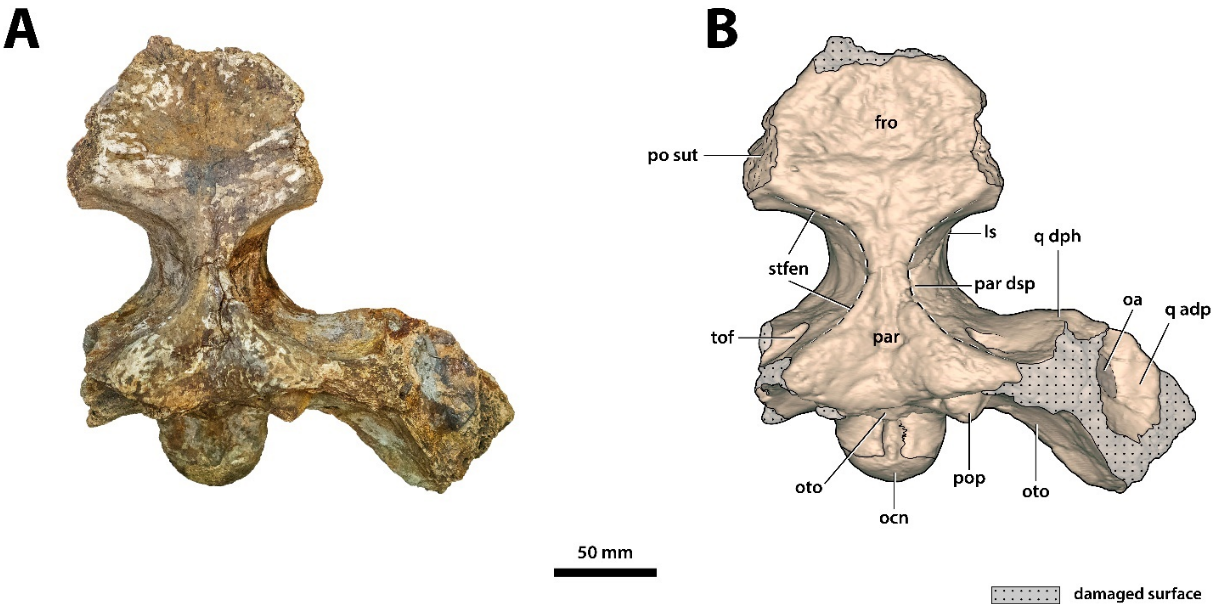

Figure S1.1 ***Gunggamarandu maunala* gen. et sp. nov., QMF14.548, holotype, cranium in dorsal view.** (A) Photograph, and (B) annotated digital model. Abbreviations: **fro**, frontal; **ls**, laterosphenoid; **oa**, otic aperture; **ocn**, occipital condyle; **oto**, otoccipital; **par**, parietal; **par dsp**, parietal descending process; **po sut**, sutural surface for articulation with the postorbital; **pop**, postoccipital process of the supraoccipital; **q adp**, quadrate anterodorsal process; **q dph**, quadrate dorsal primary head; **stfen**, supratemporal fenestra; **tof**, temporoorbital foramen.

Temporoorbital foramina

The temporoorbital foramina (=temporal canals or orbitotemporal passages; **Figs. S1.1** and **S1.5**) are evident as two transversely elongated depressions on the posterior walls of the supratemporal fossae. Their overall contours are well delineated, but the foramina are completely closed due to fossilization. These foramina are set at a small distance ventral to the dorsal rims of the supratemporal fenestrae (~17 mm from the posterior margins of the supratemporal fenestrae at the postfenestral bars) and oriented anterolaterally. The bony surface anteroventral to each foramen is slightly expanded into a shallow fossa. The temporoorbital foramina on the posterior walls of the supratemporal fossae are more ventrally positioned than the posttemporal fenestra(e) on the occiput. Both the left and right temporoorbital foramen has a transverse length of ~31–32

mm and dorsoventral height of ~12–14 mm. The sutures of the bones surrounding these foramina are not visible.

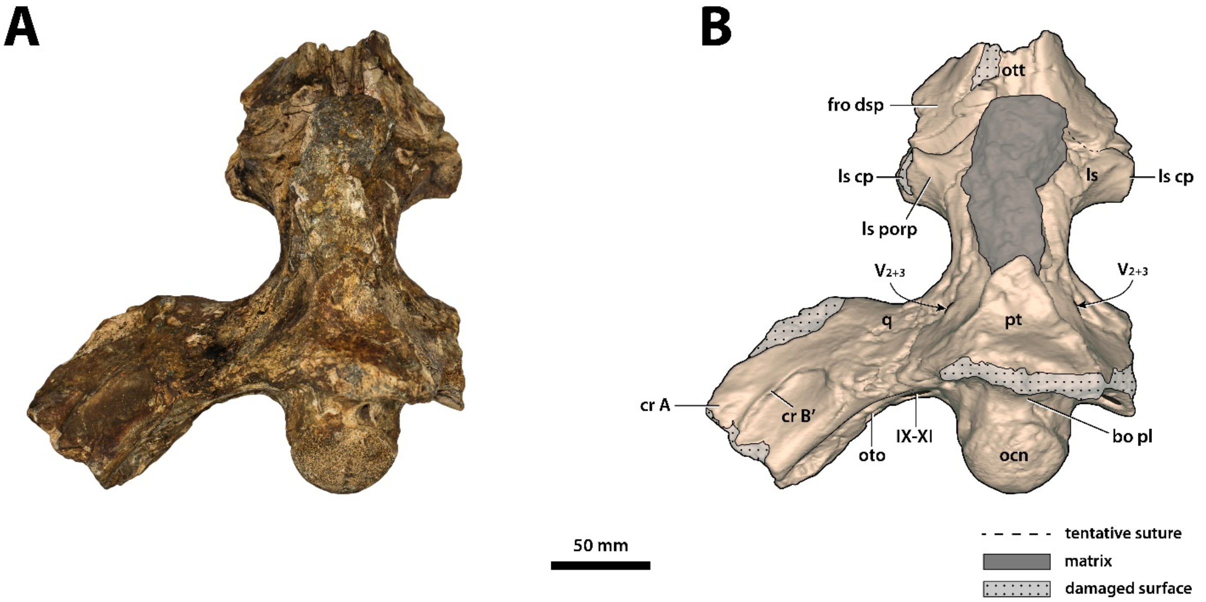

Figure S1.2 ***Gunggamarandu maunala*** **gen. et sp. nov., QMF14.548, holotype, cranium in ventral view.** (A) Photograph, and (B) annotated digital model. Abbreviations: **bo pl**, basioccipital plate; **cr A**, crest A of quadrate; **cr B′**, crest B′ of quadrate; **fro dsp**, frontal descending process; **IX-XI**, foramen for glossopharyngeal, vagus, and accessory nerves (metotic foramen); **ls**, laterosphenoid; **ls cp**, laterosphenoid capitate process; **ls porp**, laterosphenoid postorbital process; **ocn**, occipital condyle; **oto**, otoccipital; **ott**, olfactory tract trough; **pt**, pterygoid; **q**, quadrate; $\mathbf{V_{2+3}}$, maxillomandibular foramen.

Posttemporal fenestrae

On the occiput, the more clearly visible posttemporal fenestra is the one on the right side (**Fig. S1.4**). The fenestra is narrow and crescent-like, however, its insides are filled with matrix. It has a transverse length of ~32 mm, while the dorsomedial height is significantly less, ~2 mm. Ventrally, the fenestra is bound by the large posteriorly directed postoccipital process of the supraoccipital (*sensu* Kälin, 1933) and dorsally by the parietal.

Foramen magnum

Found centrally on the occiput is the large foramen magnum (**Fig. S1.4**). Its margins are complete and well defined. The foramen is distinctly obround, with a straight dorsal, minimally concave ventral, and semicircular lateral margins. Its transverse width is twice its dorsoventral height (~44 mm and ~22 mm respectively), yet the foramen's dimensions are smaller than those of the occipital condyle (see "Basioccipital" subsection below). Except for its ventromedial margin which is formed by the basioccipital, the foramen magnum is otherwise entirely surrounded by the otoccipitals. Furthermore, the dorsal margin of the foramen magnum is made of a short, posteriorly projecting shelf formed by the medial contact of the otoccipitals.

Otic aperture

The meatal chamber region (*sensu* Montefeltro *et al*., 2016) is partially preserved only on the right side (**Figs. S1.1**, **S1.3** and **S1.6**), with the conspicuous otic aperture having a shape in the form of the letter D. The otic aperture is entirely filled with matrix. Following the terminology of Montefeltro *et al.* (2016), the semilunar otic incisure delimits the anterior, anterodorsal and anteroventral margins of the aperture, whereas the posteroventral margin is delimited by the incisure of the otic aperture of the cranioquadrate passage which is bounded by the anterodorsal process of the quadrate. The posterodorsal margin of the otic aperture is delimited by the dorsal otic incisure, which is bounded by the smooth and gently concave anteromedial margin of the squamosal's posterior descending lamina (=posteroventral prong of the squamosal *sensu* Montefeltro *et al*., 2016). The dimensions of the otic aperture are ~27 mm in anteroposterior length (measured midway from the posterior to anterior margins) and ~29 mm in dorsoventral height (measured from the incisure of the otic aperture of the cranioquadrate passage ventrally to the dorsal otic incisure dorsally). The short and anteroposteriorly directed quadratosquamosal suture

can be detected on the posterior margin of the aperture, just dorsal to the incisure of the otic aperture of the cranioquadrate passage (character 148, state 1).

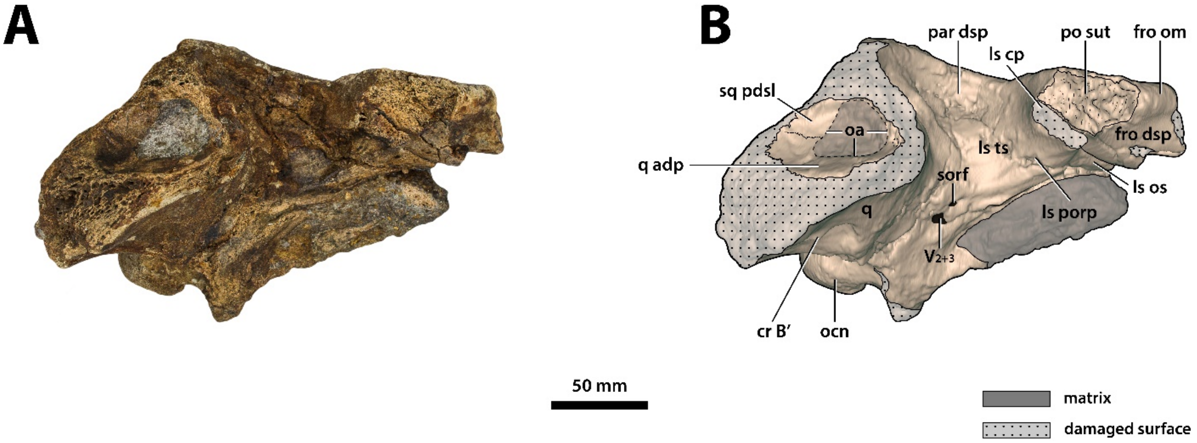

Figure S1.3 ***Gunggamarandu maunala* gen. et sp. nov., QMF14.548, holotype, cranium in right lateral view.** (A) Photogram, and (B) annotated digital model. Abbreviations: **cr B′**, crest B′ of quadrate; **fro dsp**, frontal descending process; **fro om**, frontal orbital margin; **ls cp**, laterosphenoid capitate process; **ls os**, laterosphenoid orbital surface; **ls porp**, laterosphenoid postorbital process; **ls ts**, laterosphenoid temporal surface; **oa**, otic aperture; **ocn**, occipital condyle; **par dsp**, parietal descending process; **po sut**, sutural surface for articulation with the postorbital; **q**, quadrate; **q adp**, quadrate anterodorsal process; **sq pdsl**, squamosal posterior descending lamina; **sorf**, supraorbital foramen; **V_{2+3}**, maxillomandibular foramen.

Lateral to the aperture is the wide and smooth surface of the anterodorsal process of the quadrate. The portion of the quadrate's anterodorsal process adjacent to the otic aperture is largely preserved, although missing the bulk of its anterior and anterodorsal portions. The periotic fossa is determinable anteromedially on the anterodorsal process, although it is relatively shallow and weakly defined. Anterior to the otic aperture and lying on the periotic fossa is the small and sub-circular subtympanic foramen (=preotic siphoneal foramen of Kley *et al.*, 2010). The foramen is filled with matrix, and due to the anterior breakage on the quadrate its anterior-most margin is missing. The subtympanic foramen has a relatively small diameter (~3 mm). The otic buttress is not exposed in QMF14.548, as it is likely covered by matrix.

Dermatocranial bones

Frontal

The frontal (**Figs. S1.1–S1.3** and **S1.7–S1.9**) is relatively well preserved and mostly complete, missing only its anterior process. Posteriorly, the frontal is in firm sutural contact with the parietal. The frontoparietal suture is not visible externally on the specimen, however, it is discernable in the CT scans (**Figs. S1.8B–S1.8E**). This sutural contact occurs anterior to the supratemporal fenestrae, such that the frontal has no contribution to the fenestrae's margins (character 150, state 2). Although the postorbitals are missing, the sutural surfaces for articulation with the anteromedial postorbital processes are discernable at the lateral margins of the frontal and parietal. Additionally, small lengths of the posteromedial orbital margins are also preserved anterolaterally on the frontal, just anterior to the aforementioned sutural surfaces for contact with the postorbitals.

The frontal's dorsal lamina (=dorsal surface) is quite broad and has an approximately trapezoidal shape. As revealed by the anterior breakage, the bone is very thick (~27 mm dorsoventral thickness, measured at the anterior-most preserved portion). There is also a conspicuous medial concavity that is widely spread over the frontal's dorsal lamina. Such shallow and wide concavities on the frontal are common among mature specimens of certain basal tomistomines (e.g., *Dollosuchoides densmorei*, Brochu, 2007; *Gavialosuchus eggenburgensis* Toula & Kail, 1885; *Kentisuchus* Mook, 1955, see p. 171 in Jouve, 2016; *Maroccosuchus zennaroi* Jonet & Wouters, 1977, see p. 429 in Jouve *et al.*, 2015; *Megadontosuchus arduini* [de Zigno, 1880], see p. 318 in Piras *et al.*, 2007). Dorsally, the ornamentation is predominantly composed of grooves delimited by low ridges that are discernable at the anterior, lateral and posterior margins of the frontal's dorsal lamina. Medially on the element (amidst the medial concavity) the ornamentation is more difficult to see, however, it seems that the groove-like ornamentation is continuous medially as well. The descending processes of the frontal (=cristae cranii frontales) are smooth,

bear tiny foramina and ventrally they contact the orbital surfaces of the laterosphenoids. Only the posterior portions of the descending processes are present, cradling a shallow through that would have held the base of the olfactory tract. The width of the olfactory tract trough at its preserved posterior is ~21 mm. The descending process on the right side is largely damaged and not as well preserved as the left.

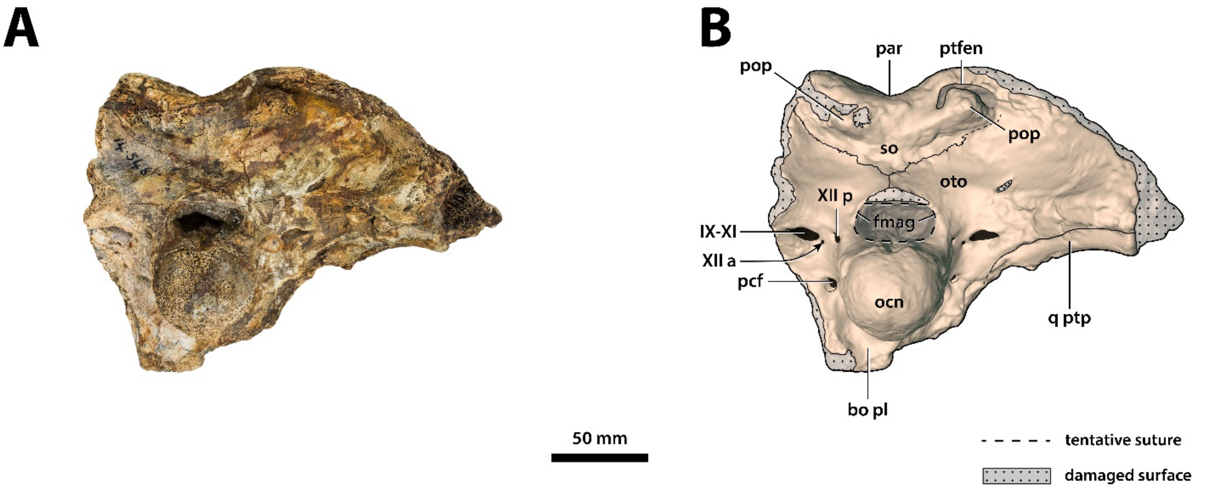

Figure S1.4 ***Gunggamarandu maunala*** **gen. et sp. nov., QMF14.548, holotype, cranium in occipital view.** (A) Photograph, and (B) annotated digital model. Abbreviations: **bo pl**, basioccipital plate; **fmag**, foramen magnum; **IX-XI**, foramen for glossopharyngeal, vagus, and accessory nerves (metotic foramen); **ocn**, occipital condyle; **oto**, otoccipital; **par**, parietal; **pcf**, posterior carotid foramen; **pop**, postoccipital process of the supraoccipital; **ptfen**, posttemporal fenestra; **q ptp**, quadrate pterygoid process; **so**, supraoccipital; **XII a**, anterior hypoglossal foramen; **XII p**, posterior hypoglossal foramen.

Parietal

The parietal (**Figs. S1.1**, **S1.3–S1.5** and **S1.7–S1.9**) is well preserved and occupies the posteromedial part of the cranial table. Aside from its contact with the frontal (mentioned above), the parietal's sutural contact with the quadrates and laterosphenoids is, unfortunately, undiscernible. Nevertheless, the parietal is still in contact with those elements. Anterolaterally, the parietal contacted the postorbitals and posterolaterally the squamosals. Posteroventrally, the

parietal contacts the supraoccipital, however, it is undetermined if the supraoccipital had a dorsal exposure on the cranial table.

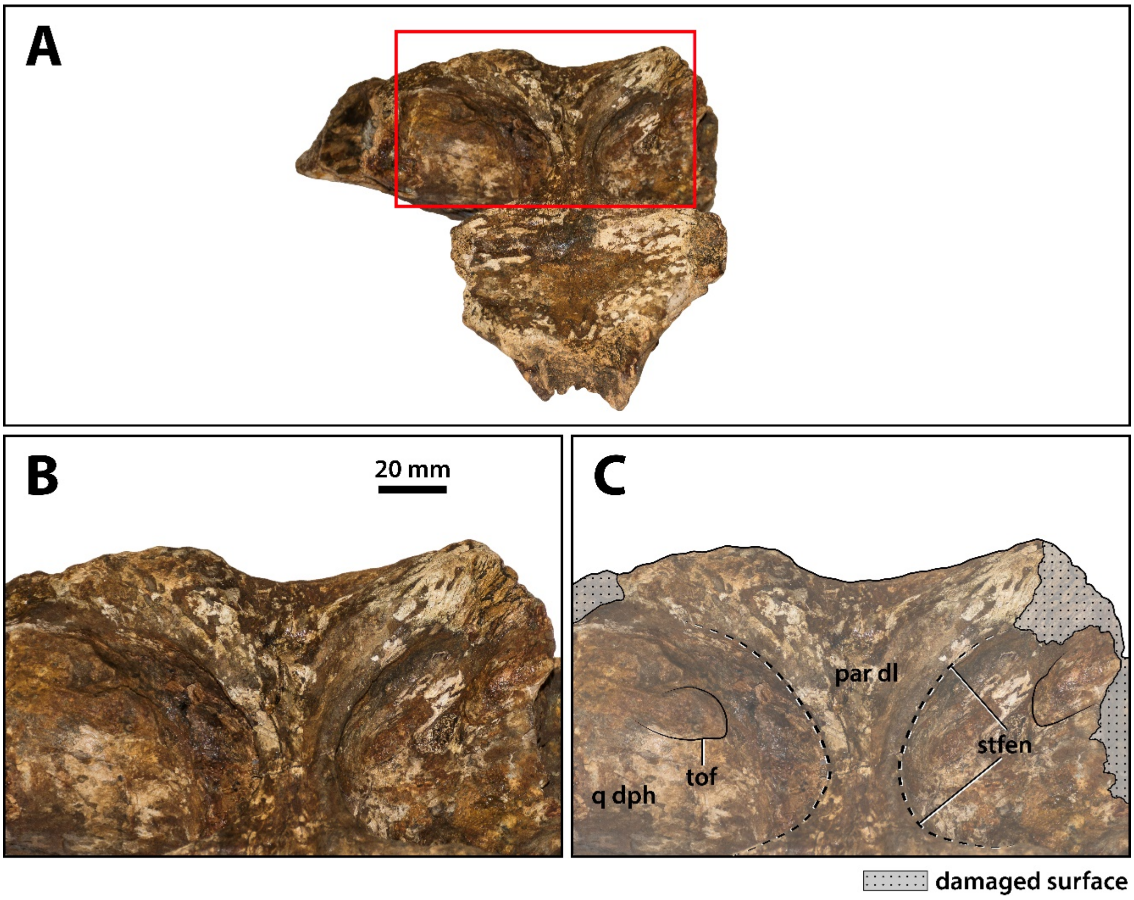

Figure S1.5 ***Gunggamarandu maunala*** **gen. et sp. nov., QMF14.548, holotype, close up of the supratemporal fenestrae and temporoorbital foramina.** (A) Cranium in anterodorsal view, highlighting the area shown (B) as a non-annotated photograph, and (C) annotated photograph. Abbreviations: **par dl**, parietal dorsal lamina; **q dph**, quadrate dorsal primary head; **stfen**, supratemporal fenestra; **tof**, temporoorbital foramen.

The parietal is a large element that comprises a significant portion of the cranium, yet due to the large supratemporal fenestrae, the parietal's dorsal lamina has a proportionately narrow surface area. The interfenestral bar is highly constricted between the supratemporal fenestrae, having a width of ~19 mm (character 208, state 0). Likewise, the postfenestral bars are also proportionately very narrow (~18 mm as measured on the more complete right bar at the place

where the contact between the parietal and medial squamosal process would have occurred; character 209, state 2). The lateral margins of the interfenestral bar (or, the medial margins of the supratemporal fenestrae) are slightly upturned. Anterior to the supratemporal fenestrae, the ornamentation on the dorsal lamina of the parietal is somewhat difficult to ascertain although it appears to be made of grooves and low ridges, like on the frontal. The ornamentation on the interfenestral bar is not as intense as on the other portions of the parietal's dorsal lamina. The ornamentation on the parietal at the posteromedial margins of the supratemporal fenestrae is composed of long and narrow grooves and irregularly shaped pits, while more medially on the element the details are obscure due to the preservational state on the surface. The most peculiar feature of the parietal is found at its posterior portion, which is the deep and sub-triangular medial concavity. Such a sub-triangular concavity has previously been reported in *Kentisuchus spenceri* (Buckland, 1836), a basal tomistomine from the early Eocene of England (see Brochu, 2007). The posterolateral portions of the parietal's dorsal lamina not affected by the sub-triangular concavity are gently swelled up. As a consequence of the sub-triangular concavity, the medial portion of the cranial table attains a cradle-like appearance when observed in occipital view (**Fig. S1.4**), and gently slopes posteroventrally. The ventrally directed descending processes of the parietal (=cristae cranii parietales) are visible through the supratemporal fenestrae when observed dorsally and have smooth and concave surfaces. Both left and right descending process is affected by few cracks. Internally, no parietal diverticulum is present (character 167, state 1; **Figs. S1.9B–S1.9D**).

Pterygoids

Only small portions of each ascending process of the pterygoid (=basisphenoid process of pterygoid *sensu* Dufeau & Witmer, 2015) are preserved, that are almost non-descript. The left lateral side preserves a slightly more extensive part of the ascending process as opposed to the right. The preserved pterygoid surfaces are smooth. A conspicuous but shallow concavity on the left ascending process is present on the posteroventrolateral-most preserved portion of the specimen.

Anterodorsally, or near the anteroventral margin of the laterosphenoids, the pterygoids have slightly concave surfaces. Ventromedially on the specimen is a poorly preserved sub-triangular concave area that most likely corresponds to the dorsal wall of the secondary choanae formed by the medial contact of the pterygoids (**Fig. S1.2**). The sutures between the pterygoids and their neighboring elements are not distinguishable.

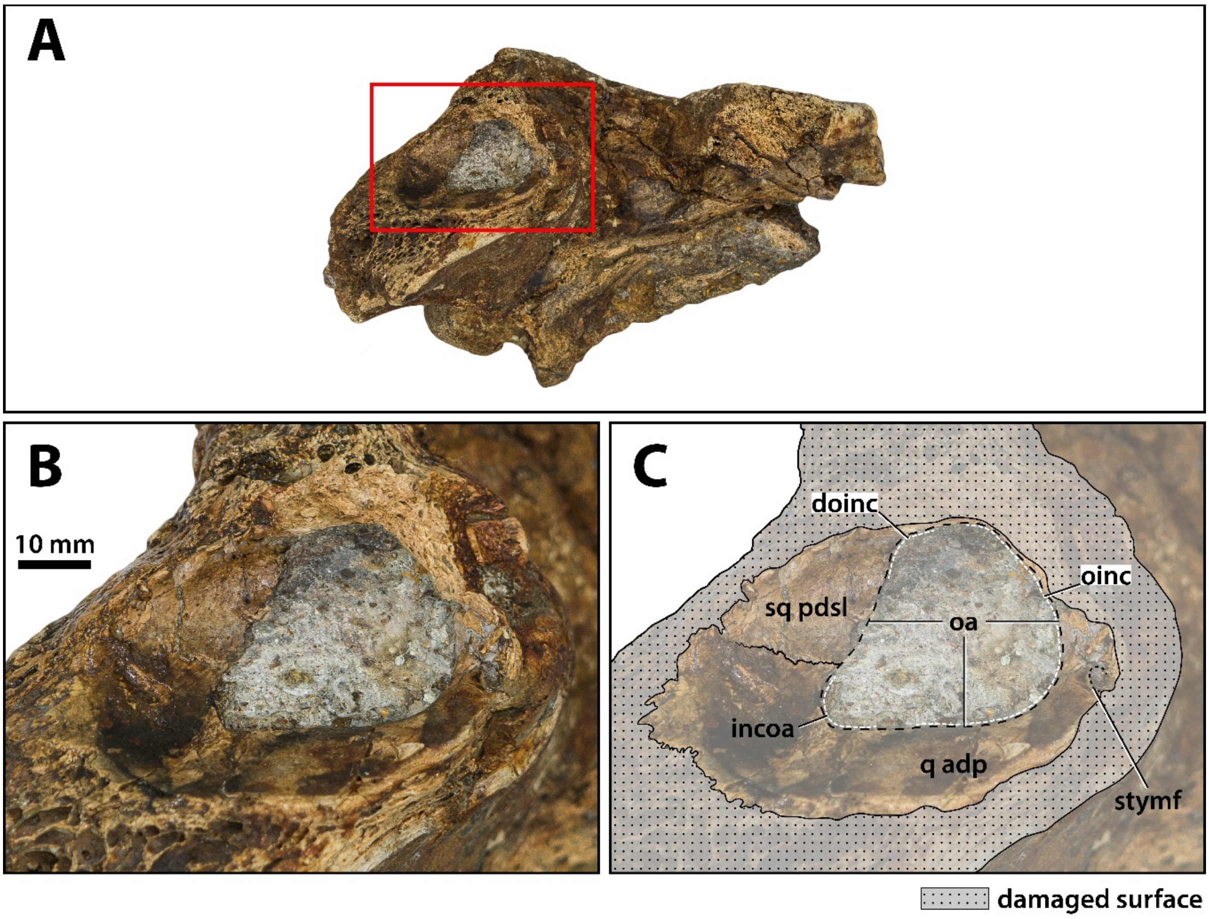

Figure S1.6 ***Gunggamarandu maunala*** **gen. et sp. nov., QMF14.548, holotype, right meatal chamber region.** (A) Cranium in right lateral view, highlighting the area shown (B) as a non-annotated photograph, and (C) annotated photograph. Abbreviations: **doinc**, dorsal otic incisure; **incoa**, incisure of the otic aperture of the cranioquadrate passage; **oa**, otic aperture; **oinc**, otic incisure; **q adp**, quadrate anterodorsal process; **sq pdsl**, squamosal posterior descending lamina; **stymf**, subtympanic foramen.

Chondrocranial bones

Laterosphenoids

Both laterosphenoids (**Figs. S1.1–S1.3** and **S1.7**) are mostly complete, but nonetheless heavily affected by cracks. The laterosphenoids are robust structures that form the anterolateral portions of the braincase. Both laterosphenoids are partially fractured, and the tips of their capitate processes are pinched-off. The external bone texture on the left laterosphenoid is better preserved than on the right. The capitate processes are oriented anteroposteriorly towards the midline so that the opening for the olfactory tract occurs more anterior relative to them (character 166, state 1). Additionally, the capitate processes are loosely in contact with the frontal and contacted the postorbitals as well. The suture between the laterosphenoid and frontal is most evident on the right side as sub-horizontal and straight, terminating medially ventral to the descending process of the frontal at the lateral margin of the exit for the olfactory tract foramen (**Fig. S1.7**). Stretching anteroventrally on the postorbital process of each laterosphenoid (*sensu* Holliday & Witmer, 2009) and continuing posteroventrally is the blunt cotylar crest (Busbey, 1989; Holliday & Witmer, 2009). On the right side, the cotylar crest appears to terminate slightly anterodorsal to the supraorbital foramen groove. Anteroventral to the cotylar crest is the sharp tensor crest. The tensor crest is stretched anteroposteriorly and is slightly dorsoventrally inclined. Posteriorly, the tensor crest terminates anterior to the trigeminal fossa. The laterosphenoid bodies (*sensu* Holliday & Witmer, 2009) bound the anterior margins of the trigeminal foramina. Unfortunately, details and structures, such as the lateral bridge and cavum epiptericum surrounding the trigeminal foramen are not distinguishable on the surface, excepting for the caudal laterosphenoid bridge (*sensu* Holliday & Witmer, 2009) that is partially preserved as a short lamina over the dorsal margin of the trigeminal foramen, separating the supraorbital from trigeminal foramen on the right side (**Fig. S1.7**). Anteromedially, the laterosphenoids are covered by a thick mass of matrix.

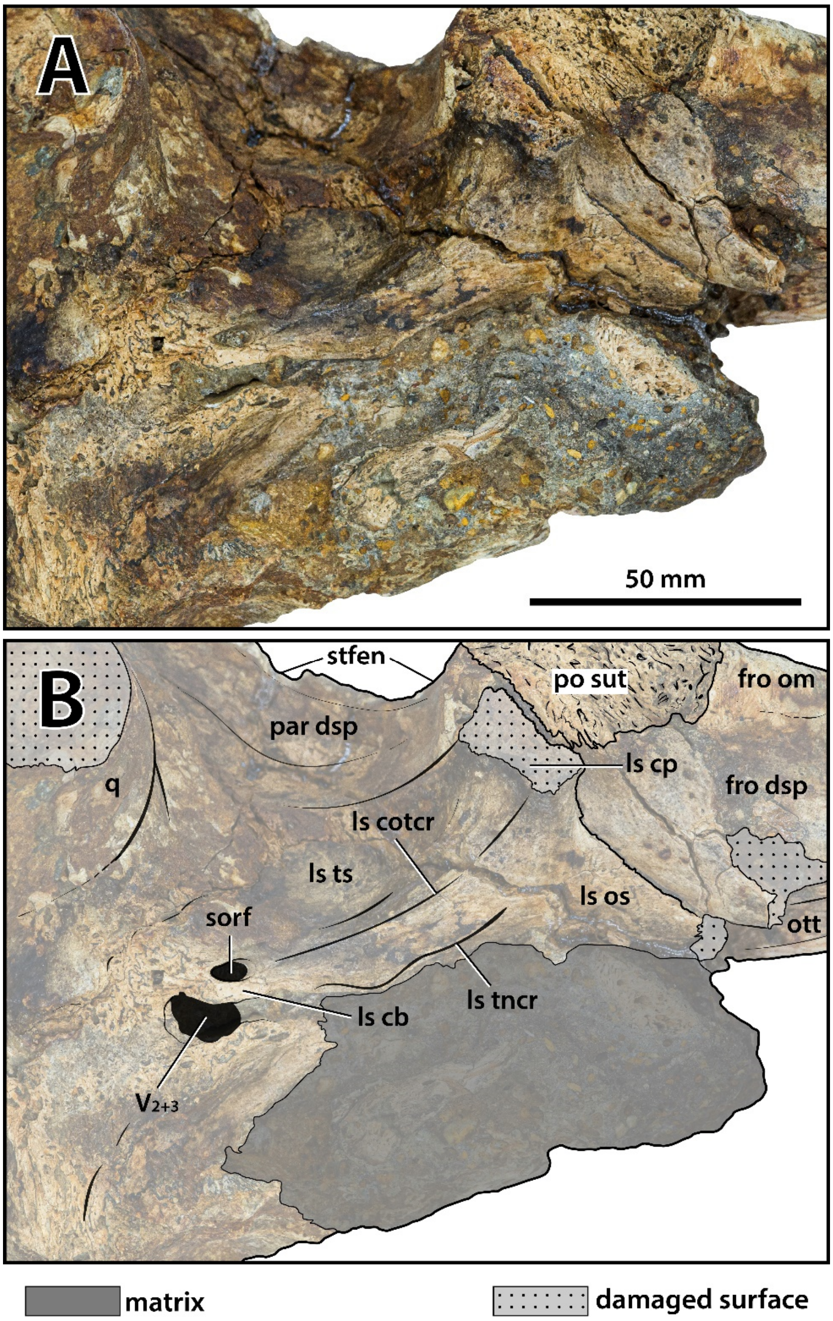

Figure S1.7 ***Gunggamarandu maunala* gen. et sp. nov., QMF14.548, holotype, close up of the right orbitotemporal region in oblique lateral view.** (A) Non-annotated photograph, and (B) annotated photograph. Abbreviations: **fro dsp**, frontal descending process; **fro om**, frontal orbital margin; **ls cb**, laterosphenoid caudal bridge; **ls cotcr**, laterosphenoid cotylar crest; **ls cp**, laterosphenoid capitate process; **ls os**, laterosphenoid orbital surface; **ls tncr**, laterosphenoid tensor crest; **ls ts**, laterosphenoid temporal surface; **ott**, olfactory tract trough; **par dsp**, parietal descending process; **po sut**, sutural surface for articulation with the postorbital; **q**, quadrate; **sorf**, supraorbital foramen; **stfen**, supratemporal fenestra; **V_{2+3}**, maxillomandibular foramen.

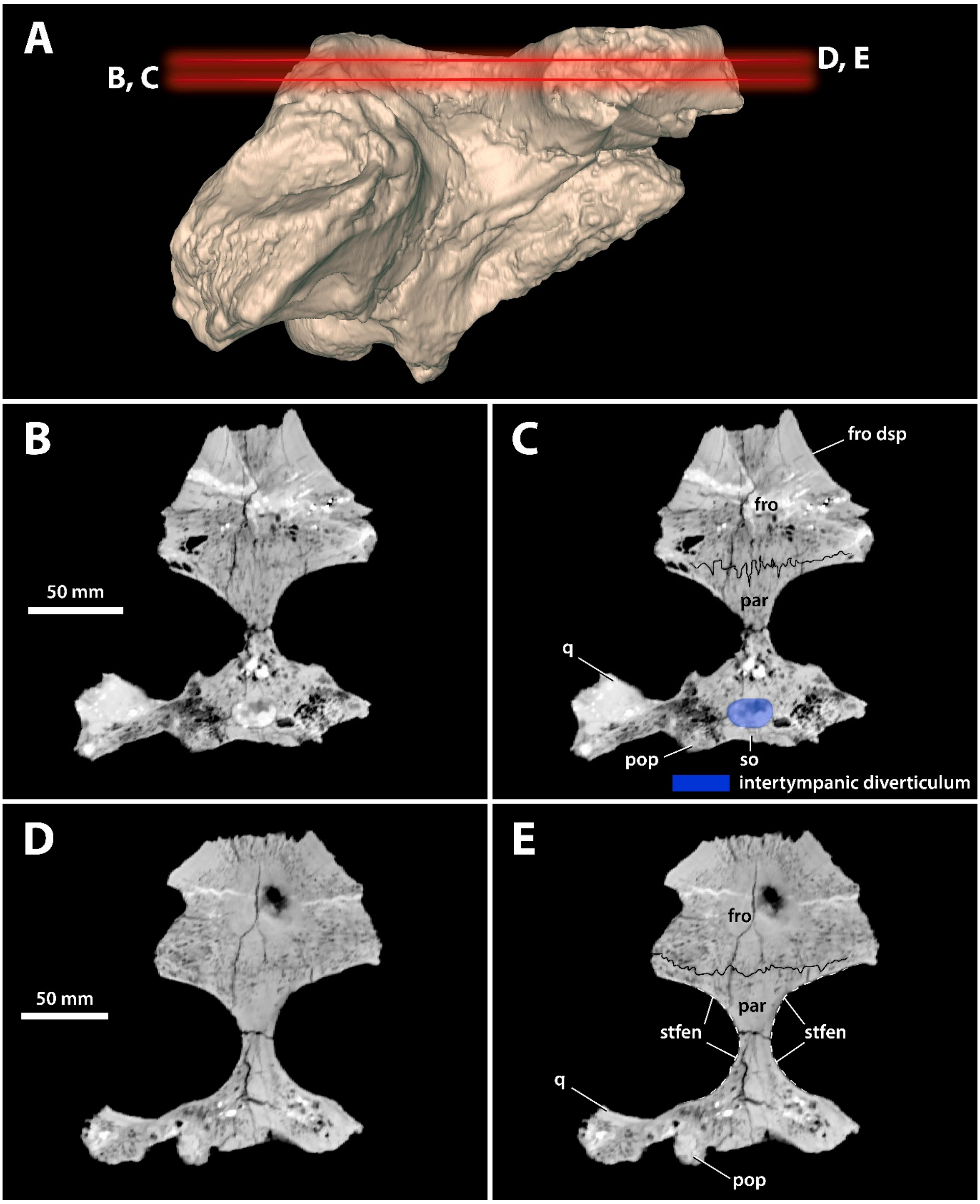

Figure S1.8 *Gunggamarandu maunala* gen. et sp. nov., QMF14.548, holotype. (A) Digital model of the cranial table in right lateral view, with the red lines indicating the levels where the screenshots of the axial slices in B–E were taken. Non-annotated axial slices in (B) and (D), and annotated axial slices in (C) and (E). Abbreviations: **fro**, frontal; **fro dsp**, frontal descending process; **par**, parietal; **pop**, postoccipital process of the supraoccipital; **q**, quadrate; **so**, supraoccipital; **stfen**, supratemporal fenestra.

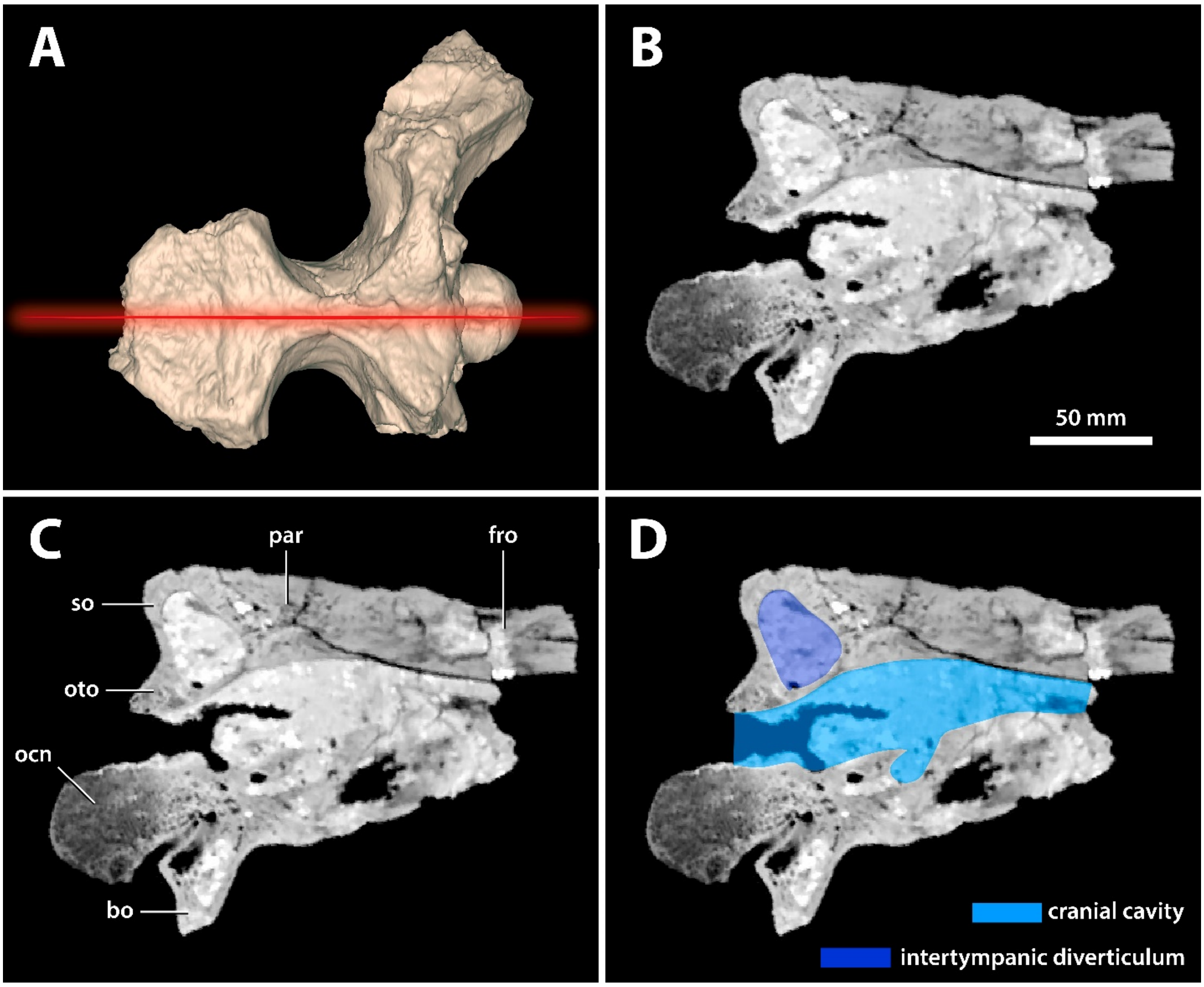

Figure S1.9 ***Gunggamarandu maunala*** **gen. et sp. nov., QMF14.548, holotype.** (A) Digital model of the specimen in dorsal view, with the red line indicating the level where the screenshot of the sagittal slice in B–D was taken. Non-annotated sagittal slice in (B), and annotated sagittal slice in (C) and (D). Abbreviations: **bo**, basioccipital; **fro**, frontal; **ocn**, occipital condyle; **oto**, otoccipital; **par**, parietal; **so**, supraoccipital.

Prootics

Unfortunately, no description can be provided of the prootics. Whether they have any exposure around the trigeminal fossae remains unknown, as they cannot be distinguished on the external braincase walls. At least some portions of the prootics still remain in QMF14.548 as

indicated by the CT scans, although the insufficient resolution combined with the extensive matrix filling of the endocranial cavities prevents meaningful assessment.

Supraoccipital

The supraoccipital (**Figs. S1.1**, **S1.4**, **S1.8B–S1.8E** and **S1.9**) is virtually complete and is also one of the best-preserved elements of QMF14.548. Since the supraoccipital is fully exposed in occipital view, most of this description is based on the external surface (i.e., the occipital lamina) of the element. As stated above (see "Parietal" subsection), it is unknown if the supraoccipital had a dorsal exposure on the cranial table. Dorsally, the supraoccipital contacts the parietal, and ventrally and ventrolaterally the otoccipitals. The supraoccipital of *G. maunala* is distinctive and is of great diagnostic value for the taxon. In occipital aspect (**Fig. S1.4**), the supraoccipital is quite wide, having a smooth and unornamented surface. Furthermore, the supraoccipital lacks a nuchal crest and its occipital surface is markedly convex. Due to its convex occipital surface, the supraoccipital somewhat overhangs the occipital region ventral to it. However, ventral to this wide convexity, at the medial contact between the supraoccipital and otoccipitals, the narrow remaining surface is flat to faintly concave. The supraoccipital-otoccipital suture is long and arch-like, giving a very wide and shallow U-shaped appearance to the supraoccipital on the occiput. The postoccipital processes, located dorsolaterally on the occipital surface of the supraoccipital, are very large and widely spaced from each other (~37 mm distance between the left and right postoccipital process; character 229, state 1). The prominent postoccipital process on the right side is almost complete and projects posteriorly from the cranium. Although the left process is mostly broken off, its position and approximate size are given away by the scarred broken surface it occupied. The size of the postoccipital processes is such that the right process is well-exposed when the cranium is observed in dorsal aspect (**Fig. S1.1**). The shape of the right postoccipital process is sub-spherical, but with a flattened ventral surface, composing most of the right lateral occipital surface of the supraoccipital. Spreading medially from each postoccipital process is a short but very

blunt ridge that becomes flush with the medial-most region of the supraoccipital's occipital lamina. Internally, the CT scans reveal an expansive intertympanic diverticulum occupying the supraoccipital (**Figs. S1.8B**, **S1.8C** and **S1.9B–S1.9D**). As rendered, the intertympanic diverticulum has a transverse width of ~50 mm, an anteroposterior length of ~29 mm and a dorsoventral height of ~42 mm.

Otoccipitals

While incomplete, substantial portions of the otoccipitals (opistothics-exoccipitals) still remain in QMF14.548 (**Figs. S1.1**, **S1.2**, **S1.4** and **S1.9**). The left otoccipital is broken-off laterally, while the right otoccipital is more complete, missing only its paroccipital process (**Fig. S1.4**). The otoccipitals are observable on the occipital surface of the skull and have gently concave, smooth external surfaces, albeit affected by thin cracks as well as a small perforation on the right element. The otoccipitals meet dorsomedially to the foramen magnum (their medial sutural contact having a length of ~14 mm; character 196, state 0) thus excluding the supraoccipital from the latter's margins. Ventrally and ventromedially, the otoccipitals contact the basioccipital. Contact with the right squamosal is evident on the right side, with a faintly visible part of the squamosal-otoccipital sutural scar spread in a dorsomedial to ventrolateral direction. Based on this, it can be inferred that the posterior process of the squamosal was quite long (character 158, state 1). The otoccipitals comprise the dorsolateral portions of the occipital condyle neck without contributing to the condyle itself. On the dorsal surface of the occipital condyle neck, the otoccipitals are in medial contact with the basioccipital. The sutural contact with the latter is also visible on the left lateral side of the occipital condyle neck, although the entirety of the suture is not distinguishable, which unfortunately leaves the full ventral extent of the otoccipital's descending process undetermined. Laterally, the right otoccipital is still in contact with the right quadrate.

Several cranial nerve and vasculature foramina open on the otoccipitals. These are the hypoglossal (cranial nerve XII) foramina, the common foramina for cranial nerves IX-XI and their

associated vessels (i.e., metotic foramina, or sometimes referred as the foramina vagi; e.g., Iordansky, 1973; Bona *et al*., 2017; Herrera *et al*., 2018), and the posterior carotid foramina. Unfortunately, their corresponding nerve and vasculature canals could not be digitally segmented. The smallest of these foramina on the otoccipitals are the hypoglossal. Each descending pillar of the otoccipital (*sensu* Kley *et al*., 2010) is pierced by two hypoglossal foramina, an anterior and posterior. Both anterior and posterior hypoglossal foramina are visible on the left descending pillar of the otoccipital, whereas on the right pillar only the posterior is exposed from the matrix. The hypoglossal foramina on the left descending pillar are sub-circular and positioned one in front of the other. Externally on the occiput, the small anterior hypoglossal foramina (each being ~1 mm in diameter) are found immediately medial to the metotic and are not enclosed within hypoglossal fossae. The posterior hypoglossal foramina are ample and circular (~4 mm in diameter, although the left posterior hypoglossal foramen has a more oval shape), located laterally to the ventral margin of the foramen magnum, or medially to the metotic and anterior hypoglossal foramina.

The largest foramina that exit on the otoccipitals are the metotic. Each metotic foramen is found laterally and nearly on the same transverse plane to the hypoglossal foramina, and is transversely elongated with a width of ~17 mm and a dorsoventral height of ~7 mm.

Ventral to the hypoglossal foramina, ventromedial to the metotic foramen, and lateral to the basioccipital-otoccipital suture at the occipital condyle neck is the posterior carotid foramen. Similarly to the posterior hypoglossal, each posterior carotid foramen has a sub-circular shape, however, it is larger than the aforementioned by having a diameter of ~6 mm.

Basioccipital

The basioccipital is incomplete, and the preservational condition of QMF14.548 allows description only of the occipital condyle and basioccipital plate (**Figs. S1.1–S1.4** and **S1.9**). The basioccipital is incomplete ventrally, particularly at its right ventrolateral side. The dorsomedial portion of the basioccipital bears the relatively short but stout occipital condyle neck.

Dorsomedially on the occipital condyle neck, the basioccipital is concave (i.e., forms a median groove) and laterally delimited by the basioccipital-otoccipital sutures that run within the foramen magnum. Distally on the occipital condyle neck, the basioccipital forms the occipital condyle. The condyle is very large (~53 mm transverse width, ~47 mm dorsoventral height) and sub-spherical, with the thin outer-most bone layer stripped-off of the condylar surface in certain places. The condyle is oriented posteriorly and slightly ventrally. There is a large vascular foramen immediately ventral to the occipital condyle neck. A small concavity is also present on the ventral surface of the basioccipital plate, ventrolateral to the occipital condyle. As preserved, the basioccipital plate is oriented posteriorly (character 170, state 1).

Basisphenoid

The presence of the basisphenoid in QMF14.548 can be inferred, although no sutural traces are evident anywhere on the basicranium in order to describe its morphology and relationships with the other cranial elements.

Splanchnocranial bones

Quadrates

Neither quadrate is complete, with the one on the left side almost entirely missing, bar for its dorsomedial-most portion (i.e., the quadrate dorsal primary head *sensu* Kley *et al*., 2010). The more complete right quadrate preserves most of its dorsal primary head, as well as its anterodorsal process, pterygoid process and part of the quadrate body, all primarily assessable in ventral and anterior views (**Figs. S1.1–S1.8**). The preserved external surfaces of the quadrates are smooth, although significantly affected by cracks medially and ventromedially at their contact with the laterosphenoids and pterygoids. Unfortunately, the sutures between these elements are not

distinguishable either. The dorsal primary heads of both quadrates, forming the posterior walls of the supratemporal fossae and ventral to the temporoorbital foramina have convex external surfaces. One of the most conspicuous features on the ventral surface of the right quadrate are the extremely well-developed crests A and B’ (*sensu* Iordansky, 1964). Crest A is not preserved in its entirety, but in comparison to crest B’, crest A is much wider, and very blunt (**Fig. S1.2**). Crest B’ (**Figs. S1.2** and **S1.3**) is in the form of a highly acute ridge, being thin and with a sharp edge. When observed ventrally, both crests A and B’ are anteromedially aligned, however, the anterior edge of crest B’ bluntly curves medially, whereas crest A appears to be nearly straight. In occipital view, the pterygoid process of the right quadrate is exposed for a significant length ventral to the otoccipital (character 214, state 1; **Fig. S1.4**).

Endocranial morphology

Brain endocast

The entirety of the brain endocast (**Figs. S1.10** and **S1.11**) was digitally segmented from the CT scan, except for the olfactory bulbs and the anterior half of the olfactory tract endocasts, which are missing. As rendered, the digital endocast is generally devoid of significant deformations, with an obvious exception being a very slight distortion dorsally on the telencephalic region of the prosencephalon approximating with the junction between the cerebral hemispheres and olfactory tract endocasts. However, it may be that the dimensions of the telencephalic region are inaccurate due to endocranial compression at that level. This is suspected primarily because the width of the olfactory tract endocast is ~14 mm, whereas the width of the olfactory tract trough between the descending processes of the frontal is ~21 mm (see “Frontal” subsection above). Normally, it would be expected that the width of the olfactory tract endocast would equal the width between the descending processes of the frontal. This compression likely affects the rest of the telencephalic portion which incorporates the section that encompassed the cerebral hemispheres.

Aside from this caveat, we are confident that the rest of the segmented brain endocast reflects its accurate proportions. Measurements of the endocast are given in **Fig. S1.11**.

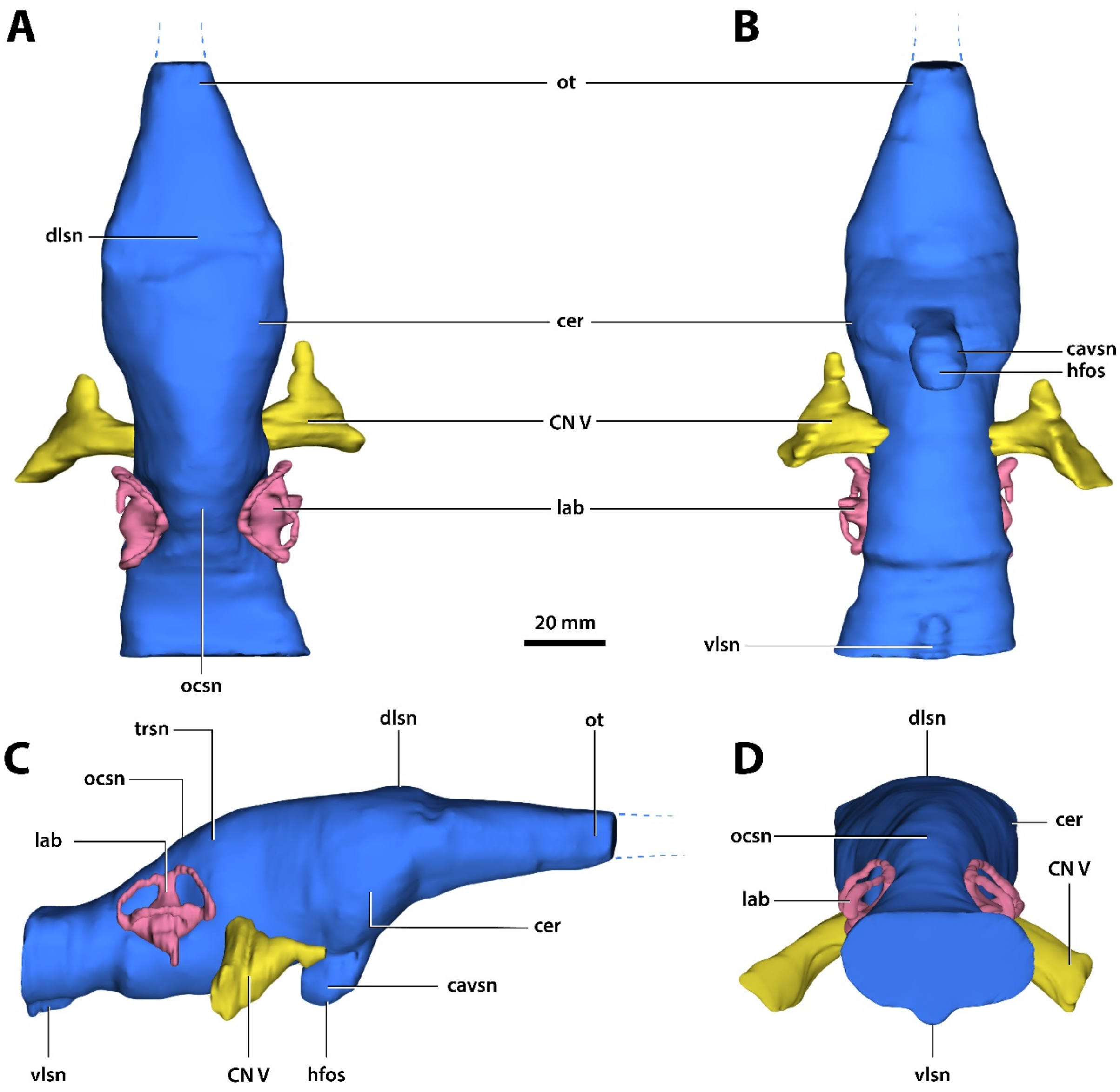

Figure S1.10 ***Gunggamarandu maunala*** **gen. et sp. nov., QMF14.548, holotype.** Digitally segmented endocranial structures in (A) dorsal, (B) ventral, (C) right lateral, and (D) occipital views. Abbreviations: **cavsn**, cavernous dural venous sinus (endocast); **cer**, cerebrum (endocast); **CN V**, trigeminal nerve canal; **dlsn**, dorsal longitudinal dural venous sinus (endocast); **hfos**, hypophyseal fossa (endocast); **lab**, endosseous labyrinth; **ocsn**, occipital dural venous sinus (endocast); **ot**, olfactory tract (endocast); **trsn**, transverse dural venous sinus (endocast); **vlsn**, ventral longitudinal dural venous sinus (endocast).

As in other crocodylomorphs (e.g., Hopson, 1979; Witmer *et al.*, 2008; Kley *et al.*, 2010; Sertich & O'Connor, 2014; Jirak & Janacek, 2017; Serrano-Martínez *et al.* 2019, 2020; Ristevski *et al.*, 2020), the brain endocast of QMF14.548 does not represent a precise reflection of the actual brain, as the brain itself was not tightly appressed to the braincase; rather, the thick dura mater and dural venous sinuses that enveloped the soft tissue brain are responsible for much of the endocasts contours. Since QMF14.548 is a specimen of a very large adult, its endocast almost certainly paints even less of the actual brain shape than it would have for an ontogenetically younger individual (see Jirak & Janacek, 2017). Nevertheless, the brain endocast still provides highly useful information on the braincase morphology, and to a certain degree, the morphology of the brain itself.

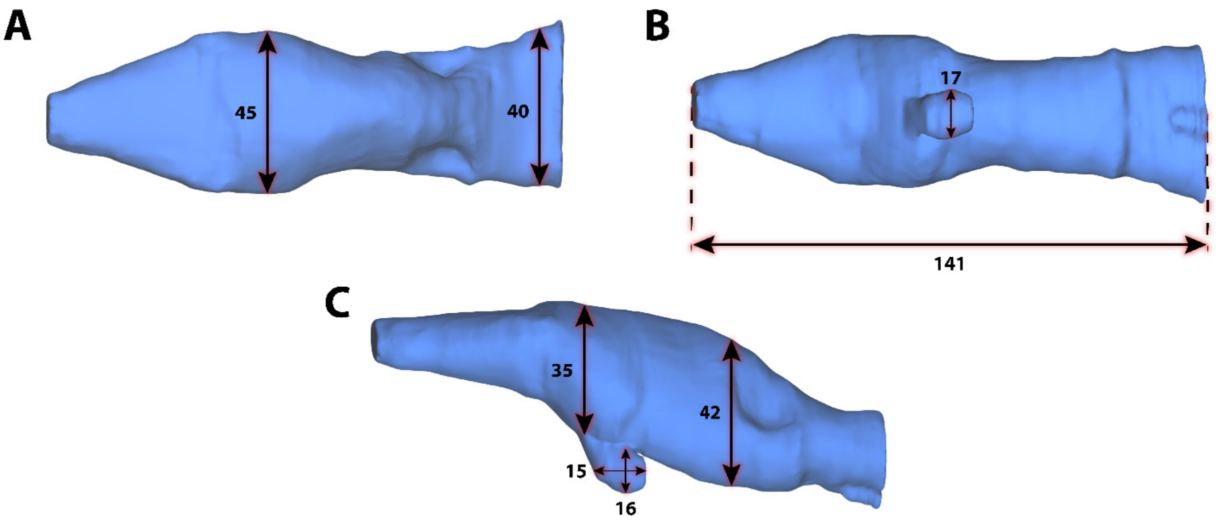

Figure S1.11 ***Gunggamarandu maunala*** **gen. et sp. nov., QMF14.548, holotype, brain endocast measurements.** Digitally segmented brain endocast in (A) dorsal, (B) ventral, and (C) left lateral views. Parameters are given in mm and were measured in Mimics 22.0.

As segmented, the brain endocast's shape generally resembles that of some mesoeucrocodylians, both extant and extinct (Franzosa, 2004; Witmer *et al.*, 2008; Kawabe *et al.*, 2009; Bona *et al.*, 2017; Jirak & Janacek, 2017; Pierce *et al.*, 2017; Serrano-Martínez *et al.* 2019,

2020; Fonseca *et al*., 2020). In lateral aspect (**Figs. S1.10C** and **S1.11C**), the brain endocast is elongated, with relatively unpronounced cephalic (forebrain-midbrain) and pontine (midbrain-hindbrain) flexures, and a smoothly sinusoidal dorsal contour. The endocast is confluent between the prosencephalon (forebrain) and mesencephalon (midbrain) regions, without a marked differentiation between the two. The endocast is widest at the level that corresponds with the cerebral hemispheres. Anteriorly from the cerebral endocast, the telencephalic portion gradually tapers anteriorly as it extends as the olfactory tract. Although not particularly salient on the endocast, the dorsal longitudinal dural venous sinus overlaid the brain (see Witmer *et al*., 2008 and Porter *et al*., 2016). The most significant feature ventrally on the prosencephalon endocast, or on its diencephalic region, is the hypophyseal (=pituitary) fossa along with its associated cavernous dural venous sinus endocast (**Figs. S1.10B** and **S1.10C**; Sedlmayr, 2002; Porter *et al*., 2016). More superficial structures, such as the impressions of the occipital and ventral longitudinal dural venous sinuses are relatively easy to discern. Thus, the occipital longitudinal dural venous sinus impression is most easily perceptible along the dorsal region of the endocast, widely overlying the sections where the optic tectum, cerebellum and dorsal part of the medulla were located (**Figs. S1.10A**, **S1.10C** and **S1.10D**). No impressions of the sphenoparietal dural venous sinus are discernable on the endocast. Near the junction of the mesencephalon and rhombencephalon regions, subtle anterolateral widenings hint at the impressions of the transverse sinus (**Fig. S1.10C**). The ventral longitudinal dural venous sinus impression is distinguishable as a short convexity along the ventral part of the myelencephalon (**Figs. S1.10B– S1.10D**). The most unique feature of the QMF14.548 brain endocast is the shape of the myelencephalic region of the rhombencephalon (which contained the remainder of the medulla oblongata, ventral longitudinal dural venous sinus and the occipital sinus) – this part of the endocast is dorsoventrally compressed and transversely elongated, with its cross-sectional outline mirroring the obround foramen magnum (**Fig. S1.10D**). Amongst crocodylomorph endocasts available to us for comparison via

contemporary published literature, the shape of the foramen magnum as well as the myelencephalon endocast appear unique to *G. maunala*.

Trigeminal nerve canals, trigeminal foramina and fossae

The only cranial nerve canals that could be segmented from the CT scan data are the trigeminal (i.e., cranial nerve V; **Fig. S1.10**). The trigeminal nerve canals are very large, tapered proximally at their "roots" on the brain endocast and progressively widen distally, attaining their greatest width at their exits on the braincase walls corresponding with the endocasts of the trigeminal (Gasserian) ganglia. Each of the two projects laterally from the brain endocast at an angle of approximately 35˚. Externally, they exit the braincase through the trigeminal foramina, or more specifically, the maxillomandibular foramina (for the maxillary and mandibular branches of the trigeminal foramen). Most canals relating to the several trigeminal nerve branches could not be reliably discerned from the CT scans, although it is likely that the digitally segmented trigeminal nerve endocast corresponds with the maxillary (V_2) and mandibular (V_3) branches of the nerve.

The trigeminal foramina (=foramina ovales; openings for cranial nerves V; **Fig. S1.7**) are located on the ventrolateral walls of the braincase. Both trigeminal foramina have diameters of ~12 mm and are filled with matrix, although the left foramen is better defined than the right. Thanks to the less extensive matrix covering, it is observable that the left trigeminal foramen is encircled by a shallow fossa (trigeminal fossa) that would have lodged the trigeminal ganglion. On the right side, dorsal to the trigeminal foramen (and separated from it by the caudal bridge of the laterosphenoid) is the ample sized and sub-oval supraorbital foramen (**Fig. S1.7**). At the anterior margin of the supraorbital foramen is a shallow and short anterodorsally directed supraorbital groove (*sensu* Holliday & Witmer, 2009). The supraorbital foramen has an anteroposterior length of ~7 mm and dorsoventral height of ~4 mm, and like all other externally exposed cranial foramina, it too is filled with matrix. The supraorbital foramen on the left side is not visible. Neither canal for the supraorbital nerve could be detected and digitally segmented from the scans.

Endosseous labyrinths

The endosseous labyrinths of the inner ears (*sensu* Witmer *et al.*, 2008) were digitally segmented, with the right labyrinth being complete and virtually undistorted (**Figs. S1.10** and **S1.12**). Only the vestibular apparatus (i.e., the semicircular canals and vestibule) could be segmented of the left endosseous labyrinth, except for some of its lateral (horizontal) semicircular canal. Hence, this description is based on the complete right endosseous labyrinth.

By following the measuring instructions proposed by Pierce *et al.* (2017), the right endosseous labyrinth has a height of ~27 mm, width of ~24 mm, and the cochlear duct has a length of ~9 mm. The vestibular apparatus is sub-pyramidal in shape and forms the dorsal component of the endosseous labyrinth, with the cochlear duct extending lateroventrally from the former. The vestibule (which contained the utricle and sacculus) is the most voluminous part of the vestibular apparatus. Medially on the vestibular apparatus is the dorsally rising common crus (crus communis), and the anterior and posterior semicircular canals meet at its dorsal end. The common crus is vertical and longer anteroposteriorly (~4 mm) than mediolaterally (~2 mm). The anterior and posterior semicircular canals are very faintly curved, with the anterior canal being slightly longer than the posterior (~17 mm and ~15 mm respectively). Additionally, the vacant space between the anterior semicircular canal and the common crus is slightly larger than the space between the crus and the posterior semicircular canal, which gives an appearance that the labyrinth is more expanded anterolaterally. At ~13 mm, the lateral semicircular canal is the shortest of the three, yet all semicircular canals have diameters of ~2 mm. Impressions of the ampullae, particularly those of the anterior and posterior semicircular canals, are not especially distinct on the endosseous labyrinth. The ampulla of the lateral semicircular canal has left the most conspicuous impression, notable as a subtle dilation just anterior to the lateral semicircular canal. The endosseous cochlear duct extends lateroventrally from the vestibular apparatus and tapers towards its ventral apex, where the lagena would have been. Observed in lateral and medial aspects, the cochlear duct has a slight anterior bend.

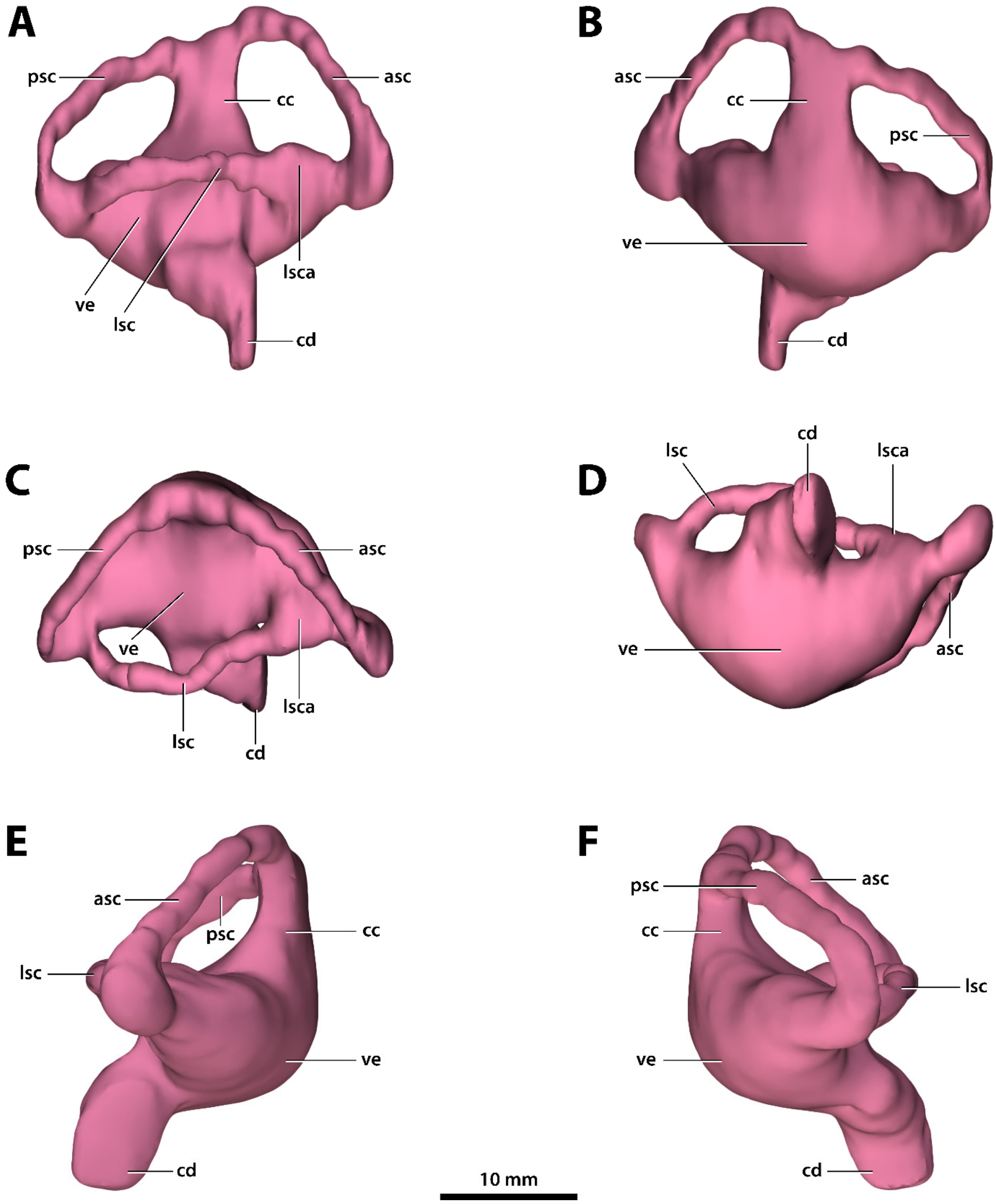

Figure S1.12 ***Gunggamarandu maunala*** **gen. et sp. nov., QMF14.548, holotype, right endosseous labyrinth.** (A) Lateral, (B) medial, (C) dorsal, (D) ventral, (E) anterior, and (F) posterior views. Abbreviations: **asc**, anterior semicircular canal (endocast); **cc**, common crus (endocast); **cd**, cochlear duct (endocast); **lsc**, lateral semicircular canal (endocast); **lsca**, ampulla of lateral semicircular canal (endocast); **psc**, posterior semicircular canal (endocast); **ve**, vestibule (endocast).

INSTITUTIONAL ABBREVIATION

QM, Queensland Museum, Brisbane, Queensland, Australia (F, fossil)

REFERENCES

Bona, P., Carabajal, A. P., & Gasparini, Z. (2017). Neuroanatomy of *Gryposuchus neogaeus* (Crocodylia, Gavialoidea): a first integral description of the braincase and endocranial morphological variation in extinct and extant gavialoids. *Earth and Environmental Science Transactions of the Royal Society of Edinburgh*, *106*(4), 235–246.

Brochu, C. A. (2007). Systematics and taxonomy of Eocene tomistomine crocodylians from Britain and northern Europe. *Palaeontology*, *50*(4), 917–928.

Buckland, W. (1836). *Geology and Mineralogy Considered with Reference to Natural Theology*. Pickering, London, 618 pp.

Busbey, A. B. III. (1989). Form and function of the feeding apparatus of *Alligator mississippiensis*. *Journal of Morphology*, *202*(1), 99–127.

Dufeau, D. L., & Witmer, L. M. (2015). Ontogeny of the middle-ear air-sinus system in *Alligator mississippiensis* (Archosauria: Crocodylia). *PLoS ONE*, *10*(9), e0137060.

Fonseca, P. H. M., Martinelli, A. G., da Silva Marinho, T., Ribeiro, L. C. B., Schultz, C. L., & Soares, M. B. (2020). Morphology of the endocranial cavities of *Campinasuchus dinizi* (Crocodyliformes: Baurusuchidae) from the Upper Cretaceous of Brazil. *Geobios*, *58*, 1–16.

Franzosa, J. W. (2004). *Evolution of the brain in Theropoda (Dinosauria)*. Unpublished PhD Thesis, Faculty of the Graduate School of The University of Texas at Austin, U. S. A., 357 pp.

Herrera, Y., Leardi, J. M., & Fernández, M. S. (2018). Braincase and endocranial anatomy of two thalattosuchian crocodylomorphs and their relevance in understanding their adaptations to the marine environment. *PeerJ*, *6*, e5686.

Holliday, C. M., & Witmer, L. M. (2009). The epipterygoid of crocodyliforms and its significance for the evolution of the orbitotemporal region of eusuchians. *Journal of Vertebrate Paleontology*, *29*(3), 715–733.

Holliday, C. M., Porter, W. R., Vliet, K. A., & Witmer, L. M. (2020). The frontoparietal fossa and dorsotemporal fenestra of archosaurs and their significance for interpretations of vascular and muscular anatomy in dinosaurs. *The Anatomical Record*, *303*(4), 1060–1074.

Hopson, J. A. (1979). Paleoneurology. Pp. 39–146 in C. Gans (ed.) *Biology of the Reptilia, 9: Neurology A*, Academic Press, New York.

Iordansky, N. N. (1964). The jaw muscles of the crocodiles and some relating structures of the crocodilian skull. *Anatomischer Anzeiger*, *115*, 256–280.

Iordansky, N. N. (1973). The skull of the Crocodilia. Pp. 201–262 in C. Gans & T. Parsons (eds.) *Biology of the Reptilia*, *4*, Academic Press, London.

Jirak, D., & Janacek, J. (2017). Volume of the crocodilian brain and endocast during ontogeny. *PLoS ONE*, *12*(6), e0178491.

Jonet, S., & Wouters, G. (1977). *Maroccosuchus zennaroi*, crocodilien Eusuchien nouveau des Phosphates du Maroc. *Notes et Mémoires du Service Géologique du Maroc*, *38*, 177–202.

Jouve, S. (2016). A new basal tomistomine (Crocodylia, Crocodyloidea) from Issel (Middle Eocene; France): palaeobiogeography of basal tomistomines and palaeogeographic consequences. *Zoological Journal of the Linnean Society*, *177*(1), 165–182.

Jouve, S., Bouya, B., Amaghzaz, M., & Meslouh, S. (2015). *Maroccosuchus zennaroi* (Crocodylia: Tomistominae) from the Eocene of Morocco: phylogenetic and palaeobiogeographical implications of the basalmost tomistomine. *Journal of Systematic Palaeontology*, *13*(5), 421–445.

Kälin, J. A. (1933). Beiträge zur vergleichenden Osteologie des Crocodilidenschädels. *Zoologische Jahrbücher*, *57*(4), 535–714.

Kawabe, S., Shimokawa, T., Miki, H., Okamoto, T., & Matsuda, S. (2009). A simple and accurate method for estimating the brain volume of birds: possible application in paleoneurology. *Brain, Behavior and Evolution*, *74*(4), 295–301.

Kley, N., Sertich, J., Turner, A., Krause, D., O'Connor, P., & Georgi, J. (2010). Craniofacial morphology of *Simosuchus clarki* (Crocodyliformes: Notosuchia) from the Late Cretaceous of Madagascar. *Journal of Vertebrate Paleontology*, *30*(S1), 13–98.

Montefeltro, F. C., Andrade, D. V., & Larsson, H. C. (2016). The evolution of the meatal chamber in crocodyliforms. *Journal of Anatomy*, *228*(5), 838–863.

Mook, C. C. (1955). Two new genera of Eocene crocodilians. *American Museum Novitates*, *1727*, 1–4.

Pierce, S. E., Williams, M., & Benson, R. B. J. (2017). Virtual reconstruction of the endocranial anatomy of the early Jurassic marine crocodylomorph *Pelagosaurus typus* (Thalattosuchia). *PeerJ*, *5*, e3225.

Piras, P., Delfino, M., Del Favero, L., & Kotsakis, T. (2007). Phylogenetic position of the crocodylian *Megadontosuchus arduini* and tomistomine palaeobiogeography. *Acta Palaeontologica Polonica*, *52*(2), 315–328.

Porter, W. R., Sedlmayr, J. C., & Witmer, L. M. (2016). Vascular patterns in the heads of crocodilians: blood vessels and sites of thermal exchange. *Journal of Anatomy*, *229*(6), 800–824.

Ristevski, J., Yates, A. M., Price, G. J., Molnar, R. E., Weisbecker, V., & Salisbury, S. W. (2020). Australia's prehistoric 'swamp king': revision of the Plio-Pleistocene crocodylian genus *Pallimnarchus* de Vis, 1886. *PeerJ*, *8*, e10466.

Sedlmayr, J. C. (2002). *Anatomy, evolution, and functional significance of cephalic vasculature in Archosauria*. Unpublished PhD Thesis, Ohio University, Athens, 398 pp.

Serrano-Martínez, A., Knoll, F., Narváez, I., Lautenschlager, S., & Ortega, F. (2019). Brain and pneumatic cavities of the braincase of the basal alligatoroid *Diplocynodon tormis* (Eocene, Spain). *Journal of Vertebrate Paleontology*, *39*(1), e1572612.

Serrano-Martínez, A., Knoll, F., Narváez, I., Lautenschlager, S., & Ortega, F. (2020). Neuroanatomical and neurosensorial analysis of the Late Cretaceous basal eusuchian *Agaresuchus fontisensis* (Cuenca, Spain). *Papers in Palaeontology*, DOI: 10.1002/spp2.1296.

Sertich, J. J. W., & O'Connor, P. M. (2014). A new crocodyliform from the middle Cretaceous Galula Formation, southwestern Tanzania. *Journal of Vertebrate Paleontology*, *34*(3), 576–596.

Toula, F., & Kail, J. A. (1885). Über einen Krokodil–Schädel aus den Tertiärablagerungen von Eggenburg in Niederösterreich: eine paläontologische studie. *Denkschriften der Kaiserlichen Akademie der Wissenschaften von Wien, Mathematisch–naturwissenschaftliche Classe*, *50*, 299–355.

Witmer, L. M., Ridgely, R. C., Dufeau, D. L., & Semones, M. C. (2008). Using CT to peer into the past: 3D visualization of the brain and ear regions of birds, crocodiles, and nonavian dinosaurs. Pp. 67–87 in H. Endo & R. Frey (eds.) *Anatomical Imaging*, Springer, Tokyo.

de Zigno, A. (1880). Sopra un cranio di coccodrillo scoperto nel terreno eoceno del Veronese. *Atti della Reale Accademia Lincei, Memorie della Classe di Scienze Fisiche, Matematiche e Naturali, Serie 3*, *5*, 65–72.
