# Supplementary material for: First record of a tomistomine crocodylian from Australia
Source: Sci Rep. 2021 Jun 9;11:12158. doi: 10.1038/s41598-021-91717-y (PMC8190066; doi:10.1038/s41598-021-91717-y)
Supplement: Supplementary file 2 — Supplementary Information 2. [file 41598_2021_91717_MOESM2_ESM.pdf]

SUPPLEMENTARY MATERIAL FOR

FIRST RECORD OF A TOMISTOMINE CROCODYLIAN FROM AUSTRALIA

SUPPLEMENTAL DOCUMENT S2: ADDITIONAL INFORMATION ON THE PHYLOGENETIC DATASET AND PHYLOGENETIC RESULTS

by JORGO RISTEVSKI[1*], GILBERT J. PRICE[2], VERA WEISBECKER[1, 3] and STEVEN W. SALISBURY[1]

[1]School of Biological Sciences, The University of Queensland, Brisbane, 4072, Queensland, Australia

[2]School of Earth and Environmental Sciences, The University of Queensland, Brisbane, 4072, Queensland, Australia

[3]College of Science and Engineering, Flinders University, Bedford Park 5042, South Australia, Australia

***Corresponding Author:**

Jorgo Ristevski[1]

School of Biological Sciences, Goddard Building (Building 8), The University of Queensland, Brisbane 4072, Queensland, Australia

Email address: j.ristevski@uq.net.au

TABLE OF CONTENTS

Taxon list **2**

Links to the examined digital specimens 20

Character list and taxon matrix – modifications and updates **22**

Morphological character list **24**

Explanation for scoring character 229 54

Phylogenetic analyses **57**

Results 57

Equal Weighting analysis 57

Implied Weighting analyses 64

Scored characters for the Australian crocodyliform operational taxonomic units 77

Institutional abbreviations **78**

References **79**

TAXON LIST

Below is a list (in alphabetical order) of all operational taxonomic units (OTUs) included in the taxon matrix. The sources, both from the literature as well as examined specimens, used for comparative anatomy (see the main text of "First record of a tomistomine crocodylian from Australia") and scoring the new morphological character (character 229) are also provided. An asterisk '*' designates a type specimen. A 'c' stands for cast, while a '*c' stands for cast of the type specimen. A 'p' indicates that high-quality photographs were used for assessment. Type specimens examined from photographs are indicated with a '*p'. A 'd' stands for digital model whilst '*d' stands for digital model of the type specimen, available online. Links to the examined digital models can be found below.

- *Acynodon adriaticus* Delfino *et al.*, 2008b

  Literature source: Delfino *et al.* (2008b)

- *Acynodon iberoccitanus* Buscalioni *et al.*, 1997

  Literature sources: Buscalioni *et al.* (1997); Martin (2007); Ősi (2014)

- *Agaresuchus fontisensis* Narváez *et al.*, 2016

  Literature sources: Narváez *et al.* (2016); Serrano-Martínez *et al.* (2020)

- *Agaresuchus subjuniperus* (Puértolas-Pascual *et al.*, 2014)

  Literature sources: Puértolas-Pascual *et al.* (2014); Narváez *et al.* (2016); Mateus *et al.* (2019)

- *Alligator mcgrewi* Schmidt, 1941

  Literature sources: Brochu (1999, 2004b)

- *Alligator mefferdi* Mook, 1946

Literature sources: Mook (1946); Brochu (1999)

- *Alligator mississippiensis* (Daudin, 1802)

  Examined specimens: QMJ4850, unregistered specimen at UQ, UF35129[d]

  Literature sources: Mook (1921c); Wermuth (1953); Iordansky (1973); Brochu (1999, 2004b); Salisbury (2001); Gold (2011); de Iuliis & Pulerà (2011); Dufeau & Witmer (2015); Drumheller *et al.* (2016); Klein (2016); Porter *et al.* (2016); Sookias (2020; supplementary)

- *Alligator olseni* White, 1942

  Literature sources: White (1942); Brochu (1999)

- *Alligator prenasalis* (Loomis, 1904)

  Literature sources: Mook (1932); Brochu (1999, 2004b)

- *Alligator sinensis* Fauvel, 1879

  Examined specimen: UF-H-105540[d]

  Literature sources: Mook (1923b); Wermuth (1953); Iordansky (1973); Brochu (1999); Drumheller *et al.* (2016); Sookias (2020; supplementary)

- *Alligator thomsoni* Mook, 1923a

  Literature sources: Mook (1923a); Brochu (1999)

- *Allodaposuchus precedens* Nopcsa, 1928

  Literature sources: Delfino *et al.* (2008a); Mateus *et al.* (2019); Narváez *et al.* (2020)

- *Allognathosuchus polyodon* Cope, 1873

  Literature sources: Mook (1961); Brochu (1999, 2004b)

- *Allognathosuchus wartheni* Case, 1925

Literature sources: Brochu (1999, 2004b)

- *Anteophthalmosuchus epikrator* Ristevski *et al*., 2018

Examined specimens: IRSNB R47, IWCMS 2001.446*, IWCMS 2005.127

Literature sources: Salisbury (2001); Martin *et al.* (2016); Ristevski *et al.* (2018)

- *Arambourgia gaudryi* (de Stefano, 1905)

Literature sources: Brochu (1999, 2004b)

- *Arenysuchus gascabadiolorum* Puértolas-Pascual *et al*., 2011

Literature sources: Puértolas-Pascual *et al.* (2011); Mateus *et al.* (2019)

- *Asiatosuchus germanicus* Berg, 1966

Literature source: Berg (1966)

- *Australosuchus clarkae* Willis & Molnar, 1991

Examined specimens: QMF16788*, QMF17433, QMF17983, QMF17984, QMF17985, QMF17986, QMF18102, QMF18151, QMF18152, SAM P23985[c, p], SAM P30160[p], SAM P30162[p], SAM P33761[p], SAM P33768[p], SAM P33942[c, p], SAM P36583[p], SAM P42788[p], SAM P42789[p]

Literature source: Willis & Molnar (1991)

- *Baru darrowi* Willis *et al*., 1990

Examined specimens: NTM P8695-8*, NTM P87115-15

Literature sources: Willis *et al.* (1990); Yates (2017)

- *Baru wickeni* Willis, 1997

Examined specimens: NTM P902-4, NTM P911, NTM P943-4 (P902-, P91164-), NTM P8681-14, NTM P8738-1, NTM P8778-2, NTM P8778-4, NTM P8778-5,

NTM P91164-10, NTM P91171-1, QMF16822*, QMF16823, QMF16824, QMF31070, QMF31071, QMF31072, QMF31073, QMF31074

Literature sources: Willis (1997); Yates (2017)

- *Bernissartia fagesii* Dollo, 1883

  Examined specimen: IRSNB R46*P

  Literature sources: Buffetaut (1975); Norell & Clark (1990); Salisbury (2001); Ősi (2014); Sookias (2020; supplementary); Martin *et al.* (2020)

- *Borealosuchus acutidentatus* (Sternberg, 1932)

  Literature source: Brochu (1997a)

- *Borealosuchus formidabilis* (Erickson, 1976)

  Literature sources: Erickson (1976); Brochu (1997a); Brochu *et al.* (2012)

- *Borealosuchus sternbergii* (Gilmore, 1910)

  Literature sources: Gilmore (1910); Brochu (1997a); Brochu *et al.* (2012)

- *Borealosuchus threeensis* Brochu *et al.*, 2012

  Literature source: Brochu *et al.* (2012)

- *Borealosuchus wilsoni* (Mook, 1959)

  Literature sources: Mook (1959); Brochu (1997a); Brochu *et al.* (2012)

- *Bottosaurus harlani* (Meyer, 1832)

  Literature source: Cossette & Brochu (2018)

- *Boverisuchus magnifrons* Kuhn, 1938

  Literature sources: Rossmann (2000); Brochu (2013)

- *Boverisuchus vorax* (Troxell, 1925)

  Literature sources: Langston (1975); Busbey (1977); Rossmann (2000); Brochu (2013)

- *Brachychampsa montana* Gilmore, 1911

  Literature sources: Gilmore (1911); Norell *et al.* (1994); Brochu (1999, 2004b); Sullivan & Lucas (2003)

- *Brachychampsa sealeyi* Williamson, 1996

  Literature source: Williamson (1996)

- *Brachyuranochampsa eversolei* Zangerl, 1944

  Literature source: Zangerl (1944)

- *Brochuchus pigotti* (Tchernov & Van Couvering, 1978)

  Literature sources: Tchernov & Van Couvering (1978); Conrad *et al.* (2013)

- *Caiman crocodilus* (Linnaeus, 1758)

  Examined specimens: FMNH 73711[d], QMJ2094, QMJ53058

  Literature sources: Wermuth (1953); Iordansky (1973); Brochu (1999); Gold (2011); Sookias (2020; supplementary)

- *Caiman latirostris* (Daudin, 1802)

  Literature sources: Wermuth (1953); Brochu (1999); Bona & Desojo (2011); Ősi (2014); Foth *et al.* (2018); Sookias (2020; supplementary)

- *Caiman lutescens* (Rovereto, 1912)

  Literature sources: Langston (1965); Brochu (1999)

- *Caiman yacare* (Daudin, 1802)

  Literature sources: Wermuth (1953); Brochu (1999); Mateus *et al.* (2019); Sookias (2020; supplementary)

- *Centenariosuchus gilmorei* Hastings *et al.*, 2013

  Literature source: Hastings *et al.* (2013)

- *Ceratosuchus burdoshi* Schmidt, 1938

  Literature sources: Schmidt (1938); Bartels (1984); Brochu (1999)

- "*Crocodylus*" *acer* Cope, 1882

  Literature sources: Cope (1882); Mook (1921b)

- "*Crocodylus*" *affinis* Marsh, 1871

  Literature sources: Mook (1921a); Norell & Storrs (1989)

- "*Crocodylus*" *depressifrons* de Blainville, 1855

  Literature source: Delfino & Smith (2009)

- "*Crocodylus*" *gariepensis* Pickford, 2003

  Literature source: Pickford (2003)

- "*Crocodylus*" *megarhinus* Andrews, 1905

  Literature source: Mook, 1927

- *Crocodylus acutus* Cuvier, 1807

  Literature sources: Wermuth (1953); Iordansky (1973); Brochu (2006a)

- *Crocodylus anthropophagus* Brochu *et al.*, 2010

Literature source: Brochu *et al.* (2010)

- *Crocodylus checchiai* Maccagno, 1948

  Literature sources: Hecht (1987); Brochu & Storrs (2012); Delfino *et al.* (2020)

- *Crocodylus intermedius* Graves, 1819

  Literature source: Mook (1921c)

- *Crocodylus johnstoni* Krefft, 1873

  Examined specimens: QMJ4280, QMJ22551, QMJ28895, QMJ39230, QMJ40259, QMJ45309, QMJ47916, QMJ58446, QMJ60590, QMJ60591, QMJ60592, QMJ65021, QMJ65022, QMJ65024, QMJ75835, QMJ84415, QMJ85977, QMJ86243, QMJ93435, QMJ94182, UQSSAL J9

  Literature source: Wermuth (1953)

- *Crocodylus mindorensis* Schmidt, 1935

  Literature source: Schmidt (1935)

- *Crocodylus moreletii* Duméril & Bibron, 1851

  Examined specimen: TMM M-4980[d]

  Literature sources: Wermuth (1953); Platt *et al.* (2009)

- *Crocodylus niloticus* Laurenti, 1768

  Examined specimen: QMJ58445

  Literature sources: Mook (1921c); Wermuth (1953); Sookias (2020; supplementary)

- *Crocodylus novaeguineae* (Schmidt, 1928)

  Examined specimens: QMJ5332, QMJ5664

Literature sources: Wermuth (1953); Hall & Portier (1994)

- *Crocodylus palaeindicus* Falconer, 1859

  Literature source: Mook (1933)

- *Crocodylus palustris* Lesson, 1831

  Literature source: Wermuth (1953); Iijima (2017)

- *Crocodylus porosus* Schneider, 1801

  Examined specimens: NTM R12638, QMJ5005, QMJ13443, QMJ14478, QMJ22550, QMJ29021, QMJ39231, QMJ39232, QMJ39233, QMJ39283, QMJ39284, QMJ39853, QMJ45308, QMJ47446, QMJ47447, QMJ47448, QMJ47474, QMJ48126, QMJ48127, QMJ52809, QMJ87508, QMJ87509, QMJ93238, QMJ93350, one unregistered specimen at QM, UQSSAL J7, five unregistered specimens at UQ

  Literature sources: Mook (1921c); Wermuth (1953); Richardson *et al.* (2002); Sookias (2020; supplementary)

- *Crocodylus raninus* Müller & Schlegel, 1844

  Literature source: Ross (1990)

  Remark: The status of *Crocodylus raninus* as a valid species has been controversial (see Das & Charles, 2002; Gratten, 2003; Grigg & Kirshner, 2015)

- *Crocodylus rhombifer* Cuvier, 1807

  Examined specimen: NMB AB50.0171[d]

  Literature sources: Mook (1921c); Wermuth (1953); Morgan & Albury (2013); Morgan *et al.* (1993, 2018); Sookias (2020; supplementary)

- *Crocodylus siamensis* Schneider, 1801

Examined specimen: MZB 2005-0351[d]

Literature sources: Wermuth (1953); Delfino & De Vos (2010)

- *Crocodylus thorbjarnarsoni* Brochu & Storrs, 2012

  Examined specimen: KNM-ER 1683*[d]

  Literature source: Brochu & Storrs (2012); Brochu (2020)

- *Culebrasuchus mesoamericanus* Hastings *et al.*, 2013

  Literature source: Hastings *et al.* (2013)

- *Deinosuchus riograndensis* (Colbert & Bird, 1954)

  Literature source: Colbert & Bird (1954); Cossette & Brochu (2020)

- *Diplocynodon darwini* (Ludwig, 1877)

  Literature sources: Ludwig (1877); Berg (1966)

- *Diplocynodon deponiae* (Frey *et al.*, 1987)

  Literature sources: Frey *et al.* (1987); Delfino & Smith (2012)

- *Diplocynodon hantoniensis* (Wood, 1846)

  Literature sources: Brochu (1999); Rio *et al.* (2020)

- *Diplocynodon muelleri* (Kälin, 1936)

  Literature source: Piras & Buscalioni, 2006

- *Diplocynodon ratelii* Pomel, 1847

  Literature sources: Brochu (1999); Díaz Aráez *et al.* (2017)

- *Diplocynodon tormis* Buscalioni *et al.*, 1992

  Literature sources: Jiménez Fuentes (1983); Buscalioni *et al.* (1992); Serrano-Martínez *et al.* (2019a)

- *Dollosuchoides densmorei* Brochu, 2007b

  Examined specimen: IRSNB R1748[*P]

  Literature source: Brochu (2007b)

- *Eoalligator chunyii* Young, 1964

  Literature sources: Young (1964), Wang *et al.* (2016)

- *Eocaiman cavernensis* Simpson, 1933

  Literature sources: Simpson (1933); Brochu (1999); Godoy *et al.* (2021)

- *Eogavialis africanum* (Andrews, 1901)

  Literature source: Müller (1927)

- *Eosuchus lerichei* Dollo, 1907

  Literature source: Delfino *et al.* (2005)

- *Eosuchus minor* (Marsh, 1870)

  Literature source: Brochu (2006b)

- *Eothoracosaurus mississippiensis* Brochu, 2004a

  Literature source: Brochu (2004a)

- *Euthecodon arambourgi* Ginsburg & Buffetaut, 1978

  Literature source: Ginsburg & Buffetaut (1978)

- *Gavialis gangeticus* (Gmelin, 1789)

  Literature sources: Mook (1921c); Wermuth (1953); Iordansky (1973); Tarsitano *et al.* (1989); Salisbury (2001); Brochu (2004a); Gold (2011); Pierce *et al.* (2017); Sookias (2020; supplementary)

- *Gavialis lewisi* Lull, 1944

  Literature sources: Lull (1944); Norell & Storrs (1989)

- *Gavialosuchus eggenburgensis* Toula & Kail, 1885

  Literature source: Toula & Kail (1885)

- *Globidentosuchus brachyrostris* Scheyer *et al.*, 2013

  Literature source: Scheyer *et al.* (2013)

- *Gryposuchus colombianus* (Langston, 1965)

  Literature sources: Langston (1965); Langston & Gasparini (1997); Salas-Gismondi *et al.* (2016)

- *Gunggamarandu maunala* gen. et sp. nov.

  Examined specimen: QMF14.548*

- *Harpacochampsa camfieldensis* Megirian *et al.*, 1991

  Examined specimens: NTM P87106-1*, NTM P87106-5, NTM P87106-6, NTM P87106-19, NTM P87106-20

  Literature source: Megirian *et al.* (1991)

- *Hassiacosuchus haupti* Weitzel, 1935

  Literature sources: Weitzel (1935); Brochu (1999, 2004b); Ősi (2014)

- *Hylaeochampsa vectiana* Owen, 1874

  Literature sources: Clark & Norell (1992); Salisbury & Naish (2011)

- *Iharkutosuchus makadii* Ősi *et al.*, 2007

  Literature sources: Ősi *et al.* (2007); Ősi (2008, 2014); Ősi & Weishampel (2009); Mateus *et al.* (2019)

- *Isisfordia duncani* Salisbury *et al.*, 2006

  Examined specimens: QMF34642, QMF36211*[c], QMF44320

  Literature sources: Salisbury *et al.* (2006); Syme & Salisbury (2018)

- *Jiangxisuchus nankangensis* Li *et al.*, 2019

  Literature source: Li *et al.* (2019)

- *Kalthifrons aurivellensis* Yates & Pledge, 2016

  Examined specimen: SAM P35062*[p]

  Literature source: Yates & Pledge (2016)

- *Kambara implexidens* Salisbury & Willis, 1996

  Examined specimens: QMF21116, QMF21118, QMF21131, QMF29662*, QMF29663, QMF29678, QMF29680, QMF29684, QMF29693, QMF29708, QMF29710, QMF29714, QMF30077

  Literature source: Salisbury & Willis (1996)

- *Kambara murgonensis* Willis *et al.*, 1993

  Examined specimens: QMF11625, QMF21117, QMF21119, QMF21120, QMF21122, QMF21123, QMF21124, QMF21125, QMF21126, QMF21127, QMF21128, QMF21129, QMF21130, QMF21134, QMF29665, QMF29666,

QMF29667, QMF29688, QMF29669, QMF29683, QMF29689, QMF29691, QMF29692, QMF29694, QMF29696, QMF29718, QMF30220, QMF30221

Literature sources: Willis *et al.* (1993, 1995)

- *Kambara taraina* Buchanan, 2009

  Literature source: Buchanan (2009)

- *Kentisuchus spenceri* (Buckland, 1836)

  Examined specimens: NHMUK PV R 1753[p], NHMUK PV OR 19633*[p], NHMUK PV OR 37717[p], NHMUK PV OR 38975[p], NHMUK PV OR 38990[p], NHMUK PV OR 38991[p]

  Literature source: Brochu (2007b)

- *Krabisuchus siamogallicus* Martin & Lauprasert, 2010

  Literature source: Martin & Lauprasert (2010)

- *Leidyosuchus canadensis* Lambe, 1907

  Literature sources: Lambe (1907); Brochu (1997a); Wu *et al.* (2001)

- *Lohuecosuchus mechinorum* Narváez *et al.*, 2015

  Literature source: Narváez *et al.* (2015)

- *Lohuecosuchus megadontos* Narváez *et al.*, 2015

  Literature sources: Narváez *et al.* (2015); Serrano-Martínez *et al.* (2019b)

- *Maomingosuchus petrolica* (Yeh, 1958)

  Literature sources: Li (1975); Shan *et al.* (2017)

- *Mecistops cataphractus* (Cuvier, 1824)

Examined specimen: TMM M-3529[d]

Literature sources: Mook (1921c); Wermuth (1953); Sookias (2020; supplementary)

- *Megadontosuchus arduini* (de Zigno, 1880)

  Examined specimen: MGPD 1Z*[p]

  Literature source: Piras *et al.* (2007)

- *Mekosuchus sanderi* Willis, 2001

  Examined specimens: QMF31166, QMF31186, QMF31187, QMF31188*

  Literature source: Willis (2001)

- *Mekosuchus whitehunterensis* Willis, 1997

  Examined specimens: QMF31051*, QMF31052, QMF31053, QMF31054, QMF31055

  Literature source: Willis (1997)

- *Melanosuchus niger* (Spix, 1825)

  Literature sources: Mook (1921c); Wermuth (1953); Brochu (1999); Foth *et al.* (2015); Bona *et al.* (2017); Foth *et al.* (2018); Mateus *et al.* (2019); Sookias (2020; supplementary)

- *Mourasuchus atopus* (Langston, 1965)

  Literature sources: Langston (1965, 1966)

- *Navajosuchus mooki* (Simpson, 1930)

  Literature sources: Simpson (1930); Brochu (2004b)

- *Orientalosuchus naduongensis* Massonne *et al.*, 2019

Literature source: Massonne *et al.* (2019)

- *Orthogenysuchus olseni* Mook, 1924b

  Literature sources: Mook (1924b); Brochu (1999)

- *Osteolaemus osborni* (Schmidt, 1919)

  Literature sources: Mook (1921c); Wermuth (1953); Brochu (2006a); Conrad *et al.* (2013); Mateus *et al.* (2019); Sookias (2020; supplementary)

- *Osteolaemus tetraspis* Cope, 1861

  Examined specimen: FMNH 98396[d]

  Literature sources: Mook (1921c); Wermuth (1953); Iordansky (1973); Gold (2011); Montefeltro *et al.* (2016); Sookias (2020; supplementary)

- *Pachycheilosuchus trinquei* Rogers, 2003

  Literature source: Rogers (2003)

- *Paleosuchus palpebrosus* Cuvier, 1807

  Examined specimen: RVC-JRH-PP1[d]

  Literature sources: Wermuth (1953); Brochu (1999); Mateus *et al.* (2019); Sookias (2020; supplementary)

- *Paleosuchus trigonatus* (Schneider, 1801)

  Examined specimens: NHMUK 1868.10.8.1[p]; USNM: amphibians & reptiles: 300660[d]

  Literature sources: Mook (1921c); Wermuth (1953); Brochu (1999); Mateus *et al.* (2019); Sookias (2020; supplementary)

- *Paludirex vincenti* Ristevski *et al.*, 2020a

Examined specimens: 'Geoff Vincent's specimen' (CMC2019-010 + QMF59017)*, QMF11626

- *Paratomistoma courti* Brochu & Gingerich, 2000

  Literature source: Brochu & Gingerich (2000)

- *Penghusuchus pani* Shan *et al.*, 2009

  Literature source: Shan *et al.* (2009)

- *Pietraroiasuchus ormezzanoi* Buscalioni *et al.*, 2011

  Literature source: Buscalioni *et al.* (2011)

- *Piscogavialis jugaliperforatus* Kraus, 1998

  Literature sources: Kraus (1998); Salas-Gismondi *et al.* (2016)

- *Planocrania datangensis* Li, 1976

  Literature sources: Li (1976); Brochu (2013)

- *Planocrania hengdongensis* Li, 1984

  Literature sources: Li (1984); Brochu (2013)

- *Portugalosuchus azenhae* Mateus *et al.*, 2019

  Literature source: Mateus *et al.* (2019)

- *Procaimanoidea kayi* (Mook, 1941b)

  Literature sources: Mook (1941b); Brochu (2004b)

- *Procaimanoidea utahensis* Gilmore, 1946

  Literature sources: Gilmore (1946); Brochu (2004b)

- *Prodiplocynodon langi* Mook, 1941a

    Literature source: Mook (1941a)

- *Protoalligator huiningensis* Young, 1982

    Literature source: Wang *et al.* (2016)

- *Purussaurus neivensis* (Mook, 1941c)

    Literature sources: Langston (1965); Aguilera *et al.* (2006)

- *Quinkana timara* Megirian, 1994

    Examined specimens: NTM P894-6, NTM P895-16, NTM P895-38, NTM P895-19*, NTM P2775-1, NTM P8691-3, NTM P8697-2; NTM P9464-167, NTM P9464-168, NTM P9464-169, NTM P9464-170, NTM P9464-182, NMV P179632[c]

    Literature source: Megirian (1994)

- *Rimasuchus lloydi* (Fourtau, 1920)

    Literature source: Storrs (2003)

- *Shamosuchus djadochtaensis* Mook, 1924a

    Literature sources: Mook (1924a); Pol *et al.* (2009); Turner (2015)

- *Stangerochampsa mccabei* Wu *et al.*, 1996

    Literature sources: Wu *et al.* (1996); Brochu (2004b)

- *Susisuchus anatoceps* Salisbury *et al.*, 2003

    Literature sources: Salisbury (2001); Salisbury *et al.* (2003); Frey & Salisbury (2007); Figueiredo *et al.* (2011); Leite & Fortier (2018)

- *Thecachampsa antiqua* (Leidy, 1852)

Literature source: Myrick (2001)

- *Theriosuchus pusillus* Owen, 1878b

  Literature sources: Owen (1879); Joffe (1967); Clark (1986); Salisbury (2001, 2002); Tennant *et al.* (2016); Schwarz *et al.* (2017)

- *Thoracosaurus macrorhynchus* (de Blainville, 1835)

  Literature source: Brochu (2004a)

- *Thoracosaurus neocesariensis* (de Kay, 1842)

  Literature source: Carpenter (1983); Brochu (2004a)

- "*Tomistoma*" *cairense* Müller, 1927

  Literature source: Müller (1927)

- *Tomistoma lusitanica* Antunes, 1961

  Literature source: Antunes (1961)

- *Tomistoma schlegelii* (Müller, 1838)

  Examined specimen: TMM M-6342[d]

  Literature sources: Mook (1921c); Wermuth (1953); Iordansky (1973); Tarsitano *et al.* (1989); Gold (2011); Sookias (2020; supplementary)

- *Toyotamaphimeia machikanensis* (Kamei et Matsumoto in Kobatake *et al.*, 1965)

  Literature source: Kobayashi *et al.* (2006)

- *Tsoabichi greenriverensis* Brochu, 2010

  Literature source: Brochu (2010)

- *Voay robustus* (Grandidier & Vaillant, 1872)

Examined specimens: NHMUK PV OR 2026[P], NHMUK PV R 36684[P], NHMUK PV R 36685[P]

Literature sources: Brochu (2006a, 2007a); Bickelmann & Klein (2009)

- *Wannaganosuchus brachymanus* Erickson, 1982

  Literature sources: Erickson (1982); Brochu (1999, 2004b)

Links to the examined digital specimens

The links to all below listed digital specimens are available online as of 07.07.2020.

- *Alligator mississippiensis* UF35129:

  https://sketchfab.com/3d-models/alligator-mississippiensis-uf-herp-35129-albert-bd974aa4336c4f33bf14b2e3499970f4

- *Alligator sinensis* UF-H-105540:

  https://sketchfab.com/3d-models/alligator-sinensis-uf-herp-105540-87bec849ccf54be7981bc539d30814c6

- *Caiman crocodilus* FMNH 73711:

  http://www.digimorph.org/specimens/Caiman_crocodilus/

- *Crocodylus moreletii* TMM M-4980:

  http://www.digimorph.org/specimens/Crocodylus_moreletii/

- *Crocodylus rhombifer* NMB AB50.0171:

  http://www.digimorph.org/specimens/Crocodylus_rhombifer/

- *Crocodylus siamensis* MZB 2005-0351:

https://sketchfab.com/3d-models/crocodylus-siamensis-skull-126fe65e526041ef8815b0f39186c5af

https://sketchfab.com/3d-models/crocodylus-siamensis-mandible-740d91fe790d4425bcdd39ed618154bd

- *Crocodylus thorbjarnarsoni* KNM-ER 1683:

  https://africanfossils.org/fauna/knmer-1683

- *Mecistops cataphractus* TMM M-3529:

  https://www.morphosource.org/concern/media/000114916?locale=en

- *Osteolaemus tetraspis* FMNH 98396:

  https://www.morphosource.org/concern/media/000114918?locale=en

- *Paleosuchus palpebrosus* RVC-JRH-PP1:

  https://sketchfab.com/3d-models/palaeosuchus-palpebrosus-hatchling-crocodylian-13321034846c475e9484f576cdde9e61

  https://osf.io/uad97/

  Hutchinson (2020, March 23)

- *Paleosuchus trigonatus* USNM: amphibians & reptiles: 300660:

  https://www.morphosource.org/Detail/SpecimenDetail/Show/specimen_id/3737

- *Tomistoma schlegelii* TMM M-6342:

  http://digimorph.org/specimens/tomistoma_schlegelii/

CHARACTER LIST AND TAXON MATRIX – MODIFICATIONS AND UPDATES

The taxon matrix and character list from this study represent slightly updated and expanded versions of those published by Ristevski *et al.* (2020a, b), which are based on the datasets by Narváez *et al.* (2015, 2016) and Mateus *et al.* (2019). The dataset used herein is largely the same as the one published by Ristevski *et al.* (2020a, b); the main differences are the additions of the OTU *Gunggamarandu maunala* and one new morphological character (character 229). Additionally, character 149 was slightly modified. More details on the original datasets can be found in Ristevski *et al.* (2020b).

The scores for *Eocaiman cavernensis* were updated according to Godoy *et al.* (2021). Also, the scorings for three characters regarding the OTU *Deinosuchus riograndensis* were updated thanks to information in the recently published study by Cossette & Brochu (2020). These three characters were left as unknowns ('?') in the matrices of Massonne *et al.* (2019) and Ristevski *et al.* (2020a, b), since the study by Cossette & Brochu (2020) was not published by the time of completion of the two aforementioned papers. The amended characters are:

Character 189: *Deinosuchus riograndensis* was scored as an unknown ('?') for this character. Based on the description by Cossette & Brochu (2020), *D. riograndensis* was rescored with state 3 (the 3rd and 4th premaxillary alveoli are largest).

Character 193: *Deinosuchus riograndensis* used to have an unknown ('?') score applied for this character. Based on figure 12A of Cossette & Brochu (2020), state 1 was applied (nasal bone does not reach the height of the orbit).

Character 196: Previously, *Deinosuchus riograndensis* was scored as an unknown ('?') for this character (character 197 of Massonne *et al.*, 2019). Based on figure 15A of Cossette & Brochu

(2020) it is clear that state 0 (sutural contact of the otoccipitals dorsal to foramen magnum long, at least half the height of the foramen magnum) is applicable to this taxon.

Lastly, the scores for character 226 were updated for *Caiman latirostris*, *Paleosuchus palpebrosus* and *Paleosuchus trigonatus*. In the matrix of Ristevski *et al.* (2020a, b), these three caimanines were scored with state 0 for this character (alveolar processes of premaxillae at the first two alveoli are straight). However, some *Caiman latirostris*, *Paleosuchus palpebrosus* and *Paleosuchus trigonatus* specimens display subtle arching at the anterior of the premaxillae, thus necessitating an amendment of their scores from 0 to {0, 1}, addressing both the straight as well as subtly arched anterior premaxillary alveolar processes (e.g., see the 3D digital models in Sookias, 2019).

MORPHOLOGICAL CHARACTER LIST

The following morphological character list is the same as the one used by Ristevski *et al.* (2020a, b), with the only differences being the modification of character 149 and the addition of a newly formulated character, character 229. An explanation on how to score character 229 is provided below. The original source(s) for each character is cited in brackets. For more details on the character list, see Ristevski *et al.* (2020b).

1. Ventral tubercle of proatlas more than one-half (0) or no more than one-half (1) the width of the dorsal crest. (*Brochu, 1997b, ch. 1*)

2. Fused proatlas boomerang-shaped (0), strap-shaped (1), or massive and block-shaped (2). (*Brochu, 1997b, ch. 2*)

3. Proatlas with prominent anterior process (0) or lacks anterior process (1). (*Brochu, 1997b, ch. 10*)

4. Proatlas has tall dorsal keel (0) or lacks tall dorsal keel; dorsal side smooth (1). (*Brochu, 1997b, ch. 17*)

5. Atlas intercentrum wedge-shaped in lateral view, with insignificant parapophyseal processes (0) or plate-shaped in lateral view, with prominent parapophyseal processes at maturity (1). (*Brochu, 1997b, ch. 5; Clark, 1994, ch. 89, modified*)

6. Dorsal margin of atlantal rib generally smooth with modest dorsal process (0) or with prominent process (1). (*Brochu, 1997b, ch. 14*)

7. Atlantal ribs without (0) or with (1) very thin medial laminae at anterior end. (*Brochu, 1997b, ch. 16*)

8. Atlantal ribs lack (0) or possess (1) large articular facets at anterior ends for each other. (*Brochu, 1997b, ch. 15*)

9. Axial rib tuberculum wide, with broad dorsal tip (0) or narrow, with acute dorsal tip (1). (*Brochu, 1997b, ch. 20*)

10. Axial rib tuberculum contacts diapophysis late in ontogeny, if at all (0) or early in ontogeny (1). (*Brochu, 1997b, ch. 21*)

11. Anterior half of axis neural spine oriented horizontally (0) or slopes anteriorly (1). (*Brochu, 1997b, ch. 11*)

12. Axis neural spine crested (0) or not crested (1). (*Brochu, 1997b, ch. 12*)

13. Posterior half of axis neural spine wide (0) or narrow (1). (*Brochu, 1997b, ch. 3*)

14. Axis neural arch lacks (0) or possesses (1) a lateral process ("diapophysis"). (*Brochu, 1997b, ch. 4; adapted from Norell, 1989, ch. 7*)

15. Axial hypapophysis located toward the center of centrum (0) or toward the anterior end of centrum (1). (*Brochu, 1997b, ch. 6*)

16. Axial hypapophysis without (0) or with (1) deep fork. (*Brochu, 1997b, ch. 19*)

17. Hypapophyseal keels present on 11th vertebra behind atlas (0), 12th vertebra behind atlas (1), or 10th vertebra behind atlas (2). (*Brochu, 1997b, ch. 7, modified*)

18. Third cervical vertebra (first postaxial) with prominent hypapophysis (0) or lacks prominent hypapophysis (1). (*Brochu, 1997b, ch. 8; adapted from Norell, 1989, ch. 12; Norell & Clark, 1990, ch. 11; Clark, 1994, ch. 91*)

19. Neural spine on third cervical long, dorsal tip at least half the length of the centrum without the cotyle (0) or short, dorsal tip acute and less than half the length of the centrum without the cotyle (1). (*Brochu, 1997b, ch. 9, modified*)

20. Cervical and anterior dorsal centra lack (0) or bear (1) deep pits on the ventral surface of the centrum. (*Brochu & Storrs, 2012, ch. 20*)

21. Presacral centra amphicoelous (0), or weakly procoelous (1), or strongly procoelous (2). **ORDERED**. (*Salisbury* et al., *2006, ch. 18, modified; adapted from several previous datasets, e.g., Benton & Clark, 1988; Norell & Clark, 1990, ch. 8 and 10 modified; Clark, 1994, ch. 92 and 93; Brochu, 1997b, ch. 18*)

22. Anterior sacral rib capitulum projects far anteriorly of tuberculum and is broadly visible in dorsal view (0) or anterior margins of tuberculum and capitulum nearly in same plane, and capitulum largely obscured dorsally (1). (*Brochu, 1997b, ch. 13*)

23. Scapular blade flares dorsally at maturity (0) or sides of scapular blade subparallel; minimal dorsal flare at maturity (1). (*Brochu, 1997b, ch. 22; adapted from Benton & Clark, 1988*)

24. Deltoid crest of scapula very thin at maturity, with sharp margin (0) or very wide at maturity, with broad margin (1). (*Brochu, 1997b, ch. 23*)

25. Scapulocoracoid synchondrosis closes very late in ontogeny (0) or relatively early in ontogeny (1). (*Brochu, 1997b, ch. 24*)

26. Scapulocoracoid facet anterior to glenoid fossa uniformly narrow (0) or broad immediately anterior to glenoid fossa and tapering anteriorly (1). (*Brochu, 1997b, ch. 25*)

27. Proximal edge of deltopectoral crest emerges smoothly from proximal end of humerus and is not obviously concave (0) or emerges abruptly from proximal end of humerus and is obviously concave (1). (*Brochu, 1997b, ch. 26*)

28. M. teres major and m. dorsalis scapulae insert separately on humerus; scars can be distinguished dorsal to deltopectoral crest (0) or insert with common tendon; single insertion scar (1). (*Brochu, 1997b, ch. 29*)

29. Olecranon process of ulna narrow and sub-angular (0) or wide and rounded (1). (*Brochu, 1997b, ch. 27*)

30. Distal extremity of ulna expanded transversely with respect to long axis of bone; maximum width equivalent to that of proximal extremity (0) or proximal extremity considerably wider than distal extremity (1). (*Salisbury* et al.*, 2006, ch. 173*)

31. Interclavicle flat along length, without dorsoventral flexure (0), or with moderate dorsoventral flexure (1), or with severe dorsoventral flexure (2). (*Brochu, 1997b, ch. 30*)

32. Anterior end of interclavicle flat (0) or rod-like (1). (*Brochu, 1997b, ch. 31*)

33. Pre-acetabular process of ilium present as a prominent triangular process (0) or present only as a barely perceptible bump to absent altogether (1). (*Lee & Yates, 2018, ch. 256; based on Brochu, 1997b, ch. 34)*

34. Dorsal margin of iliac blade rounded with smooth border (0), or rounded, with modest dorsal indentation (1), or rounded, with strong dorsal indentation (‘wasp-waisted’; 2), or narrow, with dorsal indentation (3), or rounded with smooth border; posterior tip of blade very deep (4). (*Brochu, 1997b, ch. 28*)

35. Supraacetabular crest narrow (0) or broad (1). (*Brochu, 1997b, ch. 32*)

36. Limb bones relatively robust, and hind limb much longer than forelimb at maturity (0) or limb bones very long and slender (1). (*Brochu, 1997b, ch. 33, modified*)

37. M. caudofemoralis with single head (0) or with double head (m. caudofemoralis longus and m. caudofemoralis brevis; 1). (*Salisbury* et al.*, 2006, ch. 160*; *modified from Brochu, 1997b, ch. 160*)

38. Dorsal osteoderms keeled (0) or not keeled (1). (*Salisbury* et al.*, 2006, ch. 35; polarity reversed from the version in Brochu, 1997b, ch. 35; adapted from Buscalioni* et al.*, 1992, ch. 22*)

39. Biserial dorsal shield, dorsal osteoderms rectangular in outline with distinct medial and lateral parts either side of a sagittal keel (0), or dorsal osteoderms segmented sagittally into rectangular paravertebral osteoderms and square to round accessory osteoderms (1), or paravertebral osteoderms segmented (2). **ORDERED**. (*Salisbury* et al.*, 2006, ch. 36; adapted from Brochu, 1997b, ch. 36; Norell & Clark, 1990, ch. 16; Clark, 1994, ch. 95*)

40. Accessory osteoderms absent (0), or maximum of one longitudinal row of transversely contiguous accessory osteoderms (1), or maximum of two longitudinal rows of transversely contiguous accessory osteoderms (2), or maximum of three sagittal longitudinal rows of transversely contiguous accessory osteoderms (3). (*Salisbury* et al., *2006, ch. 37; adapted from Brochu, 1997b, ch. 37; Norell & Clark, 1990, ch. 12; Clark, 1994, ch. 97*)

41. Nuchal shield grades continuously into dorsal shield (0), or nuchal shield differentiated from dorsal shield into four nuchal osteoderms in two parallel rows (1), or nuchal shield differentiated from dorsal shield into six nuchal osteoderms, with four central and two lateral (2), or nuchal shield differentiated from dorsal shield into more than four nuchal osteoderms in two parallel rows (3). (*Salisbury* et al., *2006, ch. 38; modified from Brochu, 1997b, ch. 38*)

42. Ventral osteoderms present, polygonal (0), or present, square (1), or present, paired ossifications that suture together (2), or absent (3). (*Salisbury* et al., *2006, ch. 39; Brochu, 1997b, ch. 39 modified; adapted from Buscalioni* et al., *1992, ch. 21; Clark, 1994, ch. 100*)

43. Anterior margin of dorsal midline osteoderms with anterior process (0) or smooth, without process (1). (*Brochu, 1997b, ch. 40; adapted from Norell & Clark, 1990, ch. 13; Clark, 1994, ch. 96*)

44. Ventral scales have (0) or lack (1) follicle gland pores. (*Brochu, 1997b, ch. 155*)

45. Ventral collar scales not enlarged relative to other ventral scales (0), or in a single enlarged row (1), or in two parallel enlarged rows (2). (*Brochu, 1997b, ch. 156; Poe, 1996*)

46. Median pelvic keel scales form two parallel rows along most of tail length (0), or form single row along tail (1), or merge with lateral keel scales (2). (*Brochu, 1997b, ch. 157; Poe, 1996*)

47. Alveoli for dentary teeth 3 and 4 nearly same size and confluent (0) or fourth alveolus larger than third, and alveoli are separated (1). (*Brochu, 1997b, ch. 52*)

48. Anterior dentary teeth strongly procumbent (0) or project anterodorsally (1). (*Brochu, 1997b, ch. 53*)

49. Dentary symphysis extends to fourth or fifth alveolus (0), or sixth through eighth alveolus (1), or behind eighth alveolus (2). **ORDERED**. (*Brochu & Storrs, 2012, ch. 49; modified from Brochu, 2004b, ch. 166*)

50. Dentary gently curved (0), deeply curved (1), or linear (2) between fourth and tenth alveoli. **ORDERED**. (*Brochu, 1997b, ch. 68*)

51. Largest dentary alveolus immediately caudal to fourth is (0) 13 or 14, (1) between 11 or 14 and a series behind it, (2) 11 or 12, (3) no differentiation, (4) behind 14, (5) 10. (*Massonne* et al., *2019, ch. 51; modified from Brochu, 2004b, ch. 167*)

52. Splenial with anterior perforation for mandibular ramus of cranial nerve V (0) or lacks anterior perforation for mandibular ramus of cranial nerve V (1). (*Brochu, 1997b, ch. 41; adapted partially from Norell, 1988, ch. 15 and Norell, 1989, ch. 8*)

53. Mandibular ramus of cranial nerve V exits splenial anteriorly only (0), or splenial has singular perforation for mandibular ramus of cranial nerve V posteriorly (1), or splenial has double perforation for mandibular ramus of cranial nerve V posteriorly (2). (*Brochu, 1997b, ch. 42; adapted partially from Norell, 1988, ch. 15 and Norell, 1989, ch. 8*)

54. Splenial participates in mandibular symphysis; splenial symphysis adjacent to no more than five dentary alveoli (0), or splenial excluded from mandibular symphysis; anterior tip of splenial passes ventral to Meckelian groove (1), or splenial excluded from mandibular symphysis; anterior tip of splenial passes dorsal to Meckelian groove (2), or deep splenial symphysis, longer than five dentary alveoli; splenial forms wide 'V' within symphysis (3), or deep splenial symphysis, longer than five dentary alveoli; splenial constricted within symphysis and forms narrow 'V' (4). **ORDERED**. (*Brochu, 1997b, ch. 43; adapted from Clark, 1994, ch. 77*)

55. Coronoid bounds posterior half of *foramen intermandibularis medius* (0), or completely surrounds *foramen intermandibularis medius* at maturity (1), or obliterates *foramen intermandibularis medius* at maturity (2). **ORDERED**. (*Brochu, 1997b, ch. 46; adapted from Norell, 1988, ch. 12*)

56. Superior edge of coronoid slopes strongly anteriorly (0) or almost horizontal (1). (*Brochu, 1997b, ch. 54*)

57. Inferior process of coronoid laps strongly over inner surface of Meckelian fossa (0) or remains largely on medial surface of mandible (1). (*Brochu, 1997b, ch. 55*)

58. Coronoid imperforate (0) or with perforation posterior to *foramen intermandibularis medius* (1). (*Brochu, 1997b, ch. 56*)

59. Process of splenial separates angular and coronoid (0) or no splenial process between angular and coronoid (1). (*Brochu, 1997b, ch. 59*)

60. Angular-surangular suture contacts external mandibular fenestra at posterior angle at maturity (0), or passes broadly along ventral margin of external mandibular fenestra late in ontogeny (1), or mandibular fenestra between dentary and angular, no surangular participation on the fenestra (2). (*Mateus* et al., *2019, ch. 60*; *Brochu, 1997b, ch. 47, modified; adapted from Norell, 1988, ch. 40*)

61. Dorsal and ventral anterior processes of surangular unequal (0) or sub-equal to equal (1). (*Brochu, 1997b, ch. 48, modified*)

62. Surangular with spur bordering the dentary toothrow lingually for at least one alveolus length (0) or lacking such spur (1). (*Brochu, 1997b, ch. 61*)

63. External mandibular fenestra absent (0), or present as narrow slit, no discrete fenestral concavity on angular dorsal margin (1), or present with discrete concavity on angular dorsal margin (2), or present and very large; most of *foramen intermandibularis caudalis* visible in lateral view (3). (*Brochu, 1997b, ch. 62 and 64, modified; adapted from Clark, 1994, ch. 75*; *includes information from Norell, 1988, ch. 14*)

64. Surangular-dentary suture intersects external mandibular fenestra anterior to posterodorsal corner (0) or at posterodorsal corner (1). (*Brochu, 1997b, ch. 65*)

65. Angular extends dorsally toward or beyond anterior end of *foramen intermandibularis caudalis*; anterior tip acute (0) or does not extend dorsally beyond anterior end of *foramen intermandibularis caudalis*; anterior tip very blunt (1). (*Brochu, 1997b, ch. 66*)

66. Surangular-angular suture lingually meets articular at ventral tip (0) or dorsal to tip (1). (*Brochu, 1997b, ch. 67, modified*)

67. Surangular continues to dorsal tip of lateral wall of glenoid fossa (0) or truncated and not continuing dorsally (1). (*Brochu, 1999, ch. 106*)

68. Articular-surangular suture simple (0), or articular bears anterior lamina dorsal to lingual foramen (1), or articular bears anterior lamina ventral to lingual foramen (2), or bears laminae above and below foramen (3). (*Brochu, 1997b, ch. 44, modified*)

69. Lingual foramen for articular artery and alveolar nerve perforates surangular entirely (0) or perforates surangular/angular suture (1). (*Brochu, 1997b, ch. 45, modified*)

70. Foramen aëreum at extreme lingual margin of retroarticular process (0) or set in from margin of retroarticular process (1). (*Brochu, 1997b, ch. 49; adapted from Norell, 1988, ch. 16*)

71. Retroarticular process projects posteriorly (0) or projects posterodorsally (1). (*Brochu, 1997b, ch. 50; adapted from Benton & Clark, 1988; Norell & Clark, 1990, ch. 7; Clark, 1994 ch. 71*)

72. Surangular extends to posterior end of retroarticular process (0) or pinched off anterior to tip of retroarticular process (1). (*Brochu, 1997b, ch. 51; adapted from Norell, 1988, ch. 42*)

73. Surangular-articular suture oriented anteroposteriorly (0) or bowed strongly laterally (1) within glenoid fossa. (*Brochu, 1997b, ch. 162*)

74. Sulcus between articular and surangular (0) or articular flush against surangular (1). (*Brochu, 1997b, ch. 60*)

75. Dorsal projection of hyoid cornu flat (0) or rod-like (1). (*Brochu, 1997b, ch. 57*)

76. Dorsal projection of hyoid cornu narrow, with parallel sides (0) or flared (1). (*Brochu, 1997b, ch. 58*)

77. Lingual osmoregulatory pores small (0) or large (1). (*Brochu, 1997b, ch. 158*)

78. Tongue with (0) or without (1) keratinized surface. (*Brochu, 1997b, ch. 159*)

79. Teeth and alveoli of maxilla and/or dentary circular to subcircular in cross-section (0), or posterior teeth labiolingually compressed (1), or all teeth labiolingually compressed (2). (*Adapted from Brochu, 2004b, ch. 165*)

80. Maxillary and dentary teeth with smooth carinae (0) or serrated (1). (*Brochu & Storrs, 2012, ch. 80*)

81. External naris is oriented anterodorsally (0) or dorsally (1). (*Brochu, 1997b, ch. 79, modified*)

82. External naris bisected by nasals (0), or nasals contact external naris, but do not bisect it (1), or nasals excluded, at least externally, from naris; nasals and premaxillae still in contact (2), or nasals and premaxillae not in contact (3). **ORDERED**. (*Brochu, 1997b, ch. 95; adapted from Norell, 1988, ch. 3; Clark, 1994, ch. 13 and 14*)

83. Naris circular or keyhole-shaped (0), or wider than long (1), or anteroposteriorly long and prominently teardrop-shaped (2). **ORDERED**. (*Brochu, 1997b, ch. 161, modified*)

84. External naris of reproductively mature males (0) remains similar to that of females or (1) develops bony excrescence (ghara). (*Brochu & Storrs, 2012, ch. 84*)

85. External naris (0) opens flush with dorsal surface of premaxillae or (1) circumscribed by thin crest. (*Brochu & Storrs, 2012, ch. 85*)

86. Premaxillary surface lateral to naris smooth (0) or with deep notch lateral to naris (1). (*Brochu, 1997b, ch. 142*)

87. Premaxilla has five teeth (0) or four teeth (1) early in post-hatching ontogeny. (*Brochu, 1997b, ch. 97; adapted from Norell, 1988, ch. 17*)

88. Incisive foramen absent or small, less than half the greatest width of premaxillae (0), or large, more than half the greatest width of premaxillae (1), or large and intersects premaxillary-maxillary suture (2). (*Brochu, 1997b, ch. 124, modified*)

89. Incisive foramen completely situated far from premaxillary tooth row, at the level of the second or third alveolus, or posterior (0), or abuts premaxillary tooth row (1), or projects between first premaxillary teeth (2). **ORDERED**. (*Brochu, 1997b, ch. 153, modified*)

90. Dorsal premaxillary processes short, not extending beyond third maxillary alveolus (0) or long, extending beyond third maxillary alveolus (1). (*Brochu, 1997b, ch. 145*)

91. Dentary tooth 4 occludes in notch between premaxilla and maxilla early in ontogeny (0) or occludes in a pit between premaxilla and maxilla; no notch early in ontogeny (1). (*Brochu, 1997b, ch. 77; adapted from Norell, 1988, ch. 29*)

92. Occlusion of dentary teeth on the maxillae, with dentary teeth occluding lingual to maxillary teeth with no maxillary reception pits (0), partially interlocking dentition with one or two interdental reception pits between maxillary teeth seven through to nine (1), or fully

interlocking dentition with interdental reception pits from maxillary tooth one to at least tooth nine (2). (*Lee & Yates, 2018, ch. 27*; *adapted from Norell, 1988, ch. 5; Willis, 1993, ch. 1*)

93. Largest maxillary alveolus is 3 (0), 5 (1), 4 (2), 4 and 5 are same size (3), 6 (4), or maxillary teeth homodont (5), or maxillary alveoli gradually increase in diameter posteriorly toward penultimate alveolus (6). (*Brochu & Storrs, 2012, ch. 93; adapted from Norell, 1988, ch. 1*)

94. Maxillary tooth row curved medially or linear (0) or curves laterally broadly (1) posterior to first six maxillary alveoli. (*Brochu, 1997b, ch. 135; adapted from Clark, 1994, ch. 79*)

95. Dorsal surface of rostrum curves smoothly (0) or bears medial dorsal boss (1). (*Brochu, 1997b, ch. 101*)

96. Canthi rostralii absent or very modest (0) or very prominent (1) at maturity. (*Brochu, 1997b, ch. 143; adapted from Norell, 1988, ch. 34*)

97. Preorbital ridges absent or very modest (0) or very prominent (1) at maturity. (*Brochu, 1997b, ch. 144*)

98. Antorbital fenestra present (0) or absent (1). (*Norell & Clark, 1990, ch. 2; Benton & Clark, 1988*)

99. Vomer entirely obscured by premaxilla and maxilla (0) or exposed on palate at premaxillary-maxillary suture (1). (*Brochu, 1997b, ch. 125; adapted from Norell, 1988, ch. 22*)

100. Vomer entirely obscured by maxillae and palatines (0) or exposed on palate between palatines (1). (*Brochu, 1997b, ch. 126*)

101. Surface of maxilla within narial canal imperforate (0) or with a linear array of pits (1). (*Brochu, 1997b, ch. 148, modified*)

102. Medial jugal foramen small (0) or very large (1). (*Brochu, 1997b, ch. 120*)

103. Maxillary foramen for palatine ramus of cranial nerve V small or not present (0) or very large (1). (*Brochu, 1997b, ch. 111*)

104. Ectopterygoid abuts maxillary tooth row (0) or maxilla broadly separates ectopterygoid from maxillary tooth row (1). (*Brochu, 1997b, ch. 91; adapted from Norell, 1988, ch. 19*)

105. Maxilla terminates in palatal view anterior to lower temporal bar (0) or comprises part of the lower temporal bar (1). (*Brochu & Storrs, 2012, ch. 105*)

106. Penultimate maxillary alveolus less than (0) or more than (1) twice the diameter of the last maxillary alveolus. (*Brochu & Storrs, 2012, ch. 106*)

107. Prefrontal dorsal surface smooth adjacent to orbital rim (0) or bearing discrete knob-like processes (1). (*Brochu & Storrs, 2012, ch. 107*)

108. Dorsal half of prefrontal pillar narrow (0) or expanded anteroposteriorly (1). (*Brochu, 1997b, ch. 137; adapted from Norell, 1988, ch. 41*)

109. Medial process of prefrontal pillar expanded dorsoventrally (0) or anteroposteriorly (1). (*Brochu, 1997b, ch. 136*)

110. Prefrontal pillar solid (0) or with large pneumatic recess (1). (*State 1 refers to the prefrontal recess* sensu *Witmer, 1997*; *Brochu, 1999, ch. 99, modified*)

111. Medial process of prefrontal pillar wide (0) or constricted (1) at base. (*Brochu, 1997b, ch. 138*)

112. Shape of the lateral margin of the suborbital fenestra is straight to slightly curved laterally (0) or bowed medially (1). (*Lee & Yates, 2018, ch. 147; Brochu, 1997b, ch. 105, modified*)

113. Anterior face of palatine process rounded or pointed anteriorly (0) or notched anteriorly (1). (*Brochu, 1997b, ch. 108, modified*)

114. Anterior ectopterygoid process tapers to a point (0) or forked (1). (*Brochu, 1997b, ch. 109*)

115. Palatine process extends (0) or does not extend (1) significantly beyond anterior end of suborbital fenestra. (*Brochu, 1997b, ch. 110; adapted from Willis, 1993, ch. 2*)

116. Palatine process generally broad anteriorly (0) or in form of thin wedge (1). (*Brochu, 1997b, ch. 118*)

117. Lateral edges of palatines smooth anteriorly (0) or with lateral process projecting from palatines into suborbital fenestrae (1). (*Brochu, 1997b, ch. 94*)

118. Palatine-pterygoid suture terminates at the posterior limit of the suborbital fenestra (0), or suture lies a short anterior distance up the interfenestral strut so that the pterygoids form a short (less than 20%) section of it (1), or suture lies far anterior up the interfenestral strut so that the pterygoids contribute to at least 20% of the length of the strut (2). **ORDERED**. (*Lee & Yates, 2018, ch. 142, modified; adapted from Brochu, 1997b, ch. 85; the version of this character as originally*

presented by Lee & Yates [2018] had an additional state [“suture terminates medial to the posterior limit of the suborbital fenestra but not appreciably anterior to it”] which is herein removed due to its redundancy with state 1 [=state 2 of ch. 142 in Lee & Yates, 2018])

119. Pterygoid ramus of ectopterygoid straight, posterolateral margin of suborbital fenestra linear (0) or ramus bowed, posterolateral margin of fenestra concave (1). (*Brochu & Storrs, 2012, ch. 119*)

120. Lateral edges of palatines parallel posteriorly (0) or flare posteriorly, producing a ‘shelf’ (1). (*Brochu, 1997b, ch. 90; adapted from Norell, 1988, ch. 2*)

121. Anterior margin of the secondary choana is comprised of the palatines (0), or choana entirely surrounded by pterygoids (1). (*Brochu, 1997b, ch. 71 modified; adapted from Benton & Clark, 1988; Clark, 1994, ch. 43; Norell & Clark, 1990, ch. 1*)

122. Secondary choana projects posteroventrally (0) or anteroventrally (1) at maturity. (*Brochu, 1997b, ch. 72*)

123. Pterygoid surface lateral and anterior to secondary choana flush with choanal margin (0), or pushed inward anterolateral to choanal aperture (1), or pushed inward around choana to form neck surrounding aperture (2). (*Brochu, 1997b, ch. 73, modified*)

124. Posterior rim of secondary choana not deeply notched (0) or deeply notched (1). (*Brochu, 1997b, ch. 107*)

125. Secondary choana not septate (0), or with septum that remains recessed within choana (1), or with septum that projects out of choana (2). **ORDERED**. (*Brochu, 1997b, ch. 152*)

126. Ectopterygoid-pterygoid flexure disappears during ontogeny (0) or remains throughout ontogeny (1). (*Brochu, 1997b, ch. 116*)

127. Ectopterygoid extends (0) or does not extend (1) to posterior tip of lateral pterygoid flange at maturity. (*Brochu, 1997b, ch. 149; adapted from Norell, 1988, ch. 32*)

128. Lacrimal makes broad contact with nasal; no posterior process of maxilla (0), or maxilla with posterior process within lacrimal (1), or maxilla with posterior process between lacrimal and prefrontal (2). (*Brochu, 1997b, ch. 93*)

129. Prefrontals separated by frontals and nasals (0) or prefrontals meet medially (1). (*Norell, 1988, ch. 27*)

130. Lacrimal longer than prefrontal (0), or prefrontal longer than lacrimal (1), or lacrimal and prefrontal both elongate and nearly the same length (2). (*Brochu, 1997b, ch. 117; Norell, 1988, ch. 7, modified*)

131. Anterior tip of frontal (0) forms simple acute point or (1) forms broad, complex sutural contact with the nasals. (*Brochu & Storrs, 2012, ch. 131*)

132. Ectopterygoid extends along medial face of postorbital bar (0) or stops abruptly ventral to postorbital bar (1). (*Brochu, 1997b, ch. 133*)

133. Postorbital bar massive (0) or slender (1). (*Brochu, 1997b, ch. 70; adapted from Norell, 1989, ch. 3*)

134. Postorbital bar bears process that is prominent, dorsoventrally broad, and divisible into two spines (0) or bears process that is short and generally not prominent (1). (*Brochu & Storrs, 2012, ch. 134; Brochu, 1997b, ch. 134, modified; adapted from Norell, 1989, ch. 2*)

135. Ventral margin of postorbital bar flush with lateral jugal surface (0) or inset from lateral jugal surface (1). (*Brochu, 1997b, ch. 146; adapted from Benton & Clark, 1988; Norell & Clark, 1990, ch. 3*)

136. Postorbital bar continuous with anterolateral edge of cranial table (0) or inset (1). (*Salisbury* et al.*, 2006, ch. 175, modified; adapted from Norell & Clark, 1989, ch. 3*)

137. Margin of orbit flush with skull surface (0), or dorsal edges of orbits upturned (1), or orbital margin telescoped (2), or non-telescoped orbits, with frontal having broadly convex orbital margins (3). **ORDERED**. (*Ristevski* et al.*, 2020b, ch. 137; Brochu, 1997b, ch. 103, modified*)

138. Ventral margin of orbit circular (0) or with prominent notch (1). (*Brochu, 1997b, ch. 139*)

139. Palpebral forms from single ossification (0) or from multiple ossifications (1). (*Brochu, 1997b, ch. 96; adapted from Norell, 1988, ch. 8; Clark, 1994, ch. 65*)

140. Quadratojugal spine prominent at maturity (0) or greatly reduced or absent at maturity (1). (*Brochu, 1997b, ch. 69; adapted from Norell, 1989, ch. 1*)

141. Quadratojugal spine low, near posterior angle of infratemporal fenestra (0) or high, between posterior and superior angles of infratemporal fenestra (1). (*Brochu, 1997b, ch. 114*)

142. Quadratojugal forms posterior angle of infratemporal fenestra (0), or jugal forms posterior angle of infratemporal fenestra (1), or quadratojugal-jugal suture lies at posterior angle of infratemporal fenestra (2). **ORDERED**. (*Brochu, 1997b, ch. 75; adapted from Norell, 1989, ch. 10*)

143. Postorbital neither contacts quadrate nor quadratojugal medially (0), or contacts quadratojugal, but not quadrate, medially (1), or contacts quadrate and quadratojugal at dorsal angle of infratemporal fenestra (2). (*Brochu, 1997b, ch. 76, modified*)

144. Quadratojugal bears long anterior process along lower temporal bar (0) or bears modest process, or none at all, along lower temporal bar (1). (*Brochu, 1997b, ch. 83*)

145. Quadratojugal extends to superior angle of infratemporal fenestra (0) or does not extend to superior angle of infratemporal fenestra; quadrate participates in the infratemporal fenestra (1). (*Brochu, 1997b, ch. 80; adapted from Buscalioni* et al.*, 1992, ch. 6*)

146. Postorbital-squamosal suture oriented ventrally (0) or passes medially (1) ventral to cranial table. (*Brochu, 1997b, ch. 163*)

147. Dorsal and ventral rims of squamosal groove for external ear valve musculature parallel (0) or squamosal groove flares anteriorly (1). (*Brochu, 1997b, ch. 84*)

148. Quadrate and squamosal not in contact on the external surface of the skull, posteriorly to the external auditory meatus (0), or quadratosquamosal suture extends dorsally along caudal margin of the external auditory meatus (1) or extends only to the caudoventral corner of the external auditory meatus (2). (*As modified by Delfino* et al.*, 2008a from Brochu, 1997b, ch. 132 and Salisbury* et al.*, 2006, ch. 132*)

149. Caudal margin of meatal chamber smooth and continuous with the paraoccipital process (0), or caudal margin of meatal chamber inset (1). (*Salisbury* et al., *2006, ch. 102 modified*; *adapted from Brochu, 1997b, ch. 102; meatal chamber* sensu *Montefeltro* et al., *2016*)

150. Frontoparietal suture deeply within supratemporal fenestra; frontal prevents broad contact between postorbital and parietal (0), or suture makes modest entry into supratemporal fenestra at maturity; postorbital and parietal in broad contact (1), or suture on cranial table entirely (2). **ORDERED**. (*Brochu, 1997b, ch. 81*)

151. Frontoparietal suture concavo-convex (0) or linear (1) between supratemporal fenestrae. (*Brochu, 1997b, ch. 86*)

152. Supratemporal fenestra with fossa; dermal bones of cranial table do not overhang rim at maturity (0), or dermal bones of cranial table overhang rim of supratemporal fenestra near maturity (1), or supratemporal fenestra closes during ontogeny (2), or dermal bones of cranial table overhang rim of supratemporal fenestra near maturity; fenestrae large, significantly longer than wide, with an oval shape (3). (*Cidade* et al., *2017, ch. 151 modified*; *based on Brochu, 1997b, ch. 87; adapted from Norell, 1988, ch. 9*)

153. Shallow fossa at anteromedial corner of supratemporal fenestra (0) or no such fossa; anteromedial corner of supratemporal fenestra smooth (1). (*Brochu, 1997b, ch. 92*)

154. Medial parietal wall of supratemporal fenestra imperforate (0) or bearing foramina (1). (*Brochu, 1997b, ch. 104; adapted from Norell, 1988, ch. 51*)

155. Parietal and squamosal widely separated by quadrate on posterior wall of supratemporal fenestra (0), or parietal and squamosal approach each other on posterior wall of

supratemporal fenestra without actually making contact (1), or parietal and squamosal meet along posterior wall of supratemporal fenestra (2). (*Brochu, 1997b, ch. 131*)

156. Cranial table surface slopes ventrally from sagittal axis (0) or planar (1) at maturity. (*Brochu, 1997b, ch. 123, modified*)

157. Posterolateral margin of squamosal horizontal or nearly so (0) or upturned to form a discrete 'horn' (1). (*Brochu & Storrs, 2012, ch. 157*)

158. Mature cranial table with broad curvature; short posterolateral squamosal rami along paroccipital process (0) or with nearly horizontal sides; significant posterolateral squamosal rami along paroccipital process (1). (*Brochu, 1997b, ch. 140, modified*)

159. Squamosal does not extend (0) or extends (1) ventrolaterally to lateral extent of paraoccipital process. (*Brochu, 1997b, ch. 150, modified*)

160. Supraoccipital exposure on dorsal cranial table small (0), absent (1), large (2), or large such that parietal is excluded from posterior edge of the cranial table (3). **ORDERED**. (*Brochu, 1997b, ch. 82, modified; adapted from Norell, 1988, ch. 11*)

161. Anterior foramen for palatine ramus of cranial nerve VII ventrolateral (0) or ventral (1) to basisphenoid rostrum. (*Brochu, 1997b, ch. 164*)

162. Sulcus on anterior braincase wall lateral to basisphenoid rostrum (0) or braincase wall lateral to basisphenoid rostrum smooth; no sulcus (1). (*Brochu, 1997b, ch. 122*)

163. Basisphenoid not exposed extensively (0) or exposed extensively (1) on braincase wall anterior to trigeminal foramen. (*Brochu, 1997b, ch. 129, modified; adapted from Norell, 1989, ch. 5*)

164. Extensive exposure of prootic on external braincase wall (0) or prootic largely obscured by quadrate and laterosphenoid externally (1). (*Brochu, 1997b, ch. 74; adapted from Norell, 1989, ch. 5*)

165. Lateral laterosphenoid bridge comprised entirely of laterosphenoid (0) or includes an ascending process of the palatine (1). (*Brochu, 1997b, ch. 115, modified*)

166. Capitate process of laterosphenoid oriented laterally (0) or anteroposteriorly (1) toward midline. (*Brochu, 1997b, ch. 130*)

167. Parietal with recess communicating with pneumatic system (0) or solid, without recess (1). (*Brochu, 1997b, ch. 154, modified*)

168. Significant ventral quadrate process on lateral braincase wall (0) or quadrate-pterygoid suture linear from basisphenoid exposure to trigeminal foramen (1). (*Brochu, 1997b, ch. 127, modified*)

169. Posterior carotid foramen opens lateral (0) or dorsal (1) to basisphenoid at maturity. (*Brochu, 1997b, ch. 128, modified*)

170. External surface of basioccipital ventral to occipital condyle oriented posteroventrally (0) or posteriorly (1) at maturity. (*Brochu & Storrs, 2012, ch. 170; adapted from Hua & Jouve, 2004, ch. 167 and Salisbury* et al.*, 2006, ch. 174*)

171. Posterior pterygoid processes tall and prominent (0), or small and project posteroventrally (1), or small and project posteriorly (2). (*Brochu, 1997b, ch. 98*)

172. Basisphenoid thin (0) or anteroposteriorly wide (1) ventral to basioccipital. (*Brochu, 1997b, ch. 113*)

173. Basisphenoid not broadly exposed ventral to basioccipital at maturity; pterygoid short ventral to median pharyngeal tube foramen (0) or basisphenoid exposed as broad sheet ventral to basioccipital at maturity; pterygoid tall ventral to median pharyngeal tube foramen (1). (*Brochu, 1997b, ch. 119 modified*; *terminology modified in accord with Dufeau & Witmer, 2015 and Young & Bierman, 2019*)

174. Otoccipital with very prominent boss on paroccipital process; process lateral to cranioquadrate opening short (0) or otoccipital with small or no boss on paroccipital process; process lateral to cranioquadrate opening long (1). (*Brochu, 1997b, ch. 141, modified*)

175. Pharyngotympanic foramina open dorsal (0) or lateral (1) to median pharyngeal tube foramen. (*Brochu, 1997b, ch. 147, modified; adapted from Norell, 1988, ch. 46*; *terminology modified in accord with Dufeau & Witmer, 2015 and Young & Bierman, 2019*)

176. Otoccipitals terminate dorsal to basioccipital tubera (0), or send robust process ventrally and participate in basioccipital tubera (1), or send slender process ventrally to basioccipital tubera (2). **ORDERED**. (*Brochu, 1997b, ch. 151, modified; adapted from Norell, 1988, ch. 20; Clark, 1994, ch. 57 and 60*)

177. Quadrate foramen aëreum on mediodorsal angle (0) or on dorsal surface (1) of quadrate. (*Brochu, 1997b, ch. 121*)

178. Quadrate foramen aëreum is small (0), comparatively large (1), or absent (2) at maturity. (*Brochu & Storrs, 2012, ch. 178*)

179. Quadrate lacks (0) or bears (1) prominent, mediolaterally thin crest on dorsal surface of ramus. (*Brochu & Storrs, 2012, ch. 179*)

180. Attachment scar for posterior mandibular adductor muscle on ventral surface of quadrate ramus forms modest crests (0) or prominent knob (1). (*Brochu & Storrs, 2012, ch. 180; adapted from Ősi* et al., *2007, ch. 165*)

181. Quadrate with small, ventrally reflected medial hemicondyle (0), or with small medial hemicondyle; dorsal notch for foramen aëreum (1), or with prominent dorsal projection between hemicondyles (2), or with expanded medial hemicondyle (3). (*Brochu, 1997b, ch. 112*)

182. Iris (0) greenish/yellowish or (1) brown. (*Brochu & Storrs, 2012, ch. 182*)

183. Fewer than eight (0), or eight to 14 (1), or more than 14 (2) paired midline scale rows (*Brochu & Storrs, 2012, ch.184*; *there is considerable variation in this character within species, and further work is needed to clarify the situation; data from Fuchs, 2006*)

184. Ectopterygoid maxillary ramus forms less than (0) or more than (1) two-thirds of lateral margin of suborbital fenestra. (*Brochu & Storrs, 2012, ch. 185*)

185. Ectopterygoid maxillary ramus terminates at lateral margin of suborbital fenestra (0) or lateral to it, with maxilla separating the ectopterygoid from fenestra for short distance (1). (*Brochu & Storrs, 2012, ch. 186*)

186. Palatine-maxillary suture intersects suborbital fenestra at its anteromedial margin (0) or nearly at its anterior-most limit (1). (*Brochu & Storrs, 2012, ch. 187*)

187. Frontal lacks (0) or bears (1) prominent midsagittal crest between orbits. (*Brochu & Storrs, 2012, ch. 188*)

188. All cervical neural spines anteroposteriorly broad (0) or posterior neural spines thin and rod-like (1). (*Brochu & Storrs, 2012, ch. 189; adapted from Clark, 1994, ch. 90, Pol* et al.*, 2009, ch. 90*)

189. Largest premaxillary tooth is the second (0), the third (1), or the fourth (2), or the third and fourth are largest (3), or all similar in size (4), or the fourth and fifth are largest (5), or the first four equal in size (6). (*Massonne* et al.*, 2019, ch. 190; based on Wang* et al.*, 2016, ch. 190*)

190. Dorsal surface of the surangular, lateral to the glenoid fossa is smooth (0), or bears a single pit (1), or bears two pits (2), or bears a large elongated sulcus next to the anterior half of the glenoid fossa (3), or intensely ornamented as the lateral surface of the surangular (4). (*Based on Wang* et al.*, 2016, ch. 191 and Lee & Yates, 2018, ch. 204*)

191. 'U'-shaped depression of the frontal at the point of maximum constriction between the orbits absent (0) or present (1). (*Cossette & Brochu, 2018, ch. 107, modified*)

192. Skull in lateral view relatively flat (0) or wedge-shaped (1). (*Massonne* et al.*, 2019, ch. 193*)

193. Nasal bone does (0) or does not (1) reach to the height of the orbit. (*Massonne* et al.*, 2019, ch. 194*)

194. Anterior process of jugal extends anterior (0), lies at the same level as (1), or well posterior to the anterior process of frontal (2). (*Massonne* et al.*, 2019, ch. 195; modified from Jouve, 2004, ch. 177; Jouve* et al.*, 2008, ch. 174 and Jouve, 2016, ch. 174*)

195. Notch between the premaxilla and maxilla present (0), or absent (1) in adult individuals. (*Massonne* et al.*, 2019, ch. 196*)

196. Sutural contact of the otoccipitals dorsal to foramen magnum (0) long, at least half the height of the foramen magnum, (1) short, shorter than half the height of the foramen magnum, or (2) no sutural contact between the otoccipitals. (*Massonne* et al.*, 2019, ch. 197, modified*)

197. Anterior maxillary teeth without (0) or with (1) ridges on their labial surface. (*Massonne* et al.*, 2019, ch. 198, modified*)

198. If largest dentary alveolus is between 11th and 14th and a series behind it, is it the (0) 11th, (1) 12th, or (2) 13th to 14th. (*Massonne* et al.*, 2019, ch. 200; applicable only to taxa that are scored with state 1 for character 51 [see Massonne* et al.*, 2019]*)

199. Surangular-angular suture lingually originates (0) near the ventral border of the external mandibular fenestra, (1) near the dorsal border of the external mandibular fenestra and straight, (2) near the dorsal border of the external mandibular fenestra and bowed. (*Massonne* et al.*, 2019, ch. 201*)

200. Large or very large supraoccipital exposure on cranial table is (0) trapezoid, (1) triangular, or (2) block-shaped. (*Massonne* et al.*, 2019, ch. 202, modified; applicable only to taxa that have large or very large supraoccipital exposure on cranial table, i.e., applicable to taxa that are scored with either state 2 or 3 for character 160 [see Massonne* et al.*, 2019]*)

201. Anterior profile of the premaxilla in lateral view is shallowly sloping (0) or steeply angled so that it is close to vertical (1). (*Lee & Yates, 2018, ch. 1*)

202. Alveolar process of the maxilla along the first six maxillary alveoli is inconspicuous or low, with medial lamina comprising no more than 10% of total rostral height (0), or moderately developed, with medial lamina comprising between 10–25% of total rostral height (1), or tall, with medial lamina comprising 25% or more of total rostral height (2). **ORDERED**. (*Adapted from Salisbury & Willis, 1996, ch. 16 and Lee & Yates, 2018, ch. 26*)

203. Anterior margins of suborbital fenestrae set to the level of 9th maxillary alveoli or posterior (0), or between the 9th and 7th maxillary alveoli (1), or to the level of 7th maxillary alveoli or anterior (2). (*Modified from Salisbury & Willis, 1996, ch. 14 and Lee & Yates, 2018, ch. 144*)

204. Jugal-lacrimal contact expressed by a long suture that widely separates the maxilla from the orbital margin (0), or point contact that narrowly separates the maxilla from the orbital margin (1), or no jugal-lacrimal contact, with the maxilla contributing to the orbital margin (2). (*Lee & Yates, 2018, ch. 38, modified; based on information in Willis [1997, 2001]*)

205. Orientation of the ventral ornamented portion of the jugal, anterior to the postorbital bar is lateral to ventrolateral (0) or sharply bent to face ventrally (1). (*Lee & Yates, 2018, ch. 62, modified*)

206. Cross-sectional shape of the anterior ramus of the ectopterygoid subtriangular (0) or a dorsoventrally compressed plate projecting medially from the maxilla (1). (*Lee & Yates, 2018, ch. 153, modified*)

207. Outline of supratemporal fenestra is sub-square (0), circular to sub-circular (1) sub-rectangular, longer anteroposteriorly (2), sub-rectangular, longer mediolaterally (3), elliptical, with the long axis aligned parasagittaly (4), or D-shaped (5). (*Reformulated from Lee & Yates, 2018, ch. 75; adapted in part from de Andrade* et al.*, 2011, ch. 111*)

208. Minimum width between supratemporal fenestrae with respect to maximum cranial table width is 10% or less of total width (0), or between 10–20% of total width (1), or at least 20% of total width (2). (*Tennant* et al., *2016, ch. 126, modified; based on information in Schwarz and Salisbury, 2005*)

209. The bar forming the posterior border of the supratemporal fenestra (measured as the minimum distance between the posterior margin of the supratemporal fenestra and the occipital margin of the cranial table) is robust, with the minimum thickness being greater than 18% of the width of the cranial table at the level of the postorbital-squamosal suture (0), or the minimum thickness is 8–18 % of the width of the cranial table (1), or slender, with the minimum thickness being less than 8% of the width of the cranial table (2). (*Adapted from Lee & Yates, 2018, ch. 95*)

210. Anteromedial extension of the frontoparietal fossa onto the dorsal cranial roof not present at any stage of ontogeny (0) or present in all but the most aged adults (1). (*Lee & Yates, 2018, ch. 73, modified; frontoparietal fossa* sensu *Holliday* et al., *2020*)

211. Large sculpture pits on cranial table developed anterior to the supratemporal fenestrae absent (0), or present and spread over the frontal-parietal junction, with minimal or no involvement of the postorbital (1), or present and spread over the postorbital-frontal-parietal triple junction (2). (*Lee & Yates, 2018, ch. 71, modified; based on information in Willis* et al., *1993*)

212. Ventral extent of the dorsal ornamentation into the lateral squamosal attachment area for the upper earlid musculature extends no further than the dorsal margin of the attachment area (0) or rugose ornamentation that extends into the lateral squamosal sulcus so that only the ventrolateral rim remains smooth (1). (*Lee & Yates, 2018, ch. 82, modified*)

213. Angle of posteroventral descent of the subdermal portion of the squamosal at its posterolateral corner is shallow, about 45° or less (0) or steep and close to vertical (1). (*Lee & Yates, 2018, ch. 88*)

214. The quadrate pterygoid process has minimal or no occipital exposure (0) or evident occipital exposure (1) ventrolateral to the otoccipital. (*Lee & Yates, 2018, ch. 116, modified; quadrate pterygoid process* sensu *Kley* et al.*, 2010*)

215. Distance between the posterior tip of the paroccipital process and the quadrate condylar surface is less than the width of the quadrate condylar surface (0) or greater than the width of the quadrate condylar surface (1). (*Lee & Yates, 2018, ch. 111, modified; based on information in Molnar, 1982; distance measured from the posterior tip of the paroccipital process to the medial quadrate hemicondyle*)

216. Quadrate condyles and occipital condyle aligned on the same plane (0) or unaligned, quadrate condyles are at a lower level than the occipital condyle (1). (*Adapted from de Andrade* et al.*, 2011, ch. 4; based on Wu & Sues, 1996, ch. 24*)

217. Anteriorly directed ridges on the pterygoid plates extending from the lateral margins of the secondary choana absent (0) or present (1). (*Lee & Yates, 2018, ch.161*)

218. Shape of the secondary choana is ovoid to cordiform, with the apex directed posteriorly (0), or triangular, with the apex directed anteriorly (1). (*Lee & Yates, 2018, ch. 163; original version of the character from Jouve* et al.*, 2015, ch. 236*)

219. Shape of the ventrolateral margin of the dentary is smoothly rounded (0) or curved at approximately a 90˚ angle, resulting in a horizontal ventral surface and a vertical lateral surface (1). (*Lee & Yates, 2018, ch. 181, modified*)

220. Ventral margin of the ornamented region of the angular simple, not everted (0) or everted into a laterally to ventrolaterally projecting flange (1). (*Lee & Yates, 2018, ch. 199*)

221. Laterally directed flange along the dorsolateral margin of the surangular, bordering the ornamented region is absent (0) or present (1). (*Lee & Yates, 2018, ch. 203; Pol & Norell, 2004, ch. 187, modified*)

222. Maxillary teeth/alveoli size disparity at adulthood low, largest maxillary tooth/alveolus is less than twice the diameter of the smallest interfestoonal tooth/alveolus (0) or high, diameter of the largest tooth/alveolus more than twice that of the smallest interfestoonal tooth/alveolus (1). (*Modified from Salisbury & Willis, 1996, ch. 17 and Lee & Yates, 2018, ch. 220*)

223. Maxillary alveolar count is 11 or fewer (0), 12–16 (1), 17–20 (2), 21–28 (3), 29 or more (4) alveoli. **ORDERED**. (*Young, 2014, ch. 156*)

224. Dentary alveolar count is 14 or fewer (0), 15–19 (1), 20–29 (2), 30 or more (3) alveoli per rami. **ORDERED**. (*Young, 2014, ch. 158, modified*)

225. Adult skull length is 300 mm or more (0) or less than 300 mm (1). (*Lee & Yates, 2018, ch. 219, modified; here, total skull length is measured from the anterior tip of the rostrum to the lateral quadrate hemicondyle*)

226. Alveolar processes of premaxillae at the first two alveoli are straight (0) or arched (1). (*Ristevski* et al.*, 2020b, ch. 226*)

227. Laterally directed flange along the dorsolateral margin of the dentary, labial to the last four to five dentary alveoli is absent (0) or present (1). (*Based on Lee & Yates, 2018, ch. 179*)

228. Occipital lamina of the supraoccipital is oriented posteriorly (0) or posteroventrally (1). (*Ristevski* et al., *2020b, ch. 228*)

229. Postoccipital processes of the supraoccipital are small to moderately large (0), or very large and widely spaced (1), or very large and closely spaced (2). **NEW**

Explanation for scoring character 229

The postoccipital processes of the supraoccipital (*sensu* Kälin, 1933) are knob-like protrusions on the occipital surface of the supraoccipital. The region of the occiput where the postoccipital processes are located serves as an attachment site for the M. transversospinalis capitis muscle (*sensu* Frey, 1988; see also Buttmann, 1826; Hair, 1868; Iordansky, 1973; Bakker *et al.*, 1988; Rossmann, 2000; Salisbury, 2001), with the tendons of said muscle inserting on these processes (Iordansky, 1973). The processes are visible dorsolaterally on the occipital surface of the supraoccipital, bounding the ventral margins of the posttemporal fenestrae. They can have a variable morphology between taxa, ranging from small to moderately pronounced processes that are modestly visible, or not visible at all in dorsal view; this is the most common condition among crocodylians (state 0; **Fig. S2.1A** and **S2.1B**). Otherwise, they can be very prominent in size, evident both in occipital and dorsal views of the skull and spaced widely apart from each other, like in some tomistomines (e.g., *Dollosuchoides densmorei*, *Gunggamarandu maunala*, *Tomistoma schlegelii*; state 1; **Fig. S2.1C** and **S2.1D**). Also, they can be very prominent, yet spaced closely to each other – this condition is present among Gavialidae (state 2; **Fig. S2.1E** and **S2.1F**). The morphology and size of the postoccipital processes relative to the cranium appear to have little to no ontogenetic or individual variation, at least in *Crocodylus johnstoni* and *C. porosus* (Jorgo Ristevski pers. obs. of *C. johnstoni* and *C. porosus* specimens, 2018).

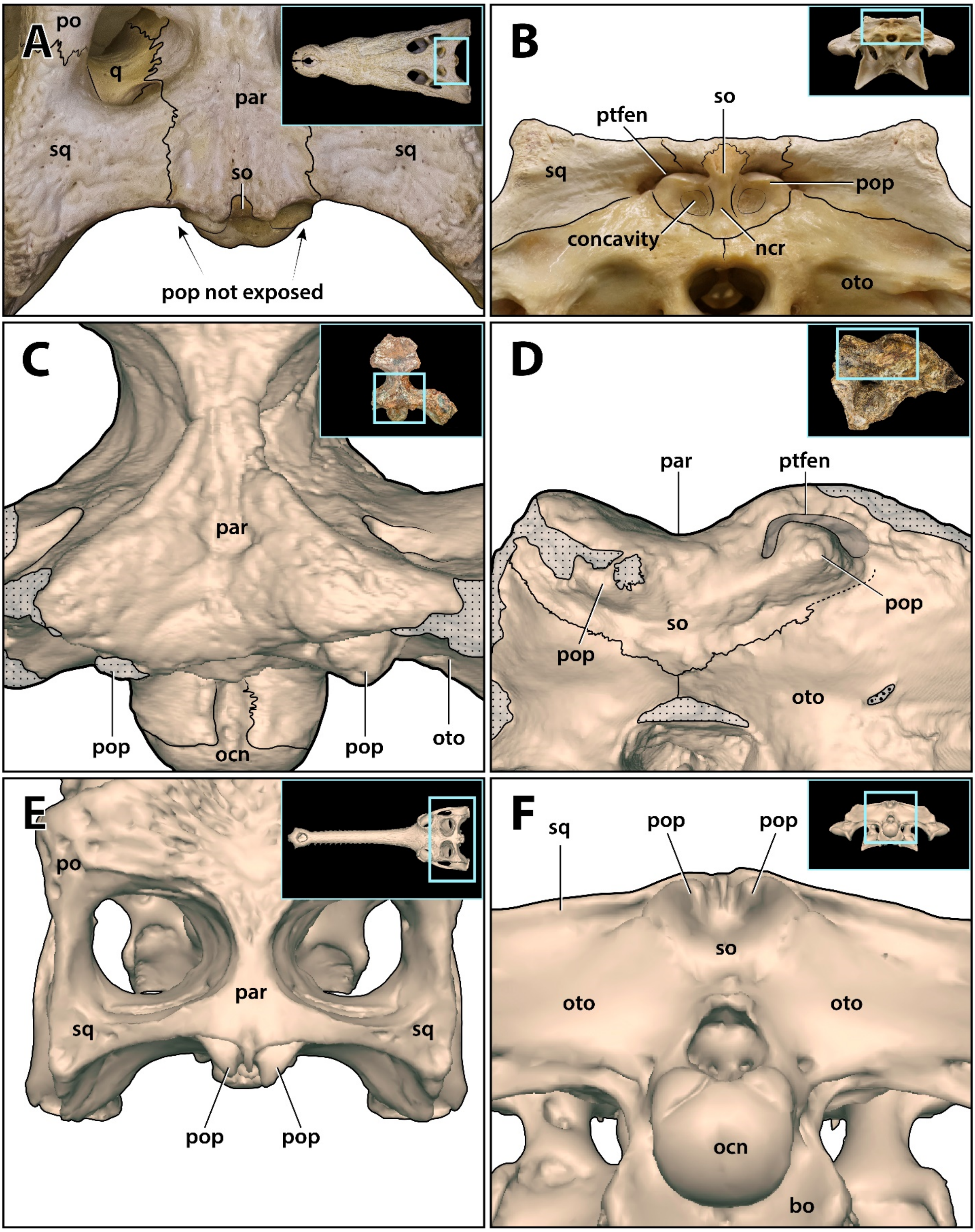
A
po
q
par
sq
sq
so
pop not exposed
B
so
ptfen
sq
pop
concavity
ncr
oto
C
par
pop
ocn
pop
oto
D
par
ptfen
pop
so
pop
oto
E
po
par
sq
sq
pop
pop
F
sq
pop
pop
so
oto
oto
ocn
bo

Figure S2.1 (on previous page) **Explanation for scoring character 229 in the list.** *Crocodylus porosus* Schneider, 1801, QMJ48127, (A) close up of the cranial table in dorsal view, and (B) the skull in occipital view. Notice how in (A) the postoccipital processes of the supraoccipital are not visible in dorsal view. *Gunggamarandu maunala* gen. et sp. nov., QMF14.548, holotype, (C) close up of the cranial table in dorsal view, and (D) skull in occipital view. *Gavialis gangeticus* (Gmelin, 1789), (E) close up of the cranial table in dorsal view, and (F) skull in occipital view. Notice how in (E) and (F) the postoccipital processes of the supraoccipital are acute and closely spaced. The images of the *Gavialis gangeticus* skull in (E) and (F) are from a 3D digital model provided as a supplementary file to Sookias (2020); see Sookias (2019, October 30). The gray, dotted areas in (C) and (D) indicate broken and/or damaged surfaces on the figured specimen. Abbreviations: **bo**, basioccipital; **ncr**, nuchal crest; **ocn**, occipital condyle; **oto**, otoccipital; **par**, parietal; **po**, postorbital; **pop**, postoccipital process of the supraoccipital; **ptfen**, posttemporal fenestra; **q**, quadrate; **so**, supraoccipital; **sq**, squamosal.

PHYLOGENETIC ANALYSES

As mentioned in the "Phylogeny" and "Phylogenetic methods" subsections of the main text, four phylogenetic analyses were performed – one using a 'traditional' equal weighting (EW) principal search approach, while the other three analyses used the implied weighting (IW) methodology. The analyses that utilized the IW methodology were run with three different concavity constant (*k*) values, one set to 3.0, the second set to 12.0, and the last set to 25.0. The search parameters and settings used to run the analyses are described in the "Phylogenetic methods" subsection of the main text, and a brief summary of the results, mainly focusing on the position of *Gunggamarandu maunala*, and their implications are also provided therein. What follows is a more detailed overview of the results from the EW and IW analyses.

Results

Equal Weighting analysis

The phylogenetic analysis performed under an EW search method recovered 339 most-parsimonious cladograms (MPCs) with a length of 1412 steps (CI = 0.228; RI = 0.725). Similar to the EW analysis results of Ristevski *et al.* (2020a, b), the strict consensus topology (**Fig. S2.2**) depicts a large unresolved polytomy within Crocodylia that also incorporates the mekosuchine OTUs. There are two mekosuchine clades recovered from the strict consensus of this analysis, one comprised by the *Baru* Willis *et al.*, 1990 OTUs, and the second by the *Mekosuchus* Balouet & Buffetaut, 1987 OTUs; regardless, both the *Baru* and *Mekosuchus* clades are parts of the polytomy.

The 50% majority-rule consensus topology (**Fig. S2.3**) has a much better resolution, however, the interrelationships of mekosuchines (all found within Crocodyloidea) are still unclear. In the 50% majority-rule consensus, *Paludirex vincenti* is the sister taxon to the *Baru* + *Mekosuchus*

clade. *Australosuchus clarkae*, *Kalthifrons aurivellensis*, *Quinkana timara*, as well as *Harpacochampsa camfieldensis* are independent of the other mekosuchine OTUs, although still sitting in an unresolved position in Crocodyloidea. Likewise, the species of *Kambara* Willis *et al.*, 1993 are also independent of the other mekosuchines, but do form a monophyletic clade, with *K. implexidens* as a sister taxon to *K. murgonensis* + *K. taraina*. Generally, the nodal support is weak (**Figs. S2.4** and **S2.5**).

Intriguingly, one of the few consistently recovered clades in both the strict and 50% majority-rule consensuses is Gavialoidea, comprised of Gavialidae, Tomistominae, and 'thoracosaurs'. The same result was recovered in the EW analysis by Ristevski *et al.* (2020a, b). This is not surprising, since the matrix used in this study is essentially the same as the one published by Ristevski *et al.* (2020a, b), bar the few updates implemented herein. As already discussed by Ristevski *et al.* (2020a), recovering a monophyletic Gavialoidea that incorporates Gavialidae and Tomistominae has been a challenge for many older studies that performed phylogenetic analyses based on morphological data alone. In the strict consensus, the recovered Gavialoidea (that also includes the 'thoracosaurs') lies in a large polytomy within Crocodylia. However, the 50% majority-rule consensus finds Gavialoidea forming a clade with Crocodyloidea (=Longirostres *sensu* Harshman *et al.*, 2003). The gavialid and 'thoracosaur' taxa are recovered in more derived positions within Gavialoidea, whereas the tomistomines occupy the more basal positions. The basal-most taxon in the clade is the tomistomine *Kentisuchus spenceri*. Unfortunately, the nodal support for Gavialoidea is unsatisfactory (Bremer = 0; bootstrap = <50%). *Gunggamarandu maunala* is found near the base of Gavialoidea along with certain other tomistomines, and is also the sister taxon to *Dollosuchoides densmorei*. The *Gunggamarandu* gen. nov. + *Dollosuchoides* Brochu, 2007b clade is one of the stronger supported gavialoid sub-clades recovered from the EW analysis (Bremer = 2; bootstrap = <50%). The *Gunggamarandu* + *Dollosuchoides* clade is characterized by two synapomorphies: a slender postfenestral bar, with a minimum thickness that is less than 8% the width of the cranial table (character 209, state 2); and, very large and widely

spaced postoccipital processes of the supraoccipital (character 229, state 1). No autapomorphies were indicated for *G. maunala* in this analysis.

As in our previous study (Ristevski *et al*., 2020a), we reiterate that our parsimony-based analysis results who yielded a cladogram including a monophyletic Gavialoidea do not solve the longstanding debate on the relationships between *Gavialis* Oppel, 1811, *Tomistoma* Müller, 1846 and their extinct relatives. More work is clearly needed to settle the issues surrounding this subject, which is outside the aims and scope of our work.

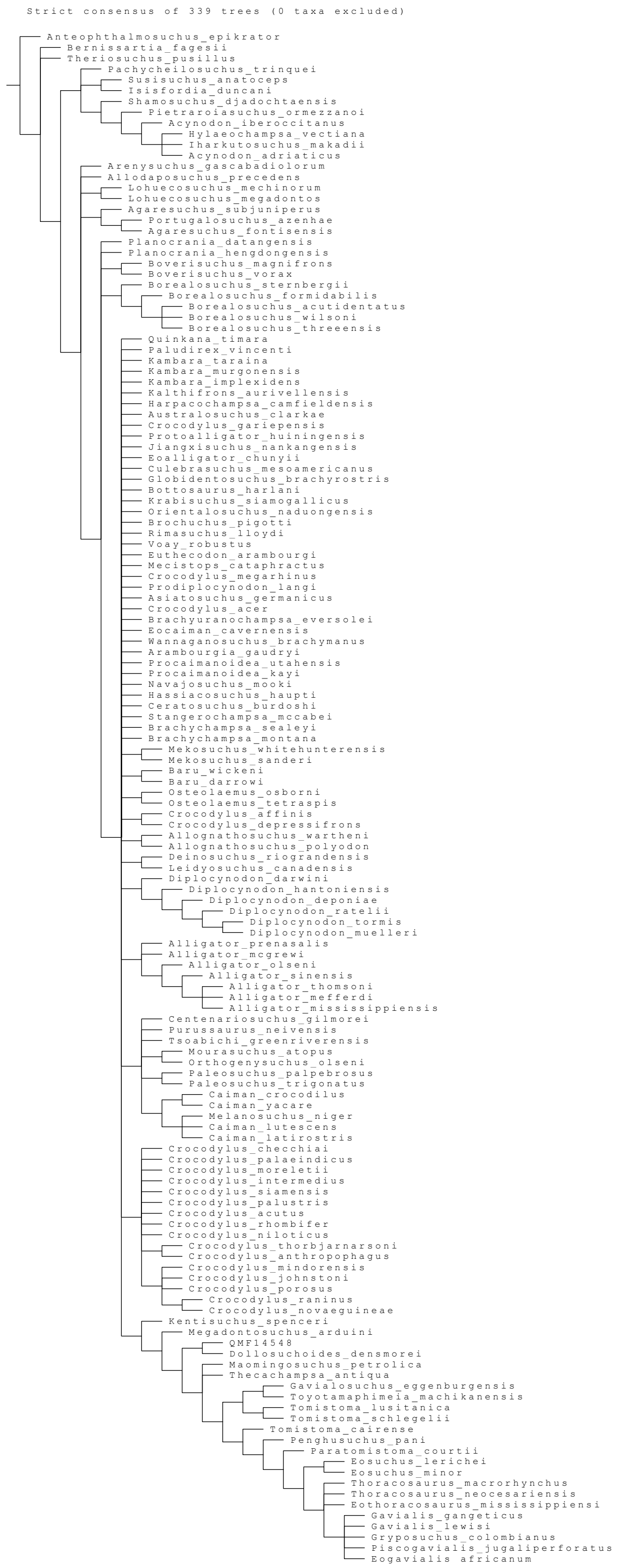

Figure S2.2 Strict consensus topology from the phylogenetic analysis performed under the EW method. In the cladogram, *Gunggamarandu maunala* gen. et sp. nov. is the OTU named QMF14548.

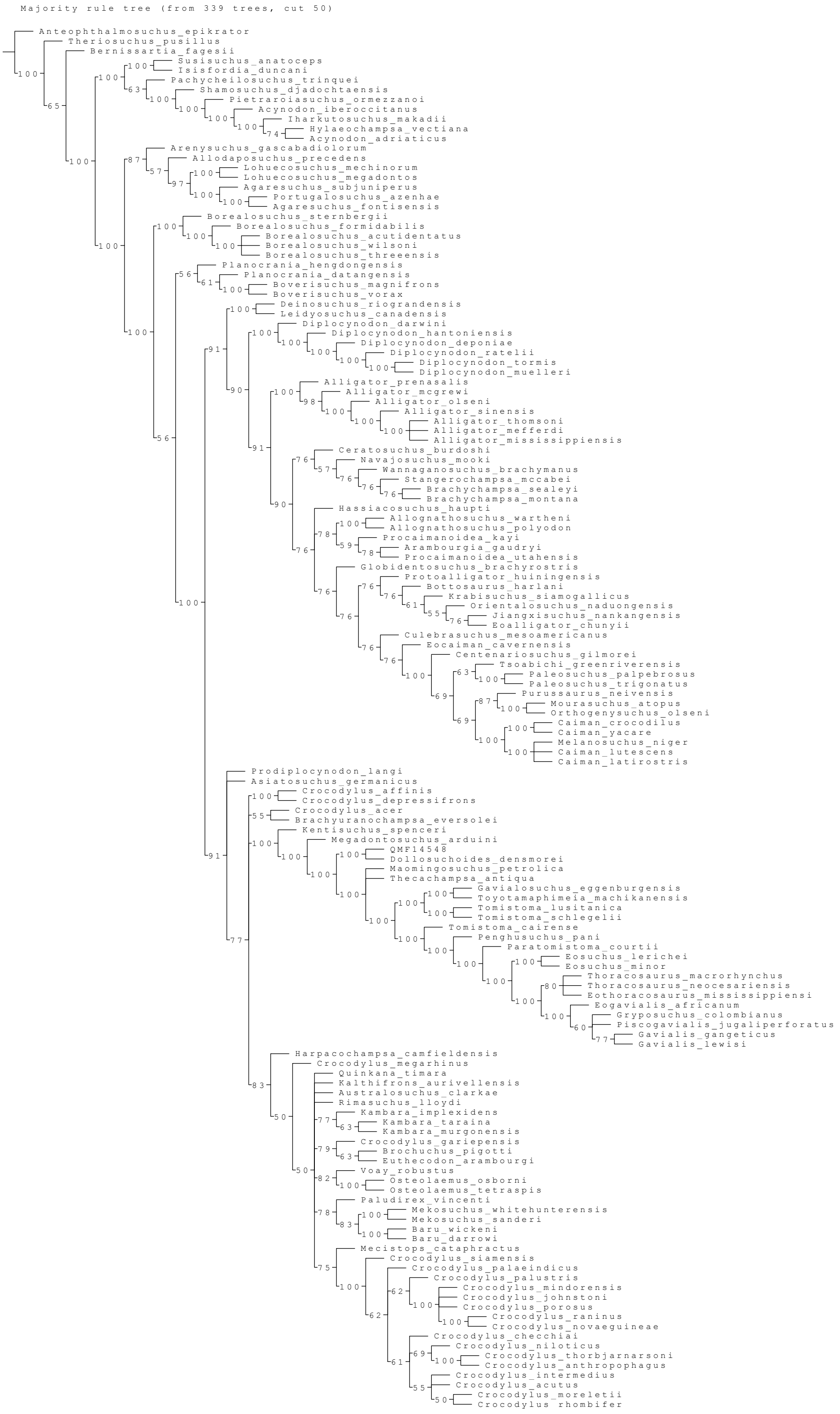

Figure S2.3 50% majority-rule consensus topology from the phylogenetic analysis performed under the EW method. In the cladogram, *Gunggamarandu maunala* gen. et sp. nov. is the OTU named QMF14548.

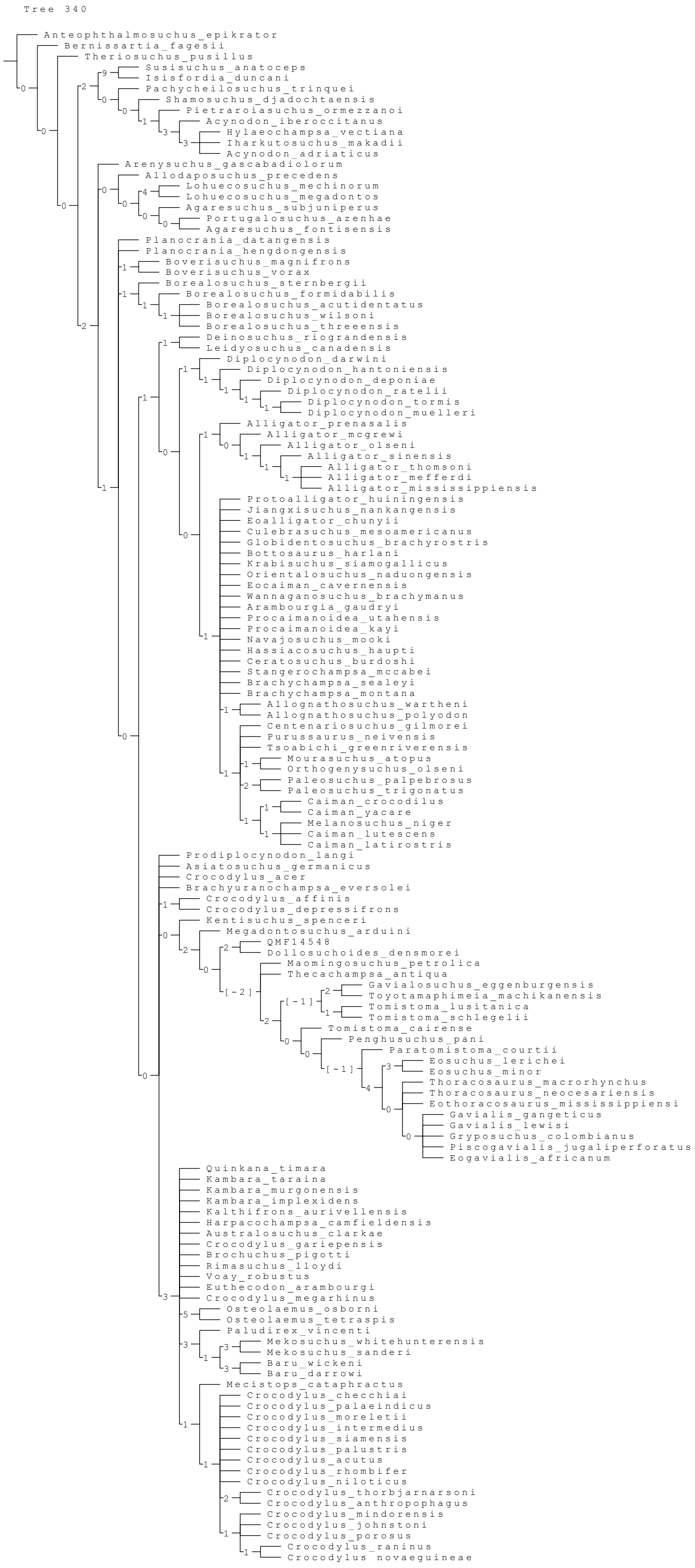

Figure S2.4 Bremer support results from the phylogenetic analysis performed under the EW method. In the cladogram, *Gunggamarandu maunala* gen. et sp. nov. is the OTU named QMF14548.

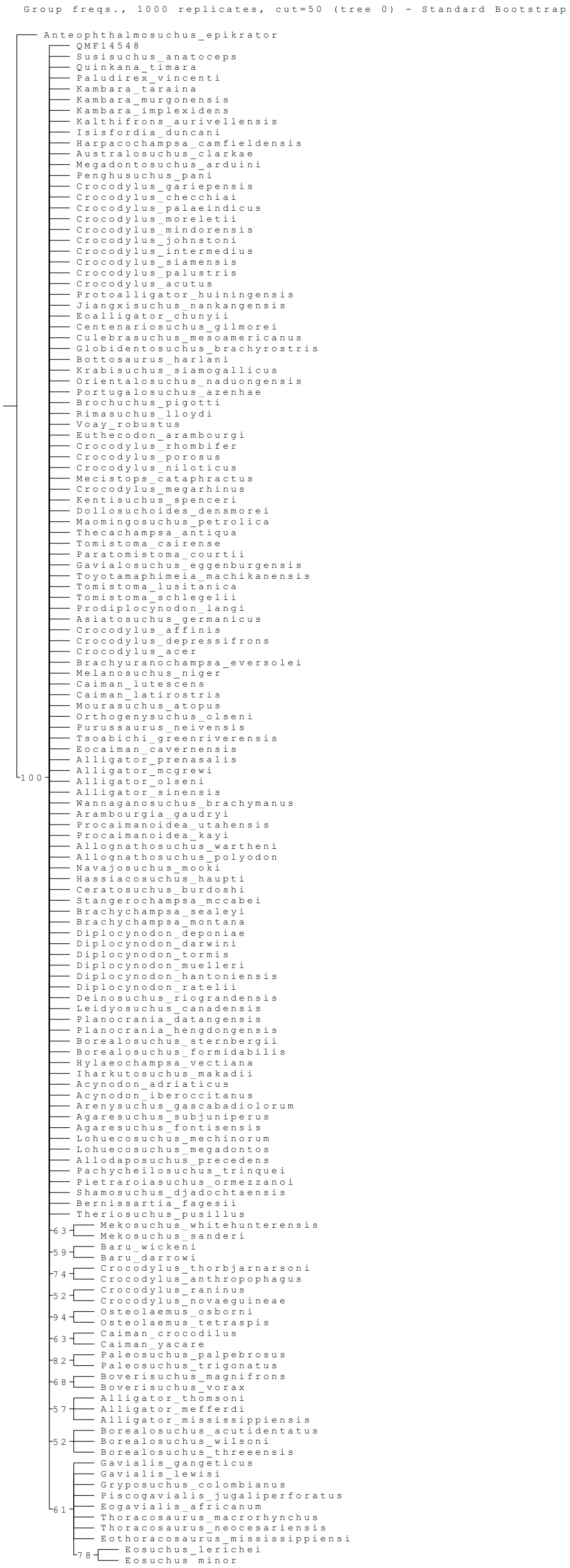

Figure S2.5 **Bootstrap support results from the phylogenetic analysis performed under the EW method.** In the cladogram, *Gunggamarandu maunala* gen. et sp. nov. is the OTU named QMF14548.

Implied Weighting analyses

The analysis that utilized the IW method with the concavity constant k = 3 (which is the default k value in TNT) recovered 9 MPCs with a length of 109.83161 (CI = 0.220; RI = 0.713). In general, the strict consensus topology is better resolved than that from the analysis that was run under the EW method (**Fig. S2.6**). Unlike the EW analysis, a monophyletic Gavialoidea that includes tomistomines was not recovered. Here, gavialids and 'thoracosaurs' are the basal-most clade within Crocodylia. Tomistominae is found within Crocodyloidea, and the latter, together with Alligatoroidea forms an even larger clade – Brevirostres. The nodal support from this analysis is quite poor (**Figs. S2.7** and **S2.8**). Tomistominae is monophyletic, except for *Kentisuchus spenceri* who is found as a sister taxon to *Mecistops cataphractus*. Tomistominae (with the exception of *Ke. spenceri*) is weakly supported (Bremer = 0; bootstrap = <50%), and characterized by five synapomorphies: dentary symphysis extends behind eighth alveolus (character 49, state 2); palatine process in the form of a thin wedge (character 116, state 1); massive postorbital bar (character 133, state 0); dorsal edges of orbits upturned (character 137, state 1); and, dorsal and ventral rims of squamosal groove for external ear valve musculature flare anteriorly (character 147, state 1). *Gunggamarandu maunala* is again found to be in a basal position within Tomistominae and the sister taxon to *D. densmorei*. The *Gunggamarandu* + *Dollosuchoides* clade is the best supported clade within Tomistominae (Bremer = 3; bootstrap = <50%), and characterized by one synapomorphy: a slender postfenestral bar, with a minimum thickness that is less than 8% the width of the cranial table (character 209, state 2). No autapomorphies were detected for *G. maunala* in this analysis.

The analysis that utilized the IW method with the concavity constant, k = 12 recovered 20 MPCs with a length of 54.59498 (CI = 0.226; RI = 0.722). This time, the strict consensus topology is mostly resolved (**Fig. S2.9**). In this analysis, gavialids and 'thoracosaurs' are found in a basal position within Crocodylia, having *Portugalosuchus azenhae* as their sister taxon. In addition, the group formed by the species of *Borealosuchus* Brochu, 1997a is the sister clade to *Portugalosuchus*

azenhae + Gavialoidea (Gavialidae + 'thoracosaurs'). Tomistominae is here recovered as a monophyletic clade within Crocodyloidea, with *Ke. spenceri* as its basal-most taxon. Nodal support is generally quite weak (**Figs. S2.10** and **S2.11**). Tomistominae is poorly supported (Bremer = 0; bootstrap = <50%), and characterized by four synapomorphies: palatine process in the form of a thin wedge (character 116, state 1); pterygoid ramus of ectopterygoid straight, with the posterolateral margin of the suborbital fenestra linear (character 119, state 0); maxilla with posterior process within lacrimal (character 128, state 1); and, the minimum width between the supratemporal fenestrae is 10% or less the total width of the cranial table (character 208, state 0). *Gunggamarandu maunala* is again found in a basal position within Tomistominae and the sister taxon to *D. densmorei*, although the support for this clade is poor (Bremer = 0; bootstrap = <50%). As in the EW analysis, the *Gunggamarandu* + *Dollosuchoides* clade is characterized by the same two synapomorphies, while no autapomorphies are indicated for *G. maunala*.

The analysis that utilized the IW method with the concavity constant, k = 25 recovered 7 MPCs with a length of 32.56997 steps (CI = 0.227; RI = 0.724). The strict consensus topology (**Fig. S2.12**) is mostly resolved and similar to the one from the previous analysis (IW k = 12), as well as the one yielded by the IW analysis of Ristevski *et al.* (2020a, b). Again, gavialids and 'thoracosaurs' are found in a basal position within Crocodylia, having *Portugalosuchus azenhae* as their sister taxon. Also, the group formed by the species of *Borealosuchus* is found as the sister clade to *Portugalosuchus azenhae* + Gavialoidea (Gavialidae + 'thoracosaurs'). Tomistominae is monophyletic and once again recovered within Crocodyloidea, who together with Alligatoroidea form Brevirostres. Nodal support is still quite weak (**Figs. S2.13** and **S2.14**). In the IW k = 25 analysis, Tomistominae is poorly supported (Bremer = 0; bootstrap = <50%), and characterized by the same four synapomorphies as in the previous (IW k = 12) analysis. *Gunggamarandu maunala* is once again found to be in a basal position within Tomistominae and the sister taxon to *D. densmorei*. The *Gunggamarandu* + *Dollosuchoides* clade is one of the best supported clades within Tomistominae (Bremer = 2; bootstrap = <50%). As in the EW and the IW k = 12 analyses, the

Gunggamarandu + *Dollosuchoides* clade is characterized by the same two synapomorphies, while no autapomorphies are indicated for *G. maunala*.

In both the IW *k* = 12 and IW *k* = 25 analysis, Mekosuchinae is found to be monophyletic, with *Kambara* spp. occupying the basal position in the group. In the IW *k* = 25 analysis, the nodal support for Mekosuchinae is not particularly strong (Bremer = 1; bootstrap = <50%). The results from the IW *k* = 25 analysis indicate four synapomorphies for Mekosuchinae: incisive foramen completely situated far from premaxillary tooth row, at the level of the second or third alveolus, or posterior (character 89, state 0); pterygoid ramus of ectopterygoid straight, with the posterolateral margin of the suborbital fenestra linear (character 119, state 0); quadrate with a small medial hemicondyle and a dorsal notch for foramen aëreum (character 181, state 1); and, anteriorly directed ridges on the pterygoid plates that extend from the lateral margins of the secondary choana (character 217, state 1).

The only Cenozoic Australian taxon that does not cluster with the other Australian OTUs in any of the performed analyses is *Harpacochampsa camfieldensis*. The exception was the IW *k* = 3 analysis who found *H. camfieldensis* forming a clade with *Paludirex vincenti* in a basal position in Crocodyloidea. In the IW *k* = 3 analysis, the *H. camfieldensis* + *P. vincenti* clade has extremely weak nodal support (Bremer = -2; bootstrap = <50%) and is united by only one synapomorphy: postorbital neither contacts quadrate nor quadratojugal medially (character 143, state 0). Similar to the result from the IW analysis of Ristevski *et al.* (2020a, b), the other IW analyses again found *H. camfieldensis* to be within Crocodyloidea and not clustering with tomistomines like *Gunggamarandu* nor with mekosuchines. Instead, in the IW *k* = 25 analysis (as well as the IW *k* = 12 analysis), *H. camfieldensis* is located at the base of Crocodyloidea, with *Prodiplocynodon langi* being the only crocodyloid in a more basal position. In the IW *k* = 12 and *k* = 25 analyses, *H. camfieldensis* is characterized by 11 autapomorphies: anterior dentary teeth strongly procumbent (character 48, state 0); maxilla broadly separates the ectopterygoid from the maxillary tooth row (character 104, state 1); massive postorbital bar (character 133, state 0); ventral margin of

postorbital bar flush with the lateral jugal surface (character 135, state 0); postorbital neither contacts quadrate nor quadratojugal medially (character 143, state 0); quadratojugal bears modest process, or none at all, along lower temporal bar (character 144, state 1); quadratojugal does not extend to superior angle of infratemporal fenestra (character 145, state 1); caudal margin of meatal chamber smooth and continuous with the paroccipital process (character 149, state 0); squamosal extends ventrolaterally to lateral extent of the paraoccipital process (character 159, state 1); anterior maxillary teeth with ridges on their labial surface (character 197, state 1); and, the alveolar process of the maxilla along the first six maxillary alveoli is inconspicuous or low, with the medial lamina comprising no more than 10% of the total rostral height (character 202, state 0).

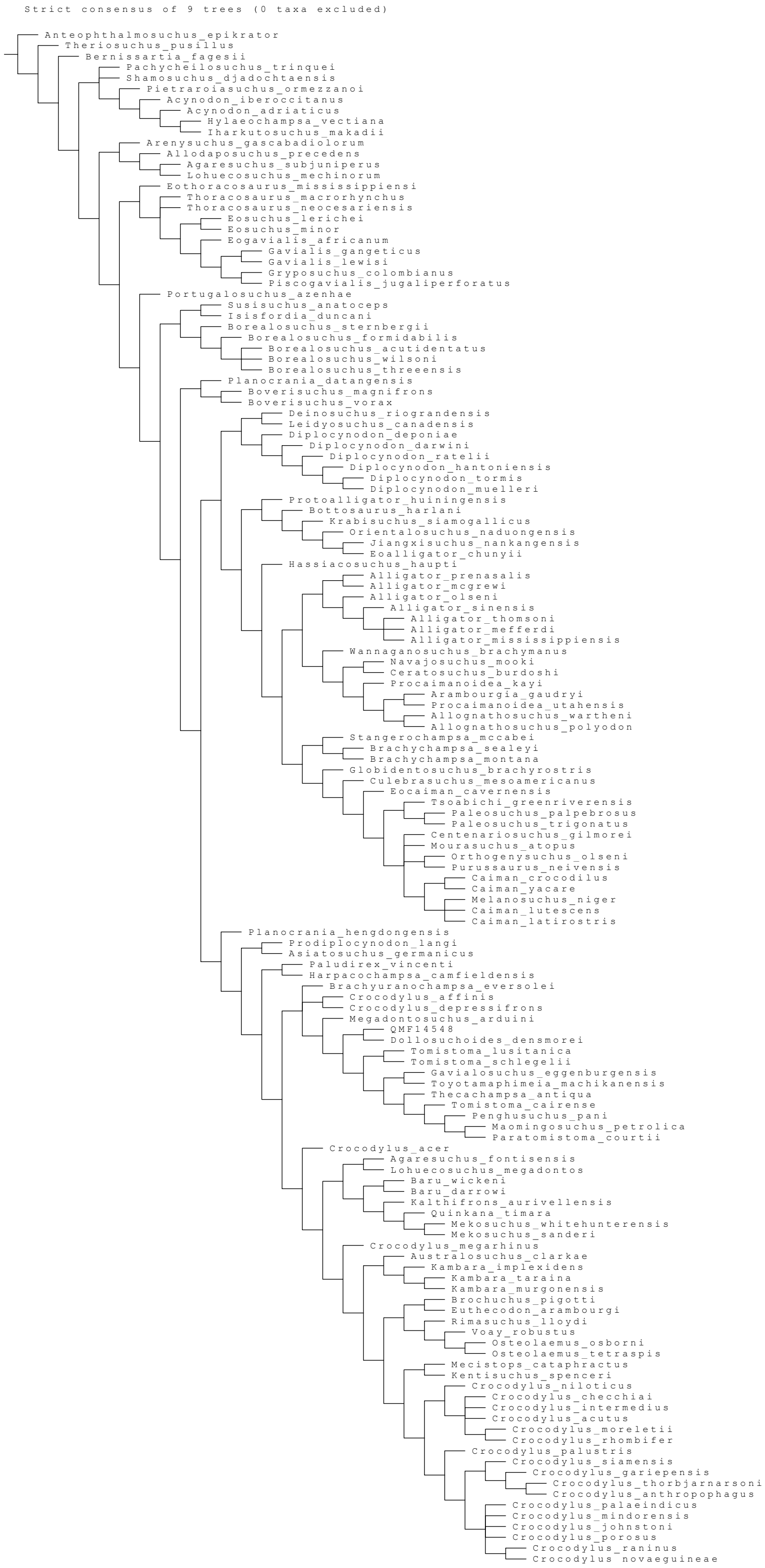

Figure S2.6 Strict consensus topology from the phylogenetic analysis performed under the IW (k = 3) method. In the cladogram, *Gunggamarandu maunala* gen. et sp. nov. is the OTU named QMF14548.

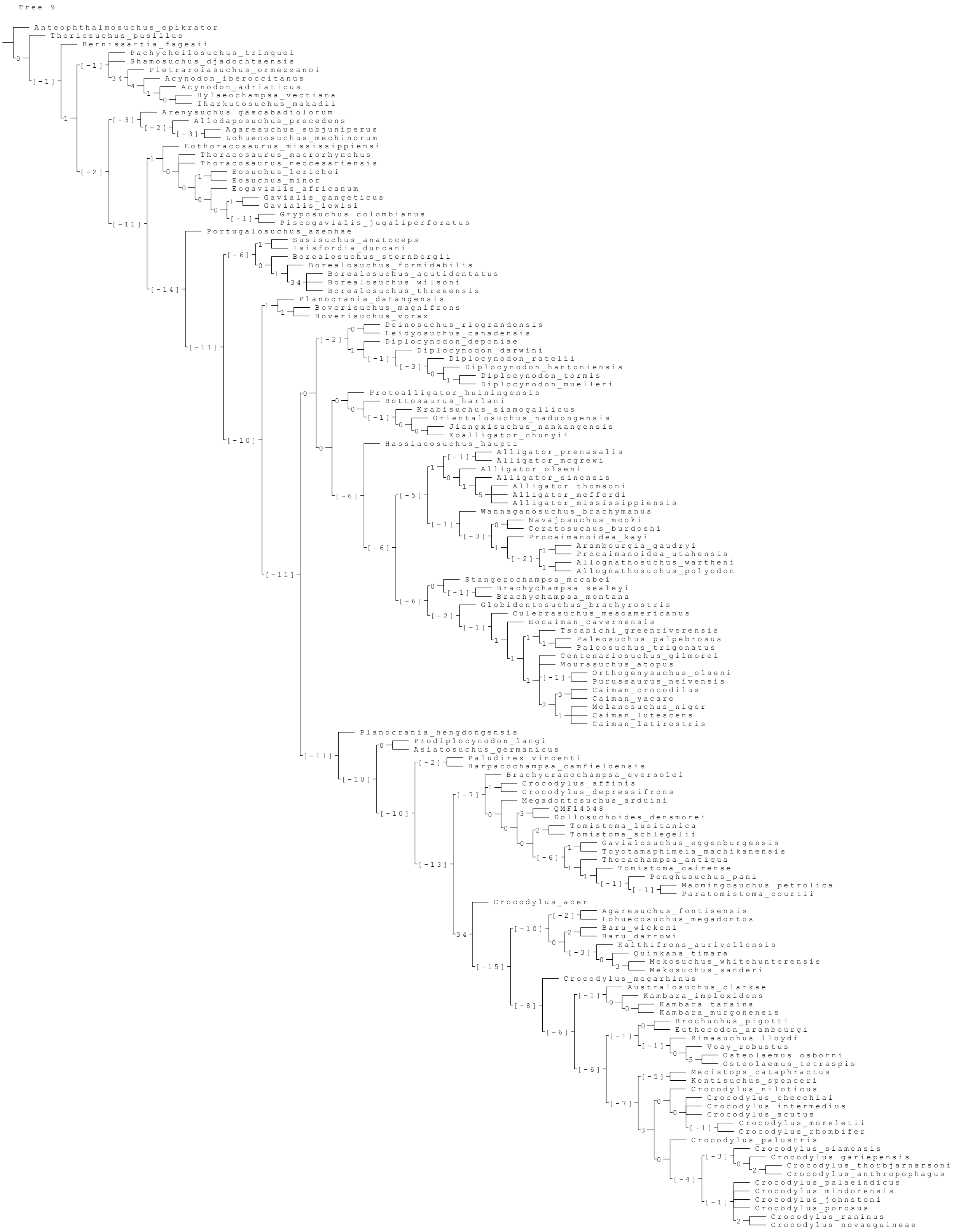

Figure S2.7 Bremer support results from the phylogenetic analysis performed under the IW (k = 3) method.

In the cladogram, *Gunggamarandu maunala* gen. et sp. nov. is the OTU named QMF14548.

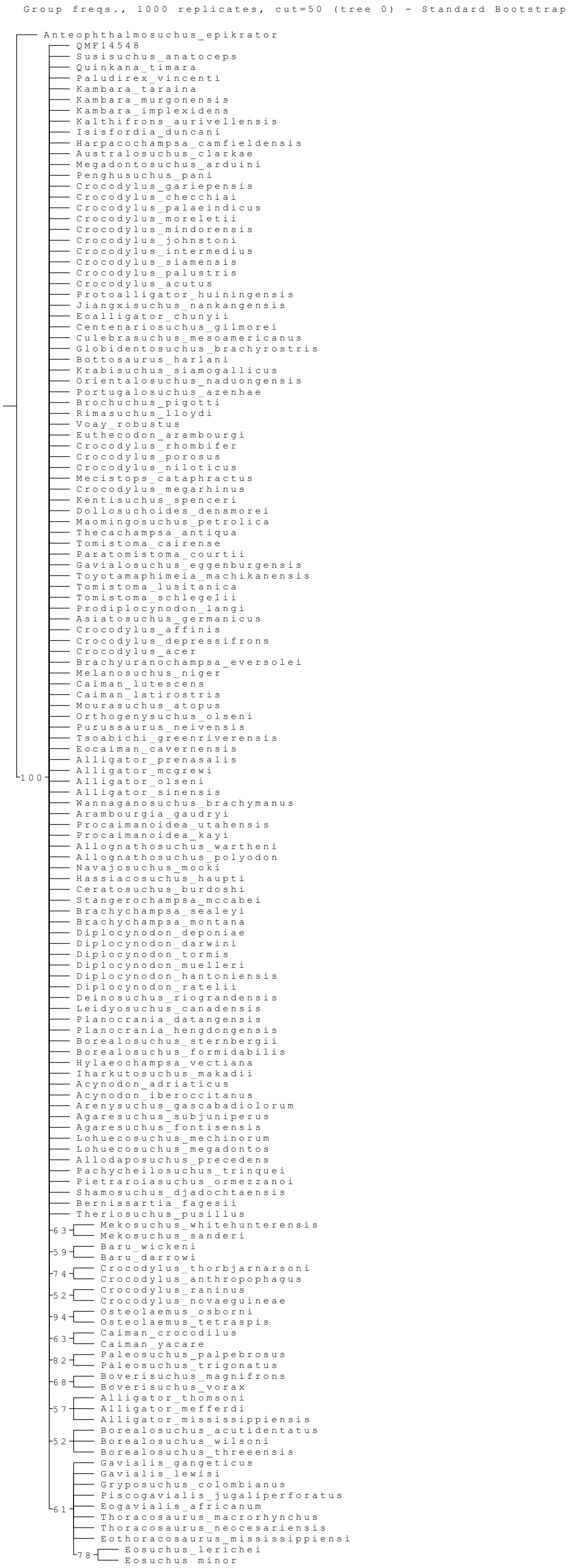

Figure S2.8 Bootstrap support results from the phylogenetic analysis performed under the IW (k = 3) method. In the cladogram, *Gunggamarandu maunala* gen. et sp. nov. is the OTU named QMF14548.

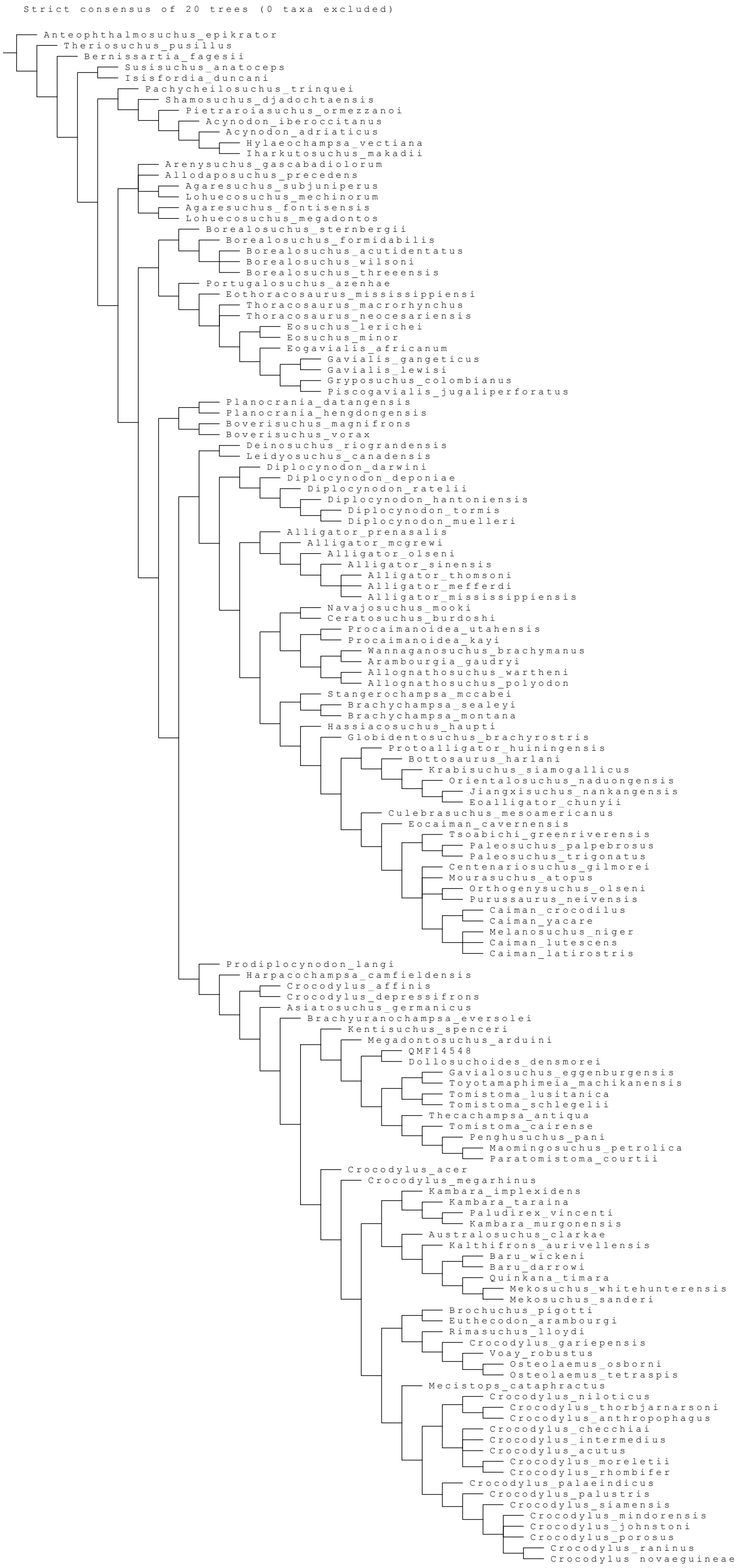

Figure S2.9 Strict consensus topology from the phylogenetic analysis performed under the IW (k = 12) method. In the cladogram, *Gunggamarandu maunala* gen. et sp. nov. is the OTU named QMF14548.

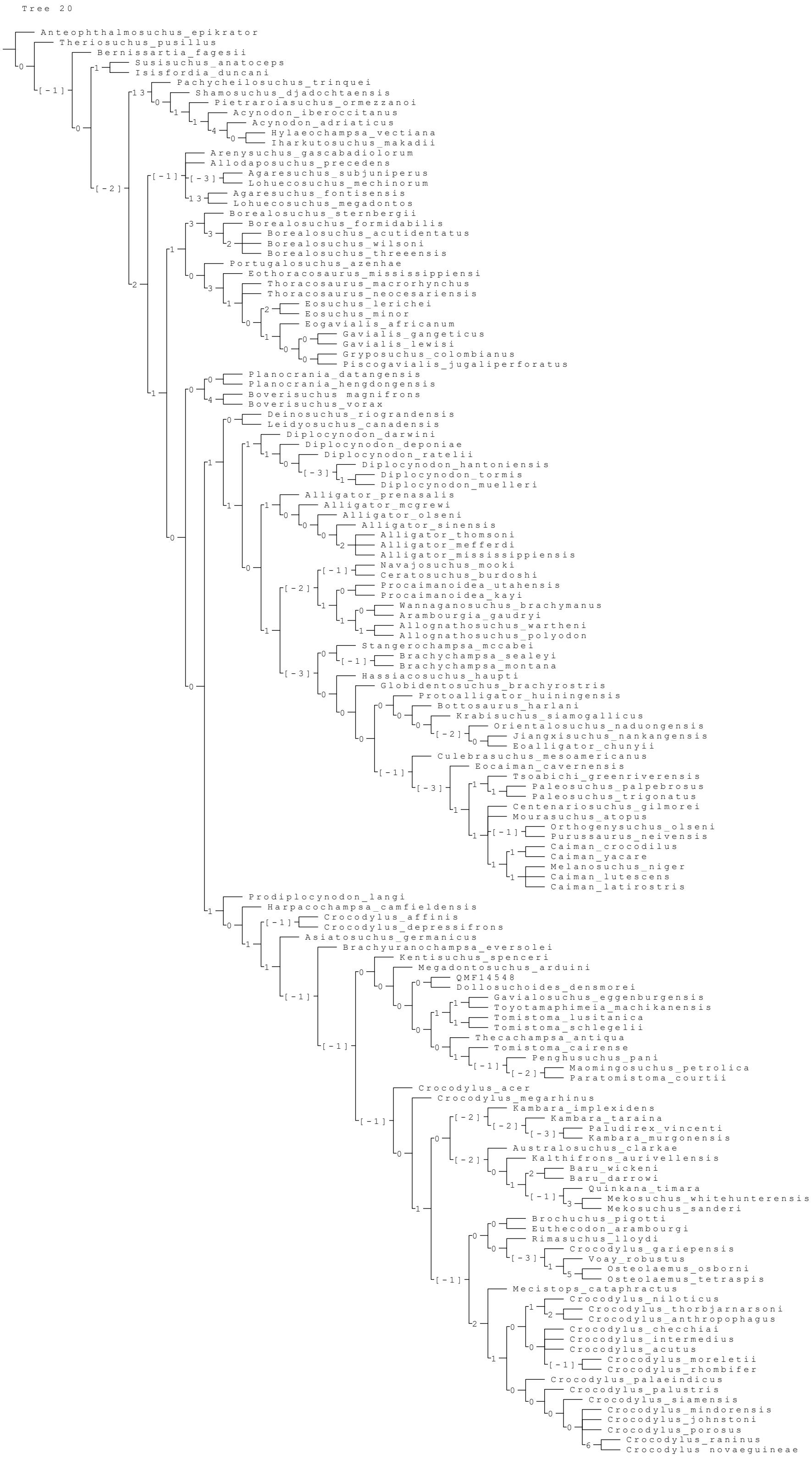

Figure S2.10 Bremer support results from the phylogenetic analysis performed under the IW (k = 12) method. In the cladogram, *Gunggamarandu maunala* gen. et sp. nov. is the OTU named QMF14548.

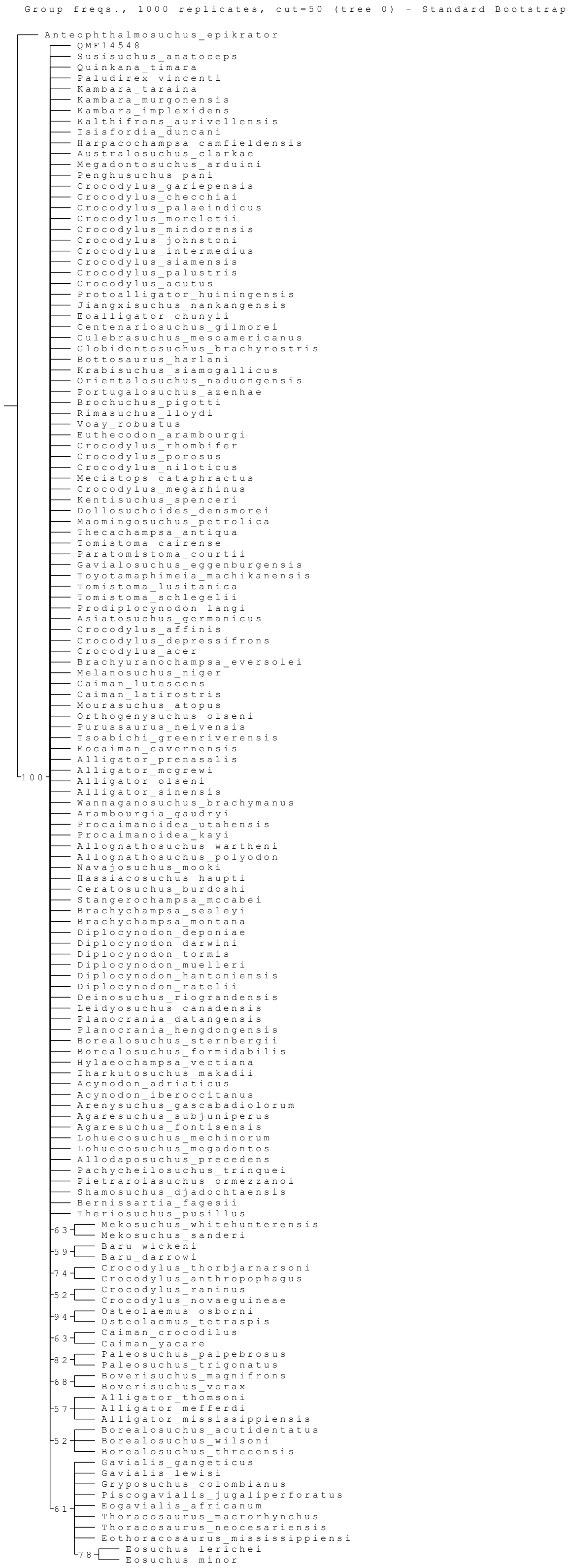

Figure S2.11 Bootstrap support results from the phylogenetic analysis performed under the IW (k = 12) method. In the cladogram, *Gunggamarandu maunala* gen. et sp. nov. is the OTU named QMF14548.

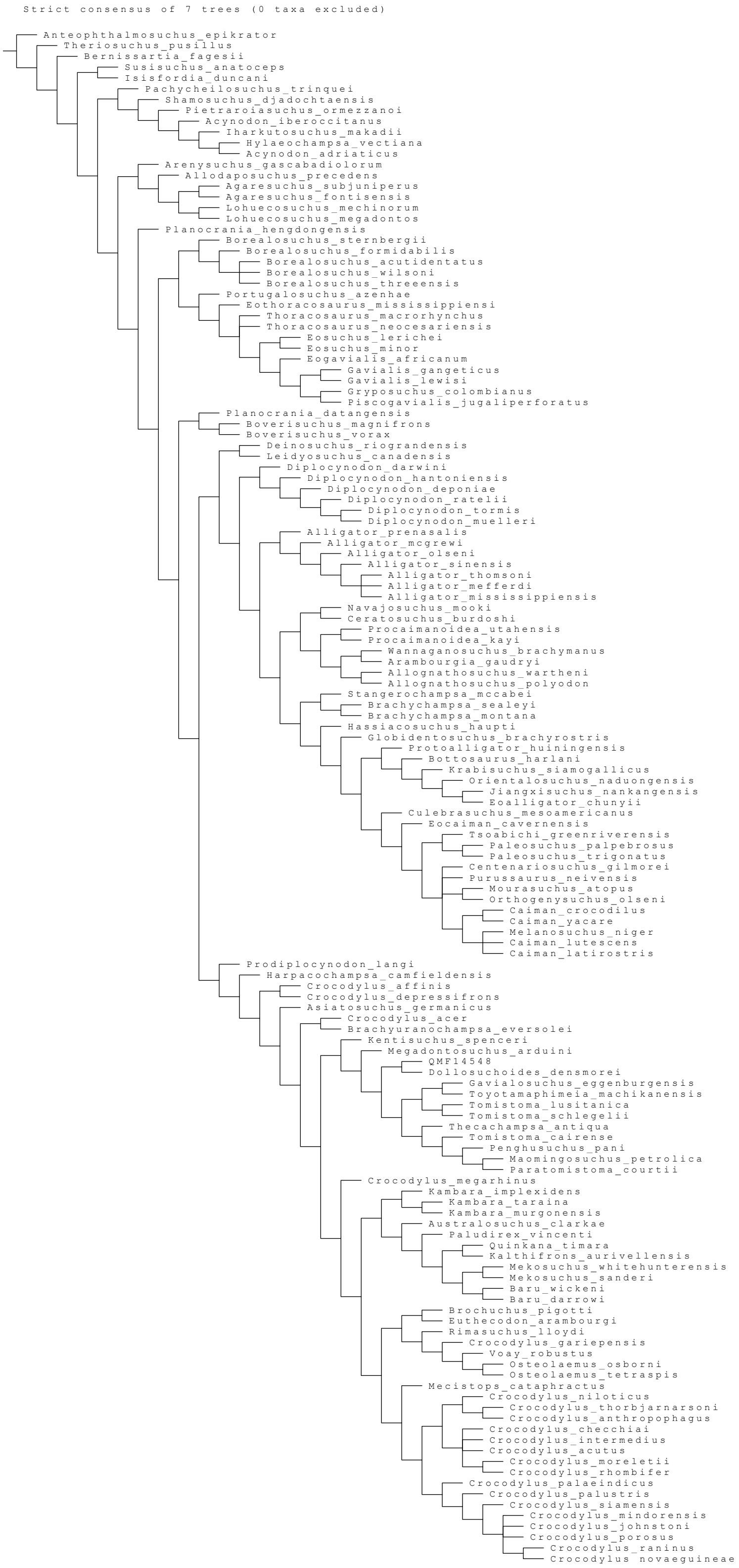

Figure S2.12 Strict consensus topology from the phylogenetic analysis performed under the IW (k = 25) method. In the cladogram, *Gunggamarandu maunala* gen. et sp. nov. is the OTU named QMF14548.

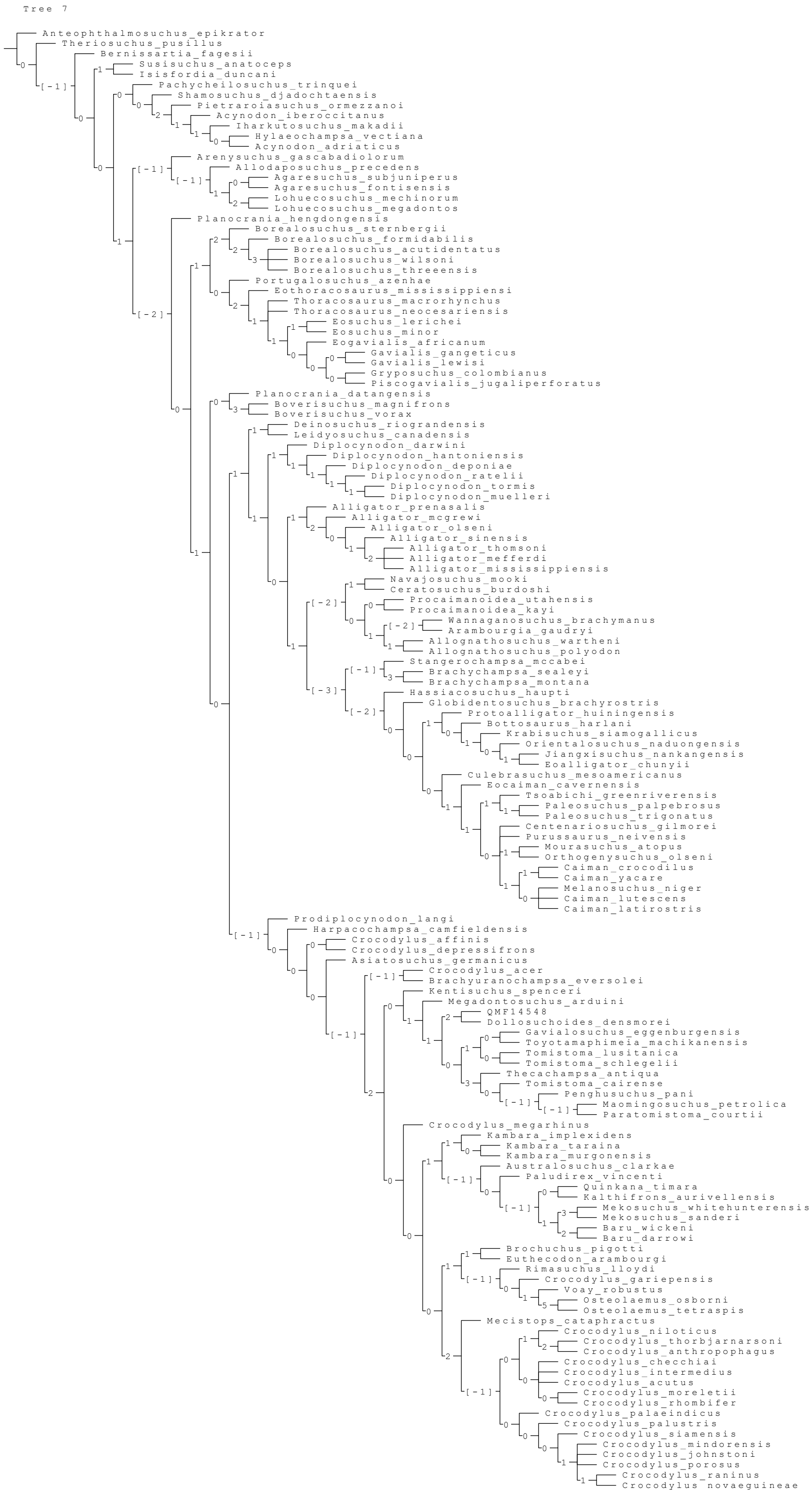

Figure S2.13 Bremer support results from the phylogenetic analysis performed under the IW (k = 25) method. In the cladogram, *Gunggamarandu maunala* gen. et sp. nov. is the OTU named QMF14548.

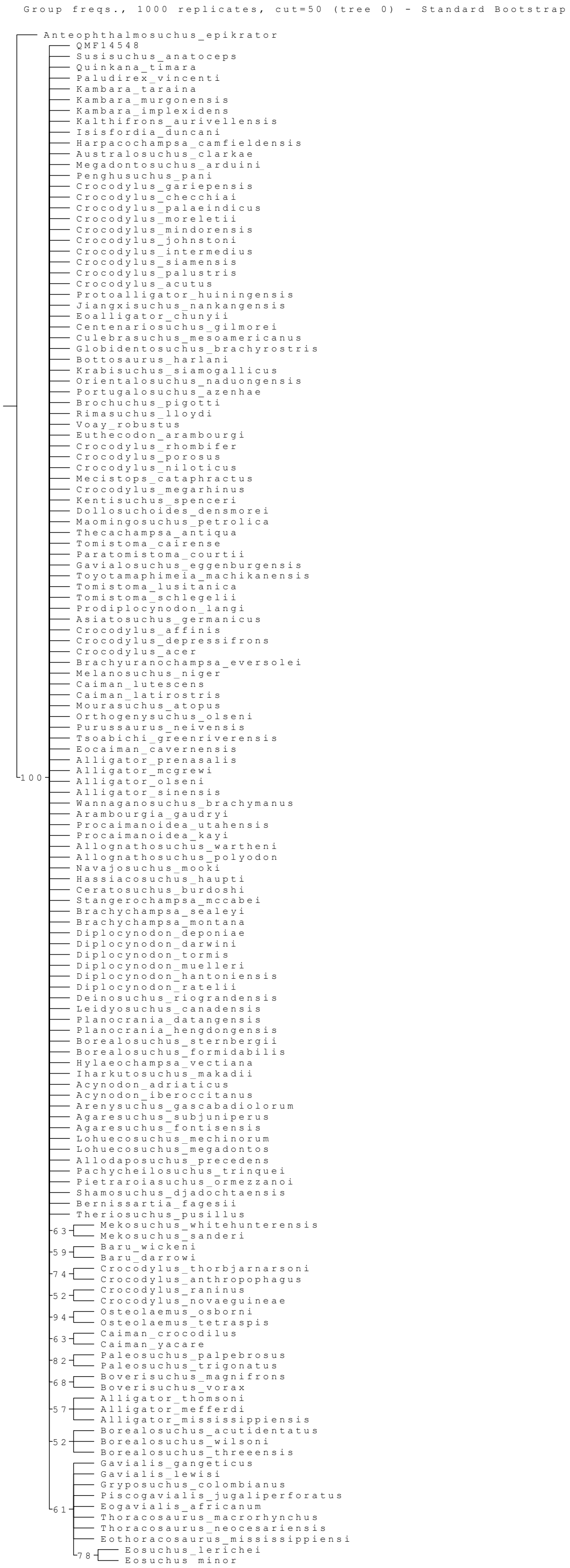

Figure S2.14 Bootstrap support results from the phylogenetic analysis performed under the IW (k = 25) method. In the cladogram, *Gunggamarandu maunala* gen. et sp. nov. is the OTU named QMF14548.

Scored characters for the Australian crocodyliform operational taxonomic units

Below is a table showing the number and percentage of scored characters for the Australian crocodyliform OTUs in the matrix. The taxa are listed in alphabetical order.

Taxon	Characters scored (out of 229)	Characters scored (in %)
Australosuchus clarkae	151	65.94%
Baru darrowi	93	40.61%
Baru wickeni	131	57.2%
Crocodylus johnstoni	228	99.56%
Crocodylus porosus	228	99.56%
Gunggamarandu maunala	25	10.92%
Harpacochampsa camfieldensis	80	34.93%
Isisfordia duncani	143	62.45%
Kalthifrons aurivellensis	62	27.07%
Kambara implexidens	160	69.97%
Kambara murgonensis	100	43.67%
Kambara taraina	118	51.53%
Mekosuchus sanderi	71	31%
Mekosuchus whitehunterensis	46	20.09%
Paludirex vincenti	78	34.06%
Quinkana timara	88	38.43%

INSTITUTIONAL ABBREVIATIONS

CMC, Chinchilla Museum Collection, Chinchilla, Queensland, Australia

FMNH, Field Museum of Natural History, Chicago, Illinois, U. S. A.

IRSNB, Institut Royal des Sciences Naturelles de Belgique, Brussels, Belgium

IWCMS, Isle of Wight County Museums Services (Dinosaur Isle Museum and visitor attraction) Sandown, England, United Kingdom

KNM, National Museums of Kenya, Nairobi, Kenya

MGPD, Museo di Geologia e Paleontologia, Università degli Studi di Padova, Padua, Italy

NHMUK, Natural History Museum, London, England, United Kingdom (OR, old register; R, reptiles)

NMB, National Museum of the Bahamas, Nassau, Commonwealth of the Bahamas

NMV, Museum Victoria, Melbourne, Victoria, Australia

NTM, Museum and Art Gallery of the Northern Territory, Darwin and Alice Springs, Northern Territory, Australia (P, palaeontology; R, reptile collection)

QM, Queensland Museum, Brisbane, Queensland, Australia (F, fossil)

SAM, South Australian Museum, Adelaide, South Australia, Australia (P, palaeontology)

TMM, Texas Memorial Museum, Austin, Texas, U. S. A.

UF, University of Florida Museum of Natural History, Gainesville, Florida, U. S. A. (H, herpetology division)

UQ, University of Queensland, Brisbane, Queensland, Australia

UQSSAL, University of Queensland Steven Salisbury collection, Brisbane, Queensland, Australia

REFERENCES

Aguilera, O. A., Riff, D., & Bocquentin-Villanueva, J. (2006). A new giant *Purussaurus* (Crocodyliformes, Alligatoridae) from the Upper Miocene Urumaco Formation, Venezuela. *Journal of Systematic Palaeontology*, *4*(3), 221–232.

de Andrade, M. B., Edmonds, R., Benton, M. J., & Schouten, R. (2011). A new Berriasian species of *Goniopholis* (Mesoeucrocodylia, Neosuchia) from England, and a review of the genus. *Zoological Journal of the Linnean Society*, *163*, S66–S108.

Andrews, C. W. (1901). II.—Preliminary note on some recently discovered extinct vertebrates from Egypt. (Part II.). *Geological Magazine*, *8*(10), 436–444.

Andrews, C. W. (1905). I.—Notes on some new Crocodilia from the Eocene of Egypt. *Geological Magazine*, *2*(11), 481–484.

Antunes, M. T. (1961). *Tomistoma lusitanica*, crocodilian du Miocène du Portugal. *Revista de Faculdade de Ciencias, Universidade de Lisboa*, *9*(*ser. 2*), 5–88.

Bakker, R. T., Williams, M., & Currie, P. J. (1988). *Nanotyrannus*, a new genus of pygmy tyrannosaur, from the latest Cretaceous of Montana. *Hunteria*, *1*(5), 1–30.

Balouet, J.-C., & Buffetaut, E. (1987). *Mekosuchus inexpectatus*, n. g., n. sp., Crocodilien nouueau de I'Holocène de Nouvelle Calédonie. *Comptes rendus de l'Académie des sciences. Série 2, Mécanique, Physique, Chimie, Sciences de l'univers, Sciences de la Terre*, *304*(14), 853–856.

Bartels, W. S. (1984). Osteology and systematic affinities of the horned alligator *Ceratosuchus* (Reptilia, Crocodilia). *Journal of Paleontology*, *58*(6), 1347–1353.

Benton, M. J., & Clark, J. M. (1988). Archosaur phylogeny and the relationships of the Crocodylia. Pp. 295–338 in M. Benton (ed.) *The phylogeny and classification of the tetrapods*, *Volume 1*, Clarendon Press, Oxford, United Kingdom.

Berg, D. E. (1966). Die Krokodile, insbesondere *Asiatosuchus* und aff. *Sebecus*?, aus dem Eozän von Messel bei Darmstadt/Hessen. *Abhandlungen des Hessischen Landesamtes für Bodenforschung*, *52*, 1–105.

Bickelmann, C., & Klein, N. (2009). The late Pleistocene horned crocodile *Voay robustus* (Grandidier & Vaillant, 1872) from Madagascar in the Museum für Naturkunde Berlin. *Fossil Record*, *12*(1), 13–21.

de Blainville, H. D. (1835). Systeme d'Herpetologie. *Nouvelles Annalesdu Museum d'Histoire Naturelle*, *4*, 37–295.

de Blainville, H. D. (1855). *Ostéographie: atlas du genre* Crocodilus. *Explication des planches*. Paris.

Bona, P., & Desojo, J. B. (2011). Osteology and cranial musculature of *Caiman latirostris* (Crocodylia: Alligatoridae). *Journal of Morphology*, *272*(7), 780–795.

Bona, P., Fernandez Blanco, M. V., Scheyer, T. M., & Foth, C. (2017). Shedding light on the taxonomic diversity of the South American Miocene caimans: the status of *Melanosuchus fisheri* (Crocodylia, Alligatoroidea). *Ameghiniana*, *54*(6), 681–687.

Brochu, C. A. (1997a). A review of "*Leidyosuchus*" (Crocodyliformes, Eusuchia) from the Cretaceous through Eocene of North America. *Journal of Vertebrate Paleontology*, *17*(4), 679–697.

Brochu, C. A. (1997b). Morphology, fossils, divergence timing, and the phylogenetic relationships of *Gavialis*. *Systematic Biology*, *46*(3), 479–522.

Brochu, C. A. (1997c). *Phylogenetic systematics and taxonomy of Crocodylia*. Unpublished PhD thesis, The University of Texas at Austin, Austin, 467 pp.

Brochu, C. A. (1999). Phylogenetics, taxonomy, and historical biogeography of Alligatoroidea. *Journal of Vertebrate Paleontology*, *19*(S2), 9–100.

Brochu, C. A. (2004a). A new Late Cretaceous gavialoid crocodylian from eastern North America and the phylogenetic relationships of thoracosaurs. *Journal of Vertebrate Paleontology*, *24*(3), 610–633.

Brochu, C. A. (2004b). Alligatorine phylogeny and the status of *Allognathosuchus* Mook, 1921. *Journal of Vertebrate Paleontology*, *24*(4), 857–873.

Brochu, C. A. (2006a). A new miniature horned crocodile from the Quaternary of Aldabra Atoll, Western Indian Ocean. *Copeia*, *2006*(2), 149–158.

Brochu, C. A. (2006b). Osteology and phylogenetic significance of *Eosuchus minor* (Marsh, 1870) new combination, a longirostrine crocodylian from the late Paleocene of North America. *Journal of Paleontology*, *80*(1), 162–186.

Brochu, C. A. (2007a). Morphology, relationships, and biogeographical significance of an extinct horned crocodile (Crocodylia, Crocodylidae) from the Quaternary of Madagascar. *Zoological Journal of the Linnean Society*, *150*, 835–863.

Brochu, C. A. (2007b). Systematics and taxonomy of Eocene tomistomine crocodylians from Britain and northern Europe. *Palaeontology*, *50*(4), 917–928.

Brochu, C. A. (2010). New alligatorid from the lower Eocene Green River Formation of Wyoming and the origin of caimans. *Journal of Vertebrate Paleontology*, *30*(4), 1109–1126.

Brochu, C. A. (2011). Phylogenetic relationships of *Necrosuchus ionensis* Simpson, 1937 and the early history of caimanines. *Zoological Journal of the Linnean society*, *163*(suppl_1), S228–S256.

Brochu, C. A. (2013). Phylogenetic relationships of Palaeogene ziphodont eusuchians and the status of *Pristichampsus* Gervais, 1853. *Earth and Environmental Science Transactions of the Royal Society of Edinburgh*, *103*(3–4), 521–550.

Brochu, C. A. (2020). Pliocene crocodiles from Kanapoi, Turkana Basin, Kenya. *Journal of Human Evolution*, *140*, 102410.

Brochu, C. A., & Gingerich, P. D. (2000). New tomistomine crocodylian from the middle Eocene (Bartonian) of Wadi Hitan, Fayum Province, Egypt. *Contributions from the Museum of Paleontology, the University of Michigan*, *30*(10), 251–268.

Brochu, C. A., & Storrs, G. W. (2012). A giant crocodile from the Plio-Pleistocene of Kenya, the phylogenetic relationships of Neogene African crocodylines, and the antiquity of *Crocodylus* in Africa. *Journal of Vertebrate Paleontology*, *32*(3), 587–602.

Brochu, C. A., Njau, J., Blumenschine, R. J., & Densmore, L. D. (2010). A new horned crocodile from the Plio-Pleistocene hominid sites at Olduvai Gorge, Tanzania. *PLoS ONE*, *5*(2), e9333.

Brochu, C. A., Parris, D. C., Grandstaff, B. S., Denton Jr, R. K., & Gallagher, W. B. (2012). A new species of *Borealosuchus* (Crocodyliformes, Eusuchia) from the Late Cretaceous–Early Paleogene of New Jersey. *Journal of Vertebrate Paleontology*, *32*(1), 105–116.

Buchanan, L. A. (2009). *Kambara taraina* sp. nov. (Crocodylia, Crocodyloidea), a new Eocene mekosuchine from Queensland, Australia, and a revision of the genus. *Journal of Vertebrate Paleontology*, *29*(2), 473–486.

Buckland, W. (1836). *Geology and Mineralogy Considered with Reference to Natural Theology*. Pickering, London, 618 pp.

Buffetaut, E. (1975). Sur l'anatomie et la position systématique de *Bernissartia fagesii* Dollo, L., 1883, crocodilien du Wealdien de Bernissart, Belgique. *Bulletin de l'Institut Royal des Sciences Naturelles de Belgique*, *51*(2), 1–20.

Busbey, A. B. III. (1977). *Functional morphology of the head of* Pristichampsus vorax *(Crocodilia, Eusuchia) from the Eocene of North America.* Unpublished Master of Arts thesis, The University of Texas at Austin, Austin, 102 pp.

Buscalioni, A. D., Sanz, J. L., & Casanovas, M. L. (1992). A new species of the eusuchian crocodile *Diplocynodon* from the Eocene of Spain. *Neues Jahrbuch für Geologie und Paläontologie, Abhandlungen*, *187*(1), 1–29.

Buscalioni, A. D., Ortega, F., & Vasse, D. (1997). New crocodiles (Eusuchia: Alligatoroidea) from the Upper Cretaceous of southern Europe. *Comptes Rendus de l'Académie des Sciences, Série IIA - Earth & Planetary Sciences, 325*(7), 525–530.

Buscalioni A. D., Piras, P., Vullo, R., Signore, M., & Barbera, C. (2011). Early eusuchia crocodylomorpha from the vertebrate-rich Plattenkalk of Pietraroia (Lower Albian, southern Apennines, Italy). *Zoological Journal of the Linnean Society*, *163*, S199–S227.

Buttmann, H. (1826). *De musculis crcodili*. Inaugral-Dissertation, Universität Halle, 32 pp.

Carpenter, K. (1983). *Thoracosaurus neocesariensis* (De Kay, 1842) (Crocodylia: Crocodylidae) from the Late Cretaceous Ripley Formation of Mississippi. *Mississippi Geology*, *4*(1), 1–10.

Case, E. C. (1925). Note on a new species of the Eocene crocodilian *Allognathosuchus*, *A. wartheni*. *Contributions from the Museum of Geology, University of Michigan*, *2*(5), 93–97.

Cidade, G. M., Solórzano, A., Rincón, A. D., Riff, D., & Hsiou, A. S. (2017). A new *Mourasuchus* (Alligatoroidea, Caimaninae) from the late Miocene of Venezuela, the phylogeny of Caimaninae and considerations on the feeding habits of *Mourasuchus*. *PeerJ*, *5*, e3056.

Clark, J. M. (1986). *Phylogenetic relationships of the crocodylomorph archosaurs*. Unpublished PhD thesis, The University of Chicago, Chicago, 556 pp.

Clark, J. M. (1994). Patterns of evolution in Mesozoic Crocodyliformes. Pp. 84–97 in N. C. Fraser & H.-D. Sues (eds.) *In the Shadow of the Dinosaurs: Early Mesozoic Tetrapods*, Cambridge, New York, U. S. A.

Clark, J. M., & Norell, M. A. (1992). The Early Cretaceous crocodylomorph *Hylaeochampsa vectiana* from the Wealden of the Isle of Wight. *American Museum Novitates*, *3032*, 1–19.

Colbert, E. H., & Bird, R. T. (1954). A gigantic crocodile from the Upper Cretaceous beds of Texas. *American Museum Novitates*, *1688*, 1–22.

Conrad, J. L., Jenkins, K., Lehmann, T., Manthi, F. K., Peppe, D. J., Nightingale, S., Cossette, A., Dunsworth, H. M., Harcourt-Smith, W. E. H., & McNulty, K. P. (2013). New specimens of '*Crocodylus*' *pigotti* (Crocodylidae) from Rusinga Island, Kenya, and generic reallocation of the species. *Journal of Vertebrate Paleontology*, *33*(3), 629–646.

Cope, E. D. (1861). Recent species of Emydosaurian reptiles represented in the Museum of the Academy. *Proceedings of the Academy of Natural Sciences of Philadelphia*, *1860*, 549–551.

Cope, E. D. (1882). The reptiles of the American Eocene. *The American Naturalist*, *16*(12), 979–993.

Cope, E. D. (1873). On the extinct Vertebrata of the Eocene of Wyoming, observed by the expedition of 1872, with notes on the geology. *Annual Report of the U. S. Geological and Geographic Survey of the Territories*, *6*, 545–649.

Cossette, A. P., & Brochu, C. A. (2018). A new specimen of the alligatoroid *Bottosaurus harlani* and the early history of character evolution in alligatorids. *Journal of Vertebrate Paleontology*, *38*(4), 1–22.

Cossette, A. P., & Brochu, C. A. (2020). A systematic review of the giant alligatoroid *Deinosuchus* from the Campanian of North America and its implications for the relationships at the root of Crocodylia. *Journal of Vertebrate Paleontology*, e1767638.

Cuvier, G. L. (1807). Sur les différentes espèces de crocodiles vivans et sur leurs caractères distinctifs. *Annales du Muséum National d'Histoire Naturelle, 10*, 8–86.

Cuvier, G. L. (1824). *Recherches Sur Les Ossemens Fossiles. Vol. 5. 2eme*. G. Dufour & E. d'Ocagne Libraries, Paris, 185 pp.

Das, I., & Charles, J. 2002. New record of a freshwater crocodile from Brunei. *IUCN/SSC Crocodile Specialist Group Newsletter*, *21*, 10–11.

Daudin, F. M. (1802). *Histoire Naturelle, Générale et Particulière des Reptiles; ouvrage faisant suit à l'Histoire naturell générale et particulière, composée par Leclerc de Buffon; et rédigee par C. S. Sonnini, membre de plusieurs sociétés savantes*. Vol. 2. F. Dufart, Paris [1802], 432 pp.

Delfino, M., & De Vos, J. (2010). A revision of the Dubois crocodylians, *Gavialis bengawanicus* and *Crocodylus ossifragus*, from the Pleistocene *Homo erectus* beds of Java. *Journal of Vertebrate Paleontology*, *30*(2), 427–441.

Delfino, M., Piras, P., & Smith, T. (2005). Anatomy and phylogeny of the gavialoid crocodylian *Eosuchus lerichei* from the Paleocene of Europe. *Acta Palaeontologica Polonica*, *50*(3), 565–580.

Delfino, M., Codrea, V., Folie, A., Dica, P., Godefroit, P., & Smith, T. (2008a). A complete skull of *Allodaposuchus precedens* Nopcsa, 1928 (Eusuchia) and a reassessment of the morphology

of the taxon based on the Romanian remains. *Journal of Vertebrate Paleontology*, *28*(1), 111–122.

Delfino, M., Martin, J. E., & Buffetaut, E. (2008b). A new species of *Acynodon* (Crocodylia) from the Upper Cretaceous (Santonian–Campanian) of Villaggio del Pescatore, Italy. *Palaeontology*, *51*(5), 1091–1106.

Delfino, M., & Smith, T. (2009). A reassessment of the morphology and taxonomic status of '*Crocodylus*' *depressifrons* Blainville, 1855 (Crocodylia, Crocodyloidea) based on the Early Eocene remains from Belgium. *Zoological Journal of the Linnean Society*, *156*(1), 140–167.

Delfino, M., & Smith, T. (2012). Reappraisal of the morphology and phylogenetic relationships of the middle Eocene alligatoroid *Diplocynodon deponiae* (Frey, Laemmert, and Riess, 1987) based on a three-dimensional specimen. *Journal of Vertebrate Paleontology*, *32*(6), 1358–1369.

Delfino, M., Iurino, D. A., Mercurio, B., Piras, P., Rook, L., & Sardella, R. (2020). Old African fossils provide new evidence for the origin of the American crocodiles. *Scientific Reports*, *10*, 11127.

Díaz Aráez, J. L., Delfino, M., Luján, À. H., Fortuny, J., Bernardini, F., & Alba, D. M. (2017). New remains of *Diplocynodon* (Crocodylia: Diplocynodontidae) from the Early Miocene of the Iberian Peninsula. *Comptes Rendus Palevol*, *16*(1), 12–26.

Dollo, L. (1883). Première note sur les crocodiliens de Bernissart. *Bulletin du Musée Royal d'Histoire Naturelle de Belgique*, *2*, 309–338.

Dollo, L. (1907). Nouvelle note sur les reptiles de l'Eocène inférieur de la Belgique et des régions voisines (*Eosuchus lerichei* et *Eosphargis gigas*). *Bulletin de la Société belge de Géologie, Paleontologie et Hydrologie*, *21*, 81–85.

Drumheller, S. K., Wilberg, E. W., & Sadleir, R. W. (2016). The utility of captive animals in actualistic research: a geometric morphometric exploration of the tooth row of *Alligator mississippiensis* suggesting ecophenotypic influences and functional constraints. *Journal of Morphology*, *277*(7), 866–878.

Dufeau, D. L., & Witmer, L. M. (2015). Ontogeny of the middle-ear air-sinus system in *Alligator mississippiensis* (Archosauria: Crocodylia). *PLoS ONE*, *10*(9), e0137060.

Duméril, M. C., & M. Aug. Duméril (1851). Catalogue méthodique de la collection des reptiles du Muséum d'Histoire Naturelle de Paris. Gide et Baudry, Paris, 224 pp.

Erickson, B. R. (1976). Osteology of the early eusuchian crocodile *Leidyosuchus formidabilis*, sp. nov. *Monographs of the Science Museum of Minnesota (Paleontology)*, *2*, 1–61.

Erickson, B. R. (1982). *Wannaganosuchus*, a new alligator from the Paleocene of North America. *Journal of Paleontology*, *56*(2), 492–506.

Falconer, H. (1859). *Descriptive catalogue of fossil remains of vertebrata from the Sewalik Hills, the Nerbudda, Perim Island, etc. in the Museum of the Asiatic Society of Bengal*. Baptist Mission Press, Calcutta, 261 pp.

Fauvel, A. A. (1879). Alligators in China: their history, description and identification. *Journal of the North-China Branch of the Royal Asiatic Society*, *1*, 1–36.

Figueiredo, R. G., Moreira, J. K. R., Saraiva, A. A. F., & Kellner, A. W. A. (2011). Description of a new specimen of *Susisuchus anatoceps* (Crocodylomorpha: Mesoeucrocodylia) from the Crato Formation (Santana Group) with comments on Neosuchia. *Zoological Journal of the Linnean Society*, *163*, S273–S288.

Foth, C., Bona, P., & Desojo, J. (2015). Intraspecific variation in the skull morphology of the black caiman *Melanosuchus niger* (Alligatoridae, Caimaninae). *Acta Zoologica*, *96*, 1–13.

Foth, C., Fernandez Blanco, M. V., Bona, P., & Scheyer, T. M. (2018). Cranial shape variation in jacarean caimanines (Crocodylia, Alligatoroidea) and its implications in the taxonomic status of extinct species: The case of *Melanosuchus fisheri*. *Journal of Morphology*, *279*(2), 259–273.

Fourtau, R. (1920). *Contribution à l'étude des vertébrés miocènes de l'Egypte*. Government Press.

Frey, E. (1988). Anatomie des Körperstammes von *Alligator mississippiensis* Daudin. *Stuttgarter Beiträge zur Naturkunde, Serie A (Biologie)*, *424*, 1–106.

Frey, E., & Salisbury, S. W. (2007). Crocodilians of the Crato Formation: evidence for enigmatic species. Pp. 463–476 in D. M. Martill, G. Bechly & R. F. Loveridge (eds.) *The Crato Fossil Beds of Brazil: Window Into an Ancient World*, Cambridge University Press, New York, U. S. A.

Frey, E., Laemmert, A., & Riess, J. (1987). *Baryphracta deponiae* n. g. n. sp. (Reptilia, Crocodylia), ein neues Krokodil aus der Grube Messel bei Darmstadt (Hessen, Bundesrepublik Deutschland). *Neues Jahrbuch für Geologie und Paläontologie, Monatschefte*, *1987*, 15–26.

Frey, E., Riess, J., & Tarsitano, S. F. (1989). The axial tail musculature of recent crocodiles and its phyletic implications. *American Zoologist*, *29*(3), 857–862.

Fuchs, K. (2006). *Crocodile Skin: Important Characteristics in Identifying Crocodilian Species*. Editions Chimaira, Frankfurt, 175 pp.

Gilmore, C. W. (1910). *Leidyosuchus sternbergii*, a new species of crocodile from the Cretaceous Beds of Wyoming. *Proceedings of the United States National Museum*, *38*, 485–502.

Gilmore, C. W. (1911). A new fossil alligator from the Hell Creek beds of Montana. *Proceedings of the U. S. National Museum*, *41*(1860), 297–301.

Gilmore, C. W. (1946). A new crocodilian from the Eocene of Utah. *Journal of Paleontology*, *20*(1), 62–67.

Ginsburg, L., & Buffetaut, E. (1978). *Euthecodon arambourgi* n. sp., et l'évolution du genre *Euthecodon*, Crocodilien du Néogène d'Afrique. *Géologie Méditerranéenne*, *5*(2), 291–301.

Gmelin, J. F. (1789). *Caroli a Linné systema naturae per regna tri naturae: secundum classes, ordines, genera, species, cum characteribus, differentiis, synonymis, locis. Tomus 1, Pars III. Editio decima tertia, aucta, reformata*, pp. 1057–1058. Georg Emanuel Beer, Leipzig.

Godoy, P. L., Cidade, G. M., Montefeltro, F. C., Langer, M. C., & Norell, M. A. (2021). Redescription and phylogenetic affinities of the caimanine *Eocaiman cavernensis* (Crocodylia, Alligatoroidea) from the Eocene of Argentina. *Papers in Palaeontology*, https://doi.org/10.1002/spp2.1339.

Gold, M. E. L. (2011). *Cranial osteology and braincase morphometrics of* Gavialis gangeticus*: implications for crocodylian phylogenetics*. Unpublished Master of Science thesis, University of Iowa, Iowa City, 103 pp.

Grandidier, A., & Vaillant, L. (1872). Sur le crocodile fossile d'Amboulintsatre (Madagascar). *Comptes Rendus de l'Academie des Sciences de Paris*, *75*, 150–151.

Gratten, J. (2003). The Molecular Systematics, Phylogeography and Population Genetics of Indo-Pacific *Crocodylus*. Unpublished PhD thesis, The University of Queensland, Brisbane, 222 pp.

Graves, M. L. (1819). Sur deux novelles espécies de crocodile. *Annales Générales des Sciences Physiques de Bruxelles*, *2*, 343–353.

Grigg, G., & Kirshner, D. (2015). *Biology and Evolution of Crocodylians*. CSIRO Publishing, Clayton South, 649 pp.

Hair, P. (1868). On the arrangement of the muscular fibers of the alligator. *Journal of Anatomy*, *2*(1), 26–41.

Hall, P. M., & Portier, K. M. (1994). Cranial morphometry of New Guinea crocodiles (*Crocodylus novaeguineae*): ontogenetic variation in relative growth of the skull and an assessment of its utility as a predictor of the sex and size of individuals. *Herpetological Monographs*, *8*, 203–225.

Harshman, J., Huddleston, C. J., Bollback, J. P., Parsons, T. J., & Braun, M. J. (2003). True and false gharials: a nuclear gene phylogeny of Crocodylia. *Systematic Biology*, *52*(3), 386–402.

Hastings, A. K., Bloch, J. I., Jaramillo, C. A., Rincon, A. F., & Macfadden, B. J. (2013). Systematics and biogeography of crocodylians from the Miocene of Panama. *Journal of Vertebrate Paleontology*, *33*(2), 239–263.

Hecht, M. K. (1987). Fossil snakes and crocodilians from the Sahabi Formation in Libya. Pp. 101–106 in N. T. Boaz, A. El-Arnauti, A. W. Gaziry, J. Heinzelin & D. D. Boaz (eds.) *Neogene paleontology and geology of Sahabi*, Alan R. Liss, New York.

Holliday, C. M., Porter, W. R., Vliet, K. A., & Witmer, L. M. (2020). The frontoparietal fossa and dorsotemporal fenestra of archosaurs and their significance for interpretations of vascular and muscular anatomy in dinosaurs. *The Anatomical Record*, *303*(4), 1060–1074.

Holt, T. R., Salisbury, S. W., & Willis, P. M. A. (2005). A new species of mekosuchine crocodilian from the middle Palaeogene Rundle Formation, central Queensland. *Memoirs of the Queensland Museum*, *50*(2), 207–218.

Hua, S. & Jouve, S. (2004). A primitive marine gavialoid from the Paleocene of Morocco. *Journal of Vertebrate Paleontology*, *24*(2), 341–350.

Hulke, J. W. (1878). Note on two skulls from the Wealden and the Purbeck Formations indicating a new subgroup of Crocodilia. *Quarterly Journal of the Geological Society of London*, *34*(1–4), 377–382.

Hutchinson, J. R. (2020, March 23). hatchling *Palaeosuchus palpebrosus* RVC-JRH-PP1 whole body. https://doi.org/10.17605/OSF.IO/UAD97

Iijima, M. (2017). Assessment of trophic ecomorphology in non-alligatoroid crocodylians and its adaptive and taxonomic implications. *Journal of Anatomy*, *231*(2), 192–211.

Iordansky, N. N. (1973). The skull of the Crocodilia. Pp. 201–262 in C. Gans & T. Parsons (eds.) *Biology of the Reptilia*, *4*, Academic Press, London.

de Iuliis, G. & Pulerà, D. (2011). Reptile skulls and mandibles. Pp. 253–285 in G. de Iuliis & D. Pulerà (eds.) *The Dissection of Vertebrates: A Laboratory Manual (Second Edition)*, Academic Press, Oxford.

Jiménez Fuentes, E. (1983). Algunos restos de craneos de cocodrilo del Paleógeno de Salamanca. *Stvdia Geologica*, *19*, 79–100.

Joffe, J. (1967). The 'dwarf' crocodiles of the Purbeck Formation, Dorset: a reappraisal. *Palaeontology*, *10*(4), 629–639.

Jouve, S. (2004). *Etude des Crocodyliformes fini Crétacé-Paléogène du Bassin des Oulad Abdoun (Maroc) et comparaison avec les faunes africaines contemporaines: systématique, phylogénie*

et paléobiogéographie. Unpublished PhD thesis, Muséum National d'Histoire Naturelle, Paris, 651 pp.

Jouve, S. (2016). A new basal tomistomine (Crocodylia, Crocodyloidea) from Issel (Middle Eocene; France): palaeobiogeography of basal tomistomines and palaeogeographic consequences. *Zoological Journal of the Linnean Society*, *177*(1), 165–182.

Jouve, S. (2017). *Leiokarinosuchus brookensis*, *Pholidosaurus meyeri* or *Anteophthalmosuchus hooleyi*? What is this croc? *Journal of Vertebrate Palaeontology*, *37*(2), e1297947.

Jouve, S., Bardet, N., Jalil, N.-E., Pereda Suberbiola, X., Bouya, B., & Amaghzaz, M. (2008). The oldest African crocodylian: phylogeny, paleobiogeography, and differential survivorship of marine reptiles through the Cretaceous-Tertiary boundary. *Journal of Vertebrate Paleontology*, *28*(2), 409–421.

Jouve, S., Bouya, B., Amaghzaz, M., & Meslouh, S. (2015). *Maroccosuchus zennaroi* (Crocodylia: Tomistominae) from the Eocene of Morocco: phylogenetic and palaeobiogeographical implications of the basalmost tomistomine. *Journal of Systematic Palaeontology*, *13*(5), 421–445.

Kälin, J. A. (1933). Beiträge zur vergleichenden Osteologie des Crocodilidenschädels. *Zoologische Jahrbücher*, *57*(4), 535–714.

Kälin, J. A. (1936). *Hispanochampsa mülleri* nov. gen. nov. spec., ein neuer Crocodilide aus dem unteren Oligocaen von Tárrega (Catalonien). *Abhandlungen Schweizerischen Palaeontologischen Gesellschaft*, *58*, 1–40.

Karl, H.-V., Gröning, E., Brauckmann, C., Schwarz, D., & Knötschke, N. (2006). The Late Jurassic crocodiles of the Langenberg near Oker, Lower Saxony (Germany), and description of related materials (with remarks on the history of quarrying the "Langenberg Limestone" and "Obernkirchen Sandstone"). *Clausthaler Geowissenschaften*, *5*, 59–77.

de Kay, J. E. (1842). *Zoology of New York*. White & Visscher, 415 pp.

Klein, G. F. (2016). *Skeletal anatomy of* Alligator *and comparison with* Thecachampsa. Calvert Marine Museum, Solomons, 70 pp.

Kley, N., Sertich, J., Turner, A., Krause, D., O'Connor, P., & Georgi, J. (2010). Craniofacial morphology of *Simosuchus clarki* (Crocodyliformes: Notosuchia) from the Late Cretaceous of Madagascar. *Journal of Vertebrate Paleontology*, *30*(S1), 13–98.

Kobatake, N., Chiji, M., Ikebe, N., Ishida, S., Kamei, T., Nakaseko, K., & Matsumoto, E. (1965). *Discovery of a crocodile fossil from the Osaka Group* [in Japanese]. *Quaternary Research* [= *Daiyonki Kenkyu*], *4*(2), 49–58.

Kobayashi, Y., Tomida, Y., Kamei, T., & Eguchi, T. (2006). Anatomy of a Japanese tomistomine crocodylian, *Toyotamaphimeia machikanensis* (Kamei et Matsumoto, 1965), from the middle Pleistocene of Osaka Prefecture: the reassessment of its phylogenetic status within Crocodylia. *National Science Museum Monographs*, *35*, 1–121.

Kraus, R. (1998). The cranium of *Piscogavialis jugaliperforatus* n. gen., n. sp. (Gavialidae, Crocodylia) from the Miocene of Peru. *Paläontologische Zeitschrift*, *72*(3–4), 389–406.

Krefft, G. (1873). Remarks on Australian crocodiles and description of a new species. *Proceedings of the Zoological Society of London*, *1873*, 334–335.

Kuhn, O. (1938). Die Crocodilier aus dem mittleren Eozän des Geiseltales bei Halle. *Nova Acta Leopoldina*, *39*, 313–328.

Lambe, L. M. (1907). On a new crocodilian genus and species from the Judith River Formation of Alberta. *Transactions of the Royal Society of Canada, series 3*, *4*, 219–244.

Langston, W. (1965). Fossil crocodilians from Colombia and the Cenozoic history of the Crocodilia in South America. *University of California Publications in Geological Science*, *52*, 1–157.

Langston, W. (1966). *Mourasuchus* Price, *Nettosuchus* Langston, and the family Nettosuchidae (Reptilia: Crocodilia). *Copeia*, *1966*(4), 882–885.

Langston, W. (1975). Ziphodont crocodiles: *Pristichampsus vorax* (Troxell), new combination, from the Eocene of North America. *Fieldiana: Geology*, *33*(16), 291–314.

Langston, W., & Gasparini, Z. (1997). Crocodilians, *Gryposuchus*, and the South American gavials. Pp. 113–154 in R. F. Kay, R. H. Madden, R. L. Cifelli & J. J. Flynn (eds.) *Vertebrate Paleontology in the Neotropics–The Miocene fauna of La Venta, Colombia*, Smithsonian Institution, Washington DC.

Laurenti, J. N. (1768). *Specimen medicum, exhibens synopsin reptilium emendatam cum experimentis circa venena et antidota reptilium austriacorum, quod authoritate et consensu*. Trattnern, Vienna, 217 pp.

Lee, M. S. Y., & Yates, A. M. (2018). Tip-dating and homoplasy: reconciling the shallow molecular divergences of modern gharials with their long fossil record. *Proceedings of the Royal Society B*, *285*(1881), 20181071.

Leidy, J. (1852). Description of a new species of crocodile from the Miocene of Virginia. *Journal of the Academy of Natural Sciences of Philadelphia*, *2*(2), 135–138.

Leite, K. J., & Fortier, D. C. (2018). The palate and choanae structure of the *Susisuchus anatoceps* (Crocodyliformes, Eusuchia): phylogenetic implications. *PeerJ*, *6*, e5372.

Lesson, R. P. (1831). Reptiles. Pp. 291–336, pls. 1-7 in I. G. S. Bélanger (ed.), *Voyage aux Indes-Orientales, par le nord de l'Europe, les provinces du Caucase, la Géorgie, l'Arménie, et la Perse, suivi de détails topographiques, statistiques et autres sur le Pégou, les iles de Java, de Maurice et de Bourbon, sur le cap-de-bonne-espérance et Sainte-Hélen, pendant les années 1825, 1826, 1827, 1828 et 1829. Zoologie*. A. Bertrand, Paris, xxxix + 535 pp.

Li, C., Wu, X.-c., & Rufolo, S. J. (2019). A new crocodyloid (Eusuchia: Crocodylia) from the upper cretaceous of China. *Cretaceous Research*, *94*, 25–39.

Li, J. (1975). New materials of *Tomistoma petrolica* from Maoming, Guangdong. *Vertebrata PalAsiatica*, *13*(3), 190–194.

Li, J. (1976). Fossil of Sebecosuchia discovered from Nanxiong, Guangdong. *Vertebrata PalAsiatica*, *14*(3), 169–173.

Li, J. (1984). A new species of *Planocrania* from Hengdong, Hunan. *Vertebrata PalAsiatica*, *22*(2), 123–133.

Linnaeus, C. (1758). *Systema Naturae per regna tria naturae, secundum classes, ordines, genera, species, cum characteribus, differentiis, synonymis, locis, Tomus I. Editio decima, reformata*. Holmiae, impensis direct. Laurentii Salvii, 1–824.

Loomis, F. B. (1904). Two new river reptiles from the Titanothere beds. *American Journal of Science (ser. 4)*, *18*, 427–432.

Ludwig, R. (1877). Fossile Crocodiliden aus der Tertiärformation des mainzer Beckens. *Palaeontographica Suppl. 3*, 1–52.

Lull, R. S. (1944). Fossil gavials from north India. *American Journal of Science*, *242*(8), 417–430.

Maccagno, A. M. (1948). Descrizione di una nuova specie di "*Crocodilus*" del giacimento di Sahabi (Sirtica). *Atti della Reale Accademia Nazionale dei Lincei: Memorie della Classe di Scienze fisiche, Mathematiche e Naturale, Serie 8*, *1*(2), 63–96.

Marsh, O. C. (1870). Notice of a new species of gavial from the Eocene of New Jersey. *American Journal of Science*, *148*, 97–99.

Marsh, O. C. (1871). Notice of some new fossil reptiles from the Cretaceous and Tertiary formations. *American Journal of Science and Arts (ser. 3) 1*, *6*, 447–459.

Martin, J. E. (2007). New material of the Late Cretaceous globidontan *Acynodon iberoccitanus* (Crocodylia) from southern France. *Journal of Vertebrate Paleontology*, *27*(2), 362–372.

Martin, J. E. (2010). A new species of *Diplocynodon* (Crocodylia, Alligatoroidea) from the Late Eocene of the Massif Central, France, and the evolution of the genus in the climatic context of the Late Palaeogene. *Geological Magazine*, *147*(4), 596–610.

Martin, J. E., & Lauprasert, K. (2010). A new primitive alligatorine from the Eocene of Thailand: relevance of Asiatic members to the radiation of the group. *Zoological Journal of the Linnean Society*, *158*(3), 608–628.

Martin, J. E., & Gross, M. (2011). Taxonomic clarification of *Diplocynodon* Pomel, 1847 (Crocodilia) from the Miocene of Styria, Austria. *Neues Jahrbuch für Geologie und Paläontologie-Abhandlungen*, *261*, 177–193.

Martin, J. E., Smith, T., de Lapparent de Broin, F., Escuillié, F., & Delfino, M. (2014). Late Palaeocene eusuchian remains from Mont de Berru, France, and the origin of the alligatoroid Diplocynodon. *Zoological Journal of the Linnean Society*, *172*(4), 867–891.

Martin, J. E., Delfino, M., & Smith, T. (2016). Osteology and affinities of Dollo's goniopholidid (Mesoeucrocodylia) from the Early Cretaceous of Bernissart, Belgium. *Journal of Vertebrate Paleontology*, *36*(6), e1222534.

Martin, J. E., Smith, T., Salaviale, C., Adrien, J., & Delfino, M. (2020). Virtual reconstruction of the skull of *Bernissartia fagesii* and current understanding of the neosuchian–eusuchian transition. *Journal of Systematic Palaeontology*, *18*(13), 1079–1101.

Massonne, T., Vasilyan, D., Rabi, M., & Böhme, M. (2019). A new alligatoroid from the Eocene of Vietnam highlights an extinct Asian clade independent from extant *Alligator sinensis*. *PeerJ*, *7*, e7562.

Mateus, O., Puértolas-Pascual, E., & Callapez, P. M. (2019). A new eusuchian crocodylomorph from the Cenomanian (Late Cretaceous) of Portugal reveals novel implications on the origin of Crocodylia. *Zoological Journal of the Linnean Society*, *186*(2), 501–528.

Medina, C. J. (1976). Crocodilian from the Late Tertiary of Northwestern Venezuela: *Melanosuchus fisheri* sp. nov. *Breviora*, *438*, 1–14.

Megirian, D. (1994). A new species of *Quinkana* Molnar (Eusuchia: Crocodylidae) from the Miocene Camfield Beds of northern Australia. *The Beagle, Records of the Museums and Art Galleries of Northern Territory*, *11*, 145–166.

Megirian, D., Murray, P. F., & Willis, P. (1991). A new crocodile of the gavial ecomorph morphology from the Miocene of northern Australia. *The Beagle, Records of the Northern Territory Museum of Arts and Sciences*, *8*(1), 135–158.

Meyer, H. von. (1832). *Paleologica zur Geshichte der Erde und irher Geschöpfe*. S. Schmerber, Frankurt-am-Main, 560 pp.

Molnar, R. E. (1981). Pleistocene ziphodont crocodilians of Queensland. *Records of the Australian Museum*, *33*(19), 803–834.

Molnar, R. E. (1982). *Pallimnarchus* and other Cenozoic crocodiles in Queensland. *Memoirs of the Queensland Museum*, *20*(3), 657–673.

Montefeltro, F. C., Andrade, D. V., & Larsson, H. C. (2016). The evolution of the meatal chamber in crocodyliforms. *Journal of Anatomy*, *228*(5), 838–863.

Mook, C. C. (1921a). Description of a skull of a Bridger crocodilian. *Bulletin of the American Museum of Natural History*, *44*(11), 111–116.

Mook, C. C. (1921b). The skull of *Crocodilus acer* Cope. *Bulletin of the American Museum of Natural History*, *44*(11), 117–121.

Mook, C. C. (1921c). Skull characters of recent Crocodilia, with notes on the affinities of the recent genera. *Bulletin of the American Museum of Natural History*, *44*(11), 123–268.

Mook, C. C. (1923a). A new species of alligator from the Snake Creek Beds. *American Museum Novitates*, *73*, 1–13.

Mook, C. C. (1923b). Skull characters of *Alligator sinense* [sic.] Fauvel. *Bulletin of the American Museum of Natural History*, *48*, 553–562.

Mook, C. C. (1924a). A new crocodilian from Mongolia. *American Museum Novitates*, *117*, 1–5.

Mook, C. C. (1924b). A new crocodilian from the Wasatch Beds. *American Museum Novitates*, *137*, 1–4.

Mook, C. C. (1927). The skull characters of *Crocodilus megarhinus* Andrews. *American Museum Novitates*, *289*, 1–8.

Mook, C. C. (1932). A study of the osteology of *Alligator prenasalis* (Loomis). *Bulletin of the Museum of Comparative Zoology*, *74*(2), 19–41.

Mook, C. C. (1933). A skull with jaws of *Crocodilus sivalensis* Lydekker. *American Museum Novitates*, *670*, 1–10.

Mook, C. C. (1941a). A new crocodilian from the Lance Formation. *American Museum Novitates*, *1128*, 1–5.

Mook, C. C. (1941b). A new crocodilian, *Hassiacosuchus kayi*, from the Bridger Eocene beds of Wyoming. *Annals of the Carnegie Museum*, *28*, 207–220.

Mook, C. C. (1941c). A new fossil crocodilian from Colombia. *Proceedings of the United States National Museum*, *91*(3122), 55–58.

Mook, C. C. (1946). A new Pliocene alligator from Nebraska. *American Museum Novitates*, *1311*, 1–12.

Mook, C. C. (1959). A new species of fossil crocodile of the genus *Leidyosuchus* from the Green River beds. *American Museum Novitates*, *1933*, 1–6.

Mook, C. C. (1961). Notes on the skull characters of *Allognathosuchus polyodon*. *American Museum Novitates*, *2072*, 1–5.

Morgan, G. S., & Albury, N. A. (2013). The Cuban Crocodile (*Crocodylus rhombifer*) from late Quaternary fossil deposits in the Bahamas and Cayman islands. *Bulletin of the Florida Museum of Natural History*, *52*(3), 162–236.

Morgan, G. S., Albury, N. A., Rímoli, R., Lehman, P., Rosenberger, A. L., & Cooke, S. B. (2018). The Cuban Crocodile (*Crocodylus rhombifer*) from late Quaternary underwater cave deposits in the Dominican Republic. *American Museum Novitates*, *3916*, 1–56.

Morgan, G. S., Franz, R., & Crombie, R. I. (1993). The Cuban Crocodile, *Crocodylus rhombifer*, from Late Quaternary Fossil Deposits on Grand Cayman. *Caribbean Journal of Science*, *29*(3–4), 153–164.

Müller, L. (1927). Ergebnisse der Forschungsreisen Prof. E. Stromers in den Wüsten Ägyptens. V. Tertiäre Wirbeltiere: 1. Beiträge zur Kenntnis der Krokodilier des ägyptischen Tertiärs. *Abhandlungen der Bayerischen Akademie der Wissenschaften, Mathematisch - naturwissenschaftliche Abteilung*, *31*(2), 1–97.

Müller, S. (1838). Waarnemingen over de Indische krokodillen en Beschrijving van eene nieuwe soort. *Tijdschrift voor Natuurlijke Geschiedenis en Physiologie. Amsterdam and Leyden*, *5*, 1–27.

Müller, S. (1846). Ueber den Charakter der Thierwelt auf den Inseln des indischen Archipels, ein Beitrag zur zoologischen Geographie. *Archiv für Naturgeschichte*, *12*, 109–128.

Müller, S., & Schlegel, H. (1844). Over de Krokodillen van den Indischen Archipel. P. 28 in C. J. Temminck, 1839-1844. *Verhandelingen over de natuurlijke geschiedenis der Nederlandsche overzeesche bezittingen, door de leden der Natuurkundige Commissie in Indie en andere Schrijvers*. Leiden, 259 pp.

Myrick, A. C. (2001). *Thecachampsa antiqua* (Leidy, 1852) (Crocodylidae: Thoracosaurinae) from fossil marine deposits at Lee Creek Mine, Aurora, North Carolina, USA. *Smithsonian Contributions to Paleobiology*, *90*, 219–225.

Narváez, I., Brochu, C. A., Escaso, F., Pérez-García, A., & Ortega, F. (2015). New crocodyliforms from southwestern Europe and definition of a diverse clade of European Late Cretaceous basal eusuchians. *PLoS ONE*, *10*(11), e0140679.

Narváez, I., Brochu, C. A., Escaso, F., Pérez-García, A., & Ortega, F. (2016). New Spanish Late Cretaceous eusuchian reveals the synchronic and sympatric presence of two allodaposuchids. *Cretaceous Research*, *65*, 112–125.

Narváez, I., Brochu, C. A., De Celiz, A., Codrea, V., Escaso, F., Pérez-García, A., & Ortega, F. (2020). New diagnosis for *Allodaposuchus precedens*, the type species of the European Upper Cretaceous clade Allodaposuchidae. *Zoological Journal of the Linnean Society*, *189*(2), 618–634.

Nopcsa, F. (1928). Paleontological notes on Reptilia. 7. Classification of the Crocodilia. *Geologica Hungarica, Series Palaeontologica*, *1*(1), 75–84.

Norell, M. A. (1988). *Cladistic approaches to paleobiology as applied to the phylogeny of alligatorids*. Unpublished PhD thesis, Yale University, New Haven, 279 pp.

Norell, M. A. (1989). The higher level relationships of the extant Crocodylia. *Journal of Herpetology*, *23*(4), 325–335.

Norell, M. A., & Clark, J. M. (1990). A reanalysis of *Bernissartia fagesii*, with comments on its phylogenetic position and its bearing on the origin and diagnosis of the Eusuchia. *Bulletin de l'Institut Royal des Sciences Naturelles de Belgique*, *60*, 115–128.

Norell, M. A., Clark, J. M., & Hutchison, J. H. (1994). The Late Cretaceous alligatoroid *Brachychampsa montana* (Crocodylia): new material and putative relationships. *American Museum Novitates*, *3116*, 1–26.

Norell, M. A., & Storrs, G. W. (1989). Catalogue and review of the type fossil crocodilians in the Yale Peabody Museum. *Postilla*, *203*, 1–28.

Oppel, M. (1811). *Die Ordnung, Familien und Gattungen der Reptilien als Prodrom einer Naturgeschichte derselben*. Lindauer, Munich, 87 pp.

Ősi, A. (2008). Cranial osteology of *Iharkutosuchus makadii*, a Late Cretaceous basal eusuchian crocodyliform from Hungary. *Neues Jahrbuch für Geologie und Paläontologie-Abhandlungen*, *248*(3), 279–299.

Ősi, A. (2014). The evolution of jaw mechanism and dental function in heterodont crocodyliforms. *Historical Biology: An International Journal of Paleobiology*, *26*(3), 279–414.

Ősi, A., & Weishampel, D. B. (2009). Jaw mechanism and dental function in the Late Cretaceous basal eusuchian *Iharkutosuchus*. *Journal of Morphology*, *270*(8), 903–920.

Ősi, A., Clark, J. M., & Weishampel, D. B. (2007). First report on a new basal eusuchian crocodyliform with multicusped teeth from the Upper Cretaceous (Santonian) of Hungary. *Neues Jahrbuch für Geologie und Paläontologie-Abhandlungen*, *243*(2), 169–177.

Owen, R. (1841). On British fossil reptiles. *Report of the British Association for the Advancement of Science*, *11*, 60–204.

Owen, R. (1874). Monograph on the fossil Reptilia of the Wealden and Purbeck Formations. Supplement No. IV (*Hylaeochampsa*). *Palaeontolographical Society, Monographs*, *27*, 1–7.

Owen, R. (1878a). Monograph on the fossil Reptilia of the Wealden and Purbeck Formations. Supplement No. VIII. Crocodilia (*Goniopholis*, *Pterosuchus*, and *Suchosaurus*). *Palaeontolographical Society, Monographs*, *32*, 1–15.

Owen, R. (1878b). On the fossils called "granicones"; being a contribution to the histology of the exo-skeleton in" Reptilia". *Journal of the Royal Microscopical Society*, *1*, 233–236.

Owen, R. (1879). Monograph on the fossil Reptilia of the Wealden and Purbeck Formations. Supplement No. IX. Crocodilia (*Goniopholis*, *Brachydectes*, *Nannosuchus*, *Theriosuchus* and *Nuthetes*). *Palaeontographical Society, Monographs*, *33*, 1–19.

Pickford, M. (2003). A new species of crocodile from early and middle Miocene deposits of the lower Orange River Valley, Namibia, and the origins of the Nile Crocodile (*Crocodylus niloticus*). *Geological Survey of Namibia Memoir*, *19*(2003), 51–65.

Pierce, S. E., Williams, M., & Benson, R. B. J. (2017). Virtual reconstruction of the endocranial anatomy of the early Jurassic marine crocodylomorph *Pelagosaurus typus* (Thalattosuchia). *PeerJ*, *5*, e3225.

Piras, P., & Buscalioni, A. D. (2006). *Diplocynodon muelleri* comb. nov., an Oligocene diplocynodontine alligatoroid from Catalonia (Ebro Basin, Lleida Province, Spain). *Journal of Vertebrate Paleontology*, *26*(3), 608–620.

Piras, P., Delfino, M., Del Favero, L., & Kotsakis, T. (2007). Phylogenetic position of the crocodylian *Megadontosuchus arduini* and tomistomine palaeobiogeography. *Acta Palaeontologica Polonica*, *52*(2), 315–328.

Platt, S. G., Rainwater, T. R., Thorbjarnarson, J. B., Finger, A. G., Anderson, T. A., & McMurry, S. T. (2009). Size estimation, morphometrics, sex ratio, sexual size dimorphism, and biomass of Morelet's crocodile in northern Belize. *Caribbean Journal of Science*, *45*(1), 80–93.

Poe, S. (1996). Data set incongruence and the phylogeny of crocodilians. *Systematic Biology*, *45*(4), 393–414.

Pol, D., & Norell, M. A. (2004). A new gobiosuchid crocodyliform taxon from the Cretaceous of Mongolia. *American Museum Novitates*, *3458*, 1–31.

Pol, D., Turner, A. H., & Norell, M. A. (2009). Morphology of the Late cretaceous crocodylomorph *Shamosuchus djadochtaensis* and a discussion of neosuchian phylogeny as related to the origin of Eusuchia. *Bulletin of the American Museum of Natural History*, *324*, 1–103.

Pomel, A. (1847). Note sur les animaux fossiles découverts dans le département de l'Allier. *Bulletin de la Societé Geologique de France, serie 2*, *4*, 378–385.

Porter, W. R., Sedlmayr, J. C., & Witmer, L. M. (2016). Vascular patterns in the heads of crocodilians: blood vessels and sites of thermal exchange. *Journal of Anatomy*, *229*(6), 800–824.

Puértolas-Pascual, E., Canudo, J. I., & Cruzado-Caballero, P. (2011). A new crocodylian from the late Maastrichtian of Spain: implications for the initial radiation of crocodyloids. *PLoS ONE*, *6*(6), e20011.

Puértolas-Pascual, E., Canudo, J. I., & Moreno-Azanza, M. (2014). The eusuchian crocodylomorph *Allodaposuchus subjuniperus* sp. nov., a new species from the latest Cretaceous (Upper Maastrichtian) of Spain. *Historical Biology: An International Journal of Paleobiology*, *26*(1), 91–109.

Richardson, K. C., Webb, G. J. W., & Manolis, S. C. (2002). *Crocodiles: Inside Out*. Surrey Beatty & Sons, Sydney, 172 pp.

Rio, J. P., Mannion, P. D., Tschopp, E., Martin, J. E., & Delfino, M. (2020). Reappraisal of the morphology and phylogenetic relationships of the alligatoroid crocodylian *Diplocynodon*

hantoniensis from the late Eocene of the United Kingdom. *Zoological Journal of the Linnean Society*, *188*(2), 579–629.

Ristevski, J., Young, M. T., de Andrade, M. B., & Hastings, A. K. (2018). A new species of *Anteophthalmosuchus* (Crocodylomorpha, Goniopholididae) from the Lower Cretaceous of the Isle of Wight, United Kingdom, and a review of the genus. *Cretaceous Research*, *84*, 340–383.

Ristevski, J., Yates, A. M., Price, G. J., Molnar, R. E., Weisbecker, V., & Salisbury, S. W. (2020a). Australia's prehistoric 'swamp king': revision of the Plio-Pleistocene crocodylian genus *Pallimnarchus* de Vis, 1886. *PeerJ*, *8*, e10466.

Ristevski, J., Yates, A. M., Price, G. J., Molnar, R. E., Weisbecker, V., & Salisbury, S. W. (2020b). Data from: Australia's prehistoric 'swamp king': revision of the Plio-Pleistocene crocodylian genus *Pallimnarchus* de Vis, 1886. *Dryad, Dataset*. https://doi.org/10.5061/dryad.8kprr4xkq

Rogers, J. V. II. (2003). *Pachycheilosuchus trinquei*, a new procoelous crocodyliform from the Lower Cretaceous (Albian) Glen Rose Formation of Texas. *Journal of Vertebrate Paleontology*, *23*(1), 128–145.

Ross, C. A. (1990). *Crocodylus raninus* S. Müller and Schlegel, a valid species of crocodile (Reptilia: Crocodylidae) from Borneo. *Proceedings of the Biological Society of Washington*, *103*(4), 955–961.

Rossmann, T. (2000). Skelettanatomische Beschreibung von *Pristichampsus rollinatii* (Gray) (Crocodilia, Eusuchia) aus dem Paläogen von Europa, Nordamerika und Ostasien. *Courier Forschungsinstitut Senckenberg*, *221*, 1–107.

Rovereto, C. (1912). Los cocodrilos fósiles en las capas de Paraná. *Anales de Museo Nacional de Buenos Aires ser. 3*, *22*, 339–369.

Salas-Gismondi, R., Flynn, J. J., Baby, P., Tejada-Lara, J. V., Claude, J., & Antoine, P.-O. (2016). A new 13 million year old gavialoid crocodylian from proto-Amazonian mega-wetlands

reveals parallel evolutionary trends in skull shape linked to longirostry. *PLoS ONE*, *11*(4), e0152453.

Salisbury, S. W. (2001). *A biomechanical transformation model for the evolution of the eusuchian-type bracing system*. Unpublished PhD thesis, University of New South Wales, Sydney, 554 pp.

Salisbury, S. W. (2002). Crocodilians from the Lower Cretaceous (Berriasian) Purbeck Limestone Group of Dorset, Southern England. *Special Papers in Palaeontology*, *68*, 121–144.

Salisbury, S. W., & Naish, D. (2011). Crocodilians. Pp. 305–369 in D. J. Batten DJ (ed.) *English Wealden Fossils*, The Palaeontological Association, London.

Salisbury, S. W., & Willis, P. M. A. (1996). A new crocodylian from the Early Eocene of south-eastern Queensland and a preliminary investigation of the phylogenetic relationships of crocodyloids. *Alcheringa: An Australasian Journal of Palaeontology*, *20*(3),179–226.

Salisbury, S. W., Frey, E., Martill, D. M., & Buchy, M. C. (2003). A new crocodilian from the Lower Cretaceous Crato formation of northeastern Brazil. *Paläontographica*, *270*(1), 3–47.

Salisbury, S. W., Molnar, R. E., Frey, E., & Willis, P. M. A. (2006). The origin of modern crocodyliforms: new evidence from the Cretaceous of Australia. *Proceedings of the Royal Society B: Biological Sciences*, *273*(1600), 2439–2448.

Salisbury, S. W., Willis, P. M. A., Peitz, S., & Sander, P. M. (1999). The crocodilian *Goniopholis simus* from the Lower Cretaceous of north-western Germany. *Special Papers in Palaeontology*, *60*, 121–148.

Scheyer, T. M., Aguilera, O. A., Delfino, M., Fortier, D. C., Carlini, A. A., Sánchez, R., Carrillo-Briceño, J. D., Quiroz, L., & Sánchez-Villagra, M. R. (2013). Crocodylian diversity peak and extinction in the late Cenozoic of the northern Neotropics. *Nature Communications*, *4*, 1907.

Schmidt, K. P. (1919). Contributions to the herpetology of the Belgian Congo based on the collection of the American Museum Congo expedition, 1909–1915. Part 1. Turtles,

crocodiles, lizards and chameleons. *Bulletin of the American Museum of Natural History*, *39*(2), 385–624.

Schmidt, K. P. (1928). A new crocodile from New Guinea. *Zoological Series of the Field Museum of Natural History*, *12*(14), 175–181.

Schmidt, K. P. (1935). A new crocodile from the Philippine Islands. *Zoological Series of the Field Museum of Natural History*, *20*(8), 67–70.

Schmidt, K. P. (1938). New crocodilians from the upper Paleocene of western Colorado. *Geological Series of the Field Museum of Natural History*, *6*(21), 315–321.

Schmidt, K. P. (1941). A new fossil alligator from Nebraska. *Fieldiana: Geology*, *8*, 27–32.

Schneider, J. G. (1801). *Historiae amphibiorum naturalis et literariae. Fasciculus secundus continens Crocodilos, Scincos, Chamaesauras, Boas. Pseudoboas, Elapes, Angues, Amphisbaenas et Caecilias*. Friedrich Frommann, Jena, 365 pp.

Schwarz, D. (2002). A new species of *Goniopholis* from the Upper Jurassic of Portugal. *Palaeontology*, *45*(1), 185–208.

Schwarz, D., & Salisbury, S. W. (2005). A new species of *Theriosuchus* (Atoposauridae, Crocodylomorpha) from the Late Jurassic (Kimmeridgian) of Guimarota, Portugal. *Geobios*, *38*(6), 779–802.

Schwarz, D., Raddatz, M., & Wings, O. (2017). *Knoetschkesuchus langenbergensis* gen. nov. sp. nov., a new atoposaurid crocodyliform from the Upper Jurassic Langenberg Quarry (Lower Saxony, northwestern Germany), and its relationships to *Theriosuchus*. *PLoS ONE*, *12*(2), e0160617.

Serrano-Martínez, A., Knoll, F., Narváez, I., Lautenschlager, S., & Ortega, F. (2019a). Brain and pneumatic cavities of the braincase of the basal alligatoroid *Diplocynodon tormis* (Eocene, Spain). *Journal of Vertebrate Paleontology*, *39*(1), e1572612.

Serrano-Martínez, A., Knoll, F., Narváez, I., Lautenschlager, S., & Ortega, F. (2019b). Inner skull cavities of the basal eusuchian *Lohuecosuchus megadontos* (Upper Cretaceous, Spain) and neurosensorial implications. *Cretaceous Research*, *93*, 66–77.

Serrano-Martínez, A., Knoll, F., Narváez, I., Lautenschlager, S., & Ortega, F. (2020). Neuroanatomical and neurosensorial analysis of the Late Cretaceous basal eusuchian *Agaresuchus fontisensis* (Cuenca, Spain). *Papers in Palaeontology*, DOI: 10.1002/spp2.1296.

Shan, H.-y., Wu, X.-c., Cheng, Y.-n., & Sato, T. (2009). A new tomistomine (Crocodylia) from the Miocene of Taiwan. *Canadian Journal of Earth Sciences*, *46*(7), 529–555.

Shan, H.-Y., Wu, X.-C., Cheng, Y.-N., & Sato, T. (2017). *Maomingosuchus petrolica*, a restudy of '*Tomistoma*' *petrolica* Yeh, 1958. *Palaeoworld*, *26*(4), 672–690.

Simpson, G. G. (1930). *Allognathosuchus mooki*, a new crocodile from the Puerco Formation. *American Museum Novitates*, *445*, 1–16.

Simpson, G. G. (1933). A new crocodilian from the *Notostylops* beds of Patagonia. *American Museum Novitates*, *623*, 1–9.

Sookias, R. B. (2019, October 30). Exploring the effects of character construction and choice, outgroups, and analytical method on phylogenetic inference from discrete characters in extant crocodilians. https://doi.org/10.17605/OSF.IO/MGH48

Sookias, R. B. (2020). Exploring the effects of character construction and choice, outgroups and analytical method on phylogenetic inference from discrete characters in extant crocodilians. *Zoological Journal of the Linnean Society*, *189*(2), 670–699.

Spix, J. B. (1825). *Animalia nova sive species novae lacertarum quas in itinere per Brasiliam annis MDCCCXVII–MDCCCXX jussu et auspiciis Maximiliani Josephi I. Bavariae Regis suscepto collegit et descripsit Dr. J. B. de Spix*. T. O. Weigel, Leipzig, 26 pp.

de Stefano, G. (1905). Appunti sui Batraci e rettili del Quercy appartenenti alla collezioni Rossignol. *Bolletino della Societa Geologia Italiana*, *24*, 17–67.

Sternberg, C. M. (1932). A new fossil crocodile from Saskatchewan. *The Canadian Field-Naturalist*, *44*, 128–133.

Storrs, G. W. (2003). Late Miocene–Early Pliocene crocodilian fauna of Lothagam, southwest Turkana Basin, Kenya. Pp. 137–159 in M. G. Leakey & J. M. Harris (eds.) *Lothagam: The Dawn of Humanity in Eastern Africa*. Columbia University Press, New York, U. S. A.

Sullivan, R. M., & Lucas, S. G. (2003). *Brachychampsa montana* Gilmore (Crocodylia, Alligatoroidea) from the Kirtland Formation (Upper Campanian), San Juan Basin, New Mexico. *Journal of Vertebrate Paleontology*, *23*(4), 832–841.

Syme, C. E., & Salisbury, S. W. (2018). Taphonomy of *Isisfordia duncani* specimens from the Lower Cretaceous (upper Albian) portion of the Winton Formation, Isisford, central-west Queensland. *Royal Society Open Science*, *5*(3), 171651.

Tarsitano, S. F., Frey, E., & Riess, J. (1989). The evolution of the Crocodilia: a conflict between morphological and biochemical data. *American Zoologist*, *29*(3), 843–856.

Tchernov, E., & van Couvering, J. (1978). New crocodiles from the early Miocene of Kenya. *Palaeontology*, *21*(4), 857–867.

Tennant, J. P., Mannion, P. D., & Upchurch, P. (2016). Evolutionary relationships and systematics of Atoposauridae (Crocodylomorpha: Neosuchia): implications for the rise of Eusuchia. *Zoological Journal of the Linnean Society*, *177*(4), 854–936.

Toula, F., & Kail, J. A. (1885). Über einen Krokodil–Schädel aus den Tertiärablagerungen von Eggenburg in Niederösterreich: eine paläontologische studie. *Denkschriften der Kaiserlichen Akademie der Wissenschaften von Wien, Mathematisch–naturwissenschaftliche Classe*, *50*, 299–355.

Troxell, E. L. (1925). The Bridger crocodiles. *American Journal of Science*, 5th Ser., *9*, 29–72.

Turner, A. H. (2015). A review of *Shamosuchus* and *Paralligator* (Crocodyliformes, Neosuchia) from the Cretaceous of Asia. *PLoS ONE*, *10*(2), e0118116.

Wang Y.-y., Sullivan, C., & Liu, J. (2016). Taxonomic revision of *Eoalligator* (Crocodylia, Brevirostres) and the paleogeographic origins of the Chinese alligatoroids. *PeerJ*, *4*(5562), e2356.

Weitzel, K. (1935). *Hassiacosuchus haupti* n.g. n.sp., ein durophages Krokodil aus dem Mitteleozän von Messel. *Notizblatt des Vereins für Erdkunde und der Hessischen Geologischen Landesanstalt Darmstadt*, *16*, 40–49.

Wermuth, H. (1953). Systematik der rezenten Krokodile. *Mitteilungen aus dem Museum für Naturkunde in Berlin. Zoologisches Museum und Institut für Spezielle Zoologie (Berlin)*, *29*(2), 375–511.

White, T. E. (1942). A new alligator from the Miocene of Florida. *Copeia*, *1*, 3–7.

Whiting, E. T., & Hastings, A. K. (2015). First fossil *Alligator* from the late Eocene of Nebraska and the Late Paleogene record of alligators in the Great Plains. *Journal of Herpetology*, *49*(4), 560–569.

Williamson, T. E. (1996). ?*Brachychampsa sealeyi*, sp. nov., (Crocodylia, Alligatoroidea) from the Upper Cretaceous (lower Campanian) Menefee Formation, northwestern New Mexico. *Journal of Vertebrate Paleontology*, *16*(3), 421–431.

Willis, P. M. A. (1993). *Trilophosuchus rackhami* gen. et sp. nov., a new crocodilian from the Early Miocene limestones of Riversleigh, northwestern Queensland. *Journal of Vertebrate Paleontology*, *13*(1), 90–98.

Willis, P. M. A. (1997). New crocodilians from the late Oligocene White Hunter Site, Riversleigh, northwestern Queensland. *Memoirs of the Queensland Museum*, *41*(2), 423–438.

Willis, P. M. A. (2001). New crocodilian material from the Miocene of Riversleigh (northwestern Queensland, Australia). Pp. 64–74 in G. Grigg, F. Seebacher & C. E. Franklin (eds.) *Crocodilian Biology and Evolution*, Surrey Beatty & Sons, Sydney, Australia.

Willis, P. M. A., & Mackness, B. S. (1996). *Quinkana babarra*, a new species of ziphodont mekosuchine crocodile from the early Pliocene Bluff Downs local fauna, northern Australia

with a revision of the genus. *Proceedings of the Linnean Society of New South Wales*, *116*, 143–151.

Willis, P. M. A., & Molnar, R. E. (1991). A new Middle Tertiary crocodile from Lake Palankarinna, South Australia. *Records of the South Australian Museum*, *25*(1), 39–55.

Willis, P. M. A., Molnar, R. E., & Scanlon, J. D. (1993). An early Eocene crocodilian from Murgon, Southeastern Queensland. *Kaupia*, *3*, 27–33.

Willis, P., Murray, P., & Megirian, D. (1990). *Baru darrowi* gen. et sp. nov., a large broad-snouted crocodyline (Eusuchia: Crocodilidae) from mid-Tertiary freshwater limestones in northern Australia. *Memoirs of the Queensland Museum*, *29*(2), 521–540.

Willis, P. M. A., Robinson, J., & Kemp, A. (1995). Computerised tomographic scans of an Eocene crocodile skull from southeast Queensland. *Memoirs of the Association of Australasian Palaeontologists*, *18*, 203–208.

Witmer, L. M. (1997). The evolution of the antorbital cavity of archosaurs: a study in soft-tissue reconstruction in the fossil record with an analysis of the function of pneumaticity. *Journal of Vertebrate Paleontology*, *17*(S1), 1–73.

Wood, S. V. (1846). On the discovery of an alligator and of several new Mammalia in the Hordwell Cliff; with observations upon the geological phenomena of that locality. *London Geological Journal*, *1*, 117–122.

Wu, X. C., & Sues H.-D. (1996). Anatomy and phylogenetic relationships of *Chimaerasuchus paradoxus*, an unusual crocodyliform reptile from the Lower Cretaceous of Hubei, China. *Journal of Vertebrate Paleontology*, *16*(4), 688–702.

Wu, X.-C., Brinkman, D. B., & Russell, A. P. (1996). A new alligator from the Upper Cretaceous of Canada and the relationship of early eusuchians. *Palaeontology*, *39*(2), 351–375.

Wu, X.-C., Russell, A. P., & Brinkman, D. B. (2001). A review of *Leidyosuchus canadensis* Lambe, 1907 (Archosauria: Crocodylia) and an assessment of cranial variation based upon new material. *Canadian Journal of Earth Sciences*, *38*, 1665–1687.

Yates, A. M. (2017). The biochronology and palaeobiogeography of *Baru* (Crocodylia: Mekosuchinae) based on new specimens from the Northern Territory and Queensland, Australia. *PeerJ*, *5*, e3458.

Yates, A. M., & Pledge, N. S. (2016). A Pliocene mekosuchine (Eusuchia: Crocodilia) from the Lake Eyre Basin of South Australia. *Journal of Vertebrate Paleontology*, *37*(1), e1244540.

Yeh, H. (1958). A new crocodile from Maoming, Kwangtung. *Vertebrata PalAsiatica*, *2*(4), 237–242.

Young, C. C. (1964). New fossil crocodiles from China. *Vertebrata Palasiatica*, *8*(2), 189–208.

Young, C. C. (1982). *A Cenozoic crocodile from Huaining, Anhui. Selected works of Yang Zhongjian*. China: Academia Sinica, 47–48.

Young, M. T. (2014). Filling the 'Corallian Gap': re-description of a metriorhynchid crocodylomorph from the Oxfordian (Late Jurassic) of Headington, England. *Historical Biology: An International Journal of Paleobiology*, *26*(1), 80–90.

Young, B. A., & Bierman, H. S. (2019). On the median pharyngeal valve of the American alligator (*Alligator mississippiensis*). *Journal of Morphology*, *280*(1), 58–67.

Zangerl, R. (1944). *Brachyuranochampsa eversolei*, gen. et sp. nov., a new crocodilian from the Washakie Eocene of Wyoming. *Annals of the Carnegie Museum*, *30*, 77–84.

de Zigno, A. (1880). Sopra un cranio di coccodrillo scoperto nel terreno eoceno del Veronese. *Atti della Reale Accademia Lincei, Memorie della Classe di Scienze Fisiche, Matematiche e Naturali, Serie 3*, *5*, 65–72.
