# Supplementary material for: First record of a tomistomine crocodylian from Australia
Source: Sci Rep. 2021 Jun 9;11:12158. doi: 10.1038/s41598-021-91717-y (PMC8190066; doi:10.1038/s41598-021-91717-y)
Supplement: Supplementary file 3 — Supplementary Information 3. [file 41598_2021_91717_MOESM3_ESM.pdf]

SUPPLEMENTARY MATERIAL FOR

FIRST RECORD OF A TOMISTOMINE CROCODYLIAN FROM AUSTRALIA

SUPPLEMENTAL DOCUMENT S3: COMPUTED TOMOGRAPHIC SCANNING SETTINGS AND PARAMETERS FOR QMF14.548

by JORGO RISTEVSKI[1*], GILBERT J. PRICE[2], VERA WEISBECKER[1, 3] and STEVEN W. SALISBURY[1]

[1]School of Biological Sciences, The University of Queensland, Brisbane, 4072, Queensland, Australia

[2]School of Earth and Environmental Sciences, The University of Queensland, Brisbane, 4072, Queensland, Australia

[3]College of Science and Engineering, Flinders University, Bedford Park 5042, South Australia, Australia

***Corresponding Author:**

Jorgo Ristevski[1]

School of Biological Sciences, Goddard Building (Building 8), The University of Queensland, Brisbane 4072, Queensland, Australia

Email address: j.ristevski@uq.net.au

CONTENTS OF THE SUPPLEMENTARY DOCUMENT

In this supplementary document to the study titled "First record of a tomistomine crocodylian from Australia" are given the Computed Tomographic (CT) scanning settings and parameters for the *Gunggamarandu maunala* gen. et sp. nov. holotype specimen, QMF14.548.

**Metadata**	
Scanning date	28/11/2018
Institution name	Mater Health Services, Brisbane
Modality	CT
CT scanner model	Toshiba Aquilion One
Slice thickness	0.5 mm
Kilovoltage peak	135 kVp
Data collection diameter	320
Reconstruction diameter	320
Gantry detector tilt	0
Table height	163
Exposure time	1000
X ray tube current	530
Exposure	530
Filter type	MEDIUM
Focal spots	1.6\1.5
Single collimation width	0.5
Total collimation width	40
Samples per pixel	1
Rows	512
Columns	512
Bits allocated	16
Bits stored	16

INSTITUTIONAL ABBREVIATION

QM, Queensland Museum, Brisbane, Queensland, Australia (F, fossil)
